# Optimizing antibiotic use in Indonesia: A systematic review and evidence synthesis to inform opportunities for intervention

**DOI:** 10.1016/j.lansea.2022.05.002

**Published:** 2022-05-26

**Authors:** Ralalicia Limato, Gilbert Lazarus, Puck Dernison, Manzilina Mudia, Monik Alamanda, Erni J. Nelwan, Robert Sinto, Anis Karuniawati, H. Rogier van Doorn, Raph L. Hamers

**Affiliations:** a Eijkman-Oxford Clinical Research Unit, Jakarta, Indonesia; b Centre for Tropical Medicine and Global Health, Nuffield Department of Medicine, University of Oxford, Oxford, UK; c Faculty of Medicine, Universitas Indonesia, Jakarta, Indonesia; d Faculty of Health Sciences, Radboud University Medical Centre, Nijmegen, the Netherlands; e Department of Internal Medicine, Division of Infectious Diseases, Cipto Mangunkusumo National General Hospital, Jakarta, Indonesia; f Cipto Mangunkusumo National General Hospital, Jakarta, Indonesia; g Oxford University Clinical Research Unit, Hanoi, Vietnam

**Keywords:** Antibiotic use, Antibiotic consumption, Antimicrobial stewardship, Indonesia, Systematic review, Meta-analysis

## Abstract

**Background:**

A major driver of antimicrobial resistance (AMR) and poor clinical outcomes is suboptimal antibiotic use, although data are lacking in low-resource settings. We reviewed studies on systemic antibiotic use (WHO ATC/DDD category J01) for human health in Indonesia, and synthesized available evidence to identify opportunities for intervention.

**Methods:**

We systematically searched five international and national databases for eligible peer-reviewed articles, in English and Indonesian, published between 1 January 2000 and 1 June 2021 including: (1) antibiotic consumption; (2) prescribing appropriateness; (3) antimicrobial stewardship (AMS); (4) consumers’ and providers’ perceptions. Two independent reviewers included studies and extracted data. Study-level data were summarized using random-effects model meta-analysis for consumption and prescribing appropriateness, effect direction analysis for antimicrobial stewardship (AMS) interventions, and qualitative synthesis for perception surveys. (PROSPERO: CRD42019134641)

**Findings:**

Of 9323 search hits, we included 100 reports on antibiotic consumption (20), prescribing appropriateness (49), AMS interventions (13), and/or perception (25) (8 categorized in >1 domain). The pooled estimate of overall antibiotic consumption was 134.8 DDD per 100 bed-days (95%CI 82.5–187.0) for inpatients and 121.1 DDD per 1000 inhabitants per day (10.4-231.8) for outpatients. Ceftriaxone, levofloxacin, and ampicillin were the most consumed antibiotics in inpatients, and amoxicillin, ciprofloxacin, and cefadroxil in outpatients. Pooled estimates for overall appropriate prescribing (according to Gyssens method) were 33.5% (18.1–53.4) in hospitals and 49.4% (23.7–75.4) in primary care. Pooled estimates for appropriate prescribing (according to reference guidelines) were, in hospitals, 99.7% (97.4–100) for indication, 84.9% (38.5-98.0) for drug choice, and 6.1% (0.2–63.2) for overall appropriateness, and, in primary care, 98.9% (60.9-100) for indication, 82.6% (50.5–95.7) for drug choice and 10.5% (0.8–62.6) for overall appropriateness. Studies to date evaluating bundled AMS interventions, although sparse and heterogeneous, suggested favourable effects on antibiotic consumption, prescribing appropriateness, guideline compliance, and patient outcomes. Key themes identified in perception surveys were lack of community antibiotic knowledge, and common non-prescription antibiotic self-medication.

**Interpretation:**

Context-specific intervention strategies are urgently needed to improve appropriate antibiotic use in Indonesian hospitals and communities, with critical evidence gaps concerning the private and informal healthcare sectors.

**Funding:**

Wellcome Africa Asia Programme Vietnam.

## Abstrak

**Latar belakang:**

Pendorong utama resistensi antimikroba/*antimicrobial resistance* (AMR) dan keluaran klinis yang buruk adalah penggunaan antibiotik yang kurang optimal meskipun data tentang masalah ini masih kurang di area yang bersumber daya rendah. Kami menelaah berbagai penelitian tentang penggunaan antibiotik sistemik (WHO ATC/DDD kategori J01) untuk kesehatan manusia di Indonesia, dan mensintesis bukti yang ada guna melihat peluang untuk intervensi.

**Metode:**

Kami mencari secara sistematis pada lima pangkalan data internasional dan nasional, artikel yang memenuhi kriteria kelayakan ditelaah mitra bestari (*peer-reviewed*), ditulis dalam bahasa Inggris dan Indonesia, serta diterbitkan antara tanggal 1 Januari 2000 sampai 1 Juni 2021, yang mencakup: 1) konsumsi antibiotik; 2) ketepatan peresepan; 3) penatagunaan antimikroba/*antimicrobial stewardship* (AMS); 4) persepsi konsumen dan penyedia layanan. Dua penelaah secara independen memilih artikel dan melakukan ekstraksi data dari artikel yang dipilih. Analisis data dilakukan dengan menggunakan model meta-analisis efek-acak (*random-effect model*) untuk melihat kesesuaian konsumsi dan peresepan, analisis arah efek (*effect direction analysis*) untuk intervensi AMS, serta sintesis kualitatif untuk survei persepsi. (PROSPERO: CRD42019134641)

**Hasil:**

Dari 9323 hasil pencarian, kami memasukkan 100 laporan tentang konsumsi antibiotik (20), ketepatan peresepan (49), AMS (13), dan/atau persepsi (25) (8 dikategorikan ke dalam >1 domain). Estimasi gabungan (*pooled estimate*) konsumsi antibiotik adalah 134,8 DDD/100 pasien-hari (KI95 82,5-187,0) untuk pasien rawat inap, dan 121.1 DDD/1000 populasi per hari (KI95 10,4-231,8) untuk pasien rawat jalan. Ceftriakson, levofloksasin, dan ampisilin merupakan antibiotik yang paling banyak dikonsumsi oleh pasien rawat inap; dan amoksisilin, siprofloksasin, and sefadroksil oleh pasien rawat jalan. Estimasi gabungan untuk peresepan yang tepat secara keseluruhan (menurut metode Gyssens) adalah 33,5% (KI95 18,1–53,4) di rumah sakit dan 49,4% (KI95 23,7–75,4) di fasilitas kesehatan primer. Estimasi gabungan untuk peresepan yang tepat (menurut panduan) di rumah sakit adalah 99,7% (KI95 97,4–100) untuk indikasi, 84,9% (KI95 38,5–98,0) untuk pilihan obat, dan 6,1% (KI95 0,2–63,2) untuk kesesuaian secara keseluruhan. Pada fasilitas kesehatan primer, estimasi gabungannya adalah 98,9% (KI95 60,9–100%) untuk indikasi, 82,6% (KI95 50,5–95,7) untuk pilihan obat, dan 10,5 (KI95 0,8–62,6) untuk kesesuaian secara keseluruhan. Studi-studi yang mengevaluasi gabungan beberapa intervensi AMS menunjukkan efek positif terhadap konsumsi antibiotik, ketepatan resep, kepatuhan terhadap panduan, dan keluaran pasien. Tema utama yang diidentifikasi dalam survei persepsi adalah kurangnya pengetahuan antibiotik di antara konsumen dan penggunaan antibiotik tanpa resep.

**Interpretasi:**

Strategi intervensi spesifik konteks sangat dibutuhkan untuk meningkatkan penggunaan antibiotik yang tepat di rumah sakit dan masyarakat di Indonesia, dengan kesenjangan bukti kritis tentang penyedia layanan kesehatan swasta dan informal.

**Pendanaan:**

Wellcome Trust Africa Asia Programme Vietnam.

**Research in context**

**Evidence before this study**

We reviewed government resources, situation analysis reports, websites of key global and government agencies, and searched PubMed with the terms “Indonesia”, “antibiotic use”, and “antibiotic consumption”, until December 1^st^ 2021, in English and Indonesian language. Indonesia, and the Southeast Asian region, is regarded a global hotspot for the emergence and spread of antimicrobial resistance (AMR) because of dense populations, unprecedented, yet uneven, economic development, fragile health systems with variations in access to quality health care, high infectious disease burdens, and weakly enforced antibiotic policies. Indonesia has seen an estimated 2.5-fold increase in nation-wide antibiotic consumption between 2000 and 2015, based on pharmaceutical sales data, mostly driven by broad-spectrum penicillins, fluoroquinolones and cephalosporins. Representative contemporary data on antibiotic use are lacking, although available data suggest antibiotic overuse in the healthcare system, widespread over-the-counter use in communities, and high levels of AMR among common Gram-negative bacteria. A comprehensive review on antibiotic use in human health in Indonesia has not been conducted to date.

**Added value of this study**

This review represents a first attempt at systematically assessing the peer-reviewed literature, including English and Indonesian language publications, on human antibiotic use in Indonesia spanning the past 20 years. This evidence synthesis provides a reference document, providing important insights in the magnitude, patterns and drivers of antibiotic use, as well as identifying areas where critical information is lacking. Available data suggested that only 34 and 49% of antibiotic prescriptions were appropriate in hospital and primary care settings, respectively, although the quality of the evidence was low. Studies to date evaluating bundled AMS interventions, although sparse and heterogeneous, suggested favourable effects on antibiotic consumption, prescribing appropriateness, guideline compliance, and patient outcomes. Perception surveys among healthcare providers and communities suggested important gaps in community antibiotic knowledge, and that non-prescription antibiotic self-medication is common practice, although data on health system-level drivers of antibiotic use were lacking.

**Implications of all available evidence**

There are critical evidence gaps on antibiotic use in the informal and formal private health care sector as well as geographic areas outside of Java Island, and what are health system drivers of antibiotic use. There is a need to strengthen the local research base to develop context-specific sustainable AMS models that consider country-specific socio-cultural circumstances. Optimisation of antimicrobial use as a means to tackle AMR and improve the treatment of bacterial infections, should be a priority of the national agenda for universal health coverage.

Alt-text: Unlabelled box

## Introduction

The global rise in antimicrobial resistance (AMR) is one of the greatest public health threats, with a disproportionate impact in low- and middle-income countries (LMICs).^1^ A recent global analysis estimated that AMR was directly responsible for 1.27 million deaths and played a part in 4.95 million deaths in 2019 worldwide, including 97 000 and 369 000, respectively, in the Southeast Asian region.^1^ One of the major drivers of AMR is antibiotic use, including their overuse and misuse.^2^^,^^3^ In low-resource settings, lack of access to quality healthcare, vaccination, safe water and sanitation, leave many people vulnerable to infection and dependent on antibiotics for treatment, with their use largely unregulated. Globally, during the past decade concerted efforts have been made to develop strategies to preserve the effectiveness of existing antibiotic agents.

Indonesia is a lower-middle-income country in Southeast Asia with the world's fourth largest population (274 million), and socio-economic conditions and health indicators vary widely across the archipelago. More than 55% of the population is concentrated on Java Island, which has the best developed health infrastructure.^4^ Indonesia has a decentralised public healthcare system, in which provincial or district-level governments have the authority over most public hospitals,^5^ and a substantial private health sector.^6^ In 2020, Indonesia had a total of 2985 hospitals, 21 550 primary health centres, and an estimated 135 000 drug outlets in the community selling over-the-counter drugs, of which only 29% were officially licensed pharmacies and drug stores.^7^^,^^8^ To achieve the goal of universal healthcare coverage, in 2014 the Government introduced national health insurance (*Jaminan Kesehatan Nasional*),^4^ which had reached 84% of the population by 2021. Based on an analysis of pharmaceutical sales data in 76 countries between 2000 and 2015, Indonesia ranks among the greatest risers in antibiotic consumption (29th).^3^ A range of complex factors, including variable access to quality health care, persistently high infectious disease burdens, and weakly enforced antibiotic policies, render Indonesia particularly vulnerable to AMR.^9^^,^^10^ The implementation of the National Action Plan for AMR since 2017^11^^,^^12^ has been hindered due to, among other factors, a limited evidence base of AMR epidemiology, antibiotic utilisation and rational prescribing practices.^9^ To our knowledge, there has been no comprehensive countrywide analysis to date of the magnitude and key drivers of AMR and antibiotic use,^5^ which is critical to generate evidence and highlight gaps that can help guide priorities of the National Action Plan for AMR.^13^

We therefore systematically reviewed the scientific literature on antibiotic use for human health, in both hospital and primary care settings, in Indonesia spanning the past 20 years. This Review focused on four key domains: (1) antibiotic consumption; (2) appropriateness of antibiotic prescribing; (3) AMS interventions; (4) knowledge, attitudes and perceptions among consumers and providers. This Review also reflects on current progress in the National Action Plan for AMR, and defines evidence gaps and context-specific priorities for action.

## Methods

### Search strategy and selection criteria

This systematic review was reported according to the Preferred Reporting Items for Systematic Reviews and Meta-analyses (PRISMA) 2020 guidelines.^14^ A protocol has been prospectively registered in the PROSPERO database (CRD42019134641). We screened five international (PubMed, EMBASE and Google Scholar) and national (Garuda [Garba Rujukan Digital] and Neliti) databases for peer-reviewed original articles from Indonesia, published in English or Indonesian between 1 January 2000 and 1 June 2021 using search terms listed in **Table S1**. Reference screening was used to identify additional relevant papers. The review was limited to antibacterials for systematic human use (J01 category in WHO ATC/DDD index). We did not contact study authors.

We categorised the included reports within one or more of the following domains as follows:

1. Antibiotic consumption, expressed as WHO-defined defined daily dose (DDD) per 100 bed-days (inpatients) or 1000 inhabitants per day (outpatients) (or with alternative units of measure that could be converted). We decided post-hoc to exclude paediatric studies because all studies incorrectly used DDD instead of days of therapy (DOT), as advised by WHO (dose recommendations may differ based on age and weight).^15^
2. Audits of antibiotic prescribing appropriateness for treatment or prophylaxis, based on either Gyssens method or compliance with reference guideline(s). According to the Gyssens method,^16^^,^^17^ an expert panel of at least two reviewers sequentially evaluates each prescription with related clinical and microbiological data in the medical record and local guidelines, based on seven pre-defined indicators: insufficient data (vi); antibiotic is not indicated (v); alternative antibiotic is available (iv) that is more effective (iv-a), less toxic (iv-b), less costly (iv-c), has narrower spectrum (iv-d); inappropriate duration (iii), either too long (iii-a), or too short (iii-b); incorrect dose (ii-a), interval (ii-b), route (ii-c); incorrect timing (i); and appropriate use (0). Compliance with specified reference guideline(s) will be recorded as assessed by the study investigators, based on at least one of eight pre-defined indicators: no contra-indication or allergy label, indication, drug choice, dose, frequency, duration, route of administration, and overall appropriate use. Point prevalence surveys were also included in this category as relevant (e.g., GLOBAL-PPS, WHO-PPS).
3. AMS intervention evaluation studies, with a clearly described pre-post or (quasi-) experimental design and outcome measures.
4. Surveys assessing knowledge, attitudes and/or perceptions on antibiotic use, including factors related to health system, health care providers and/or consumers.

We excluded non-human studies; studies exclusively describing other (non-J01) drugs; describing or comparing the effectiveness, cost, quality and/or molecular profiles of antibiotics; exclusively targeting particular diagnoses and its treatment; non-peer-reviewed papers, student theses, conference proceedings, and studies with irretrievable full-text. Because the included studies were mostly neither randomised controlled studies nor comparative studies, traditional methods for assessment of risk of bias were not applicable. To ensure quality, we excluded studies that did not report an essential set of STROBE checklist core items,^18^ or did not fulfil additional criteria (**Table S2**). All steps in this systematic literature search were conducted using Mendeley reference management software. Two reviewers (PD, MM) separately screened all titles and abstracts, and removed duplicates. Full-text articles were independently judged for relevance and quality by at least two reviewers (PD, MA, MM, RL). Any disagreements were resolved by a senior researcher (RLH).

### Data analysis

We extracted and tabulated data on study design, location, size, population, health care setting, year of study, indicators, interventions, outcome measures and effects, and emerging themes, as appropriate and relevant, based on the author-reported summary estimates (not individual patient-level data). Data were extracted by two reviewers (PD, MM) onto a predesigned form.

Where possible, data on antibiotic consumption and appropriateness of antibiotic prescribing were pooled with random-effects meta-analyses. Data on antibiotic consumption were pooled with meta-analyses of rates using the generic inverse variance method, with DDD per 100 bed-days for inpatients and DDD per 1000 inhabitants per day (DID) for outpatients as the rates, and sample sizes as the denominator. Furthermore, we also reported the top-15 antibiotics with the highest DDDs for inpatients and outpatients, provided that the antibiotic was reported in at least two studies, and grouped them according to the 2021 WHO AwaRe (Access, Watch, and Reserve) classification.^19^ For outpatients, due to the limited number of antibiotics, we reported all antibiotics reported in at least two studies. Data on the appropriateness of antibiotic prescribing, separate for Gyssens method (i.e. “appropriate use”, indicator 0) and reference guidelines (i.e. “overall appropriate”), were pooled with meta-analyses of proportions using the generalised linear mixed model with the logit transformation.^20^ Heterogeneity was assessed using I^2^ statistic (low <25%, moderate 25–49%, substantial 50–74%, or high 75–100%) and chi-squared test (*p* < 0.10). When two or more studies involved overlapping populations, data synthesis was prioritised to studies with larger sample sizes. Subgroup analyses were then performed by dichotomising the studies based on health care setting (primary care vs hospital [including secondary and tertiary levels]), study location, year of study, AWaRe classification (for DDD only), and age groups (for appropriateness only). We also performed sensitivity analyses by sequentially excluding individual studies (for DDD and Gyssens), and by replicating the analysis using other transformations and without transformation (for Gyssens only). All meta-analyses were performed in R version 4.1.0. When appropriate (k≥10), publication bias assessments were performed by visually inspecting funnel plots, generated by plotting the inverse square root of study sizes against the effect estimates,^21^ and by Egger's tests (*p* < 0.10).

Due to substantial clinical heterogeneity in studies evaluating AMS interventions, we synthesised the findings using a vote-counting method based on effect direction. The interventions were classified as structural, enabling, persuasive, restrictive, or combined (bundled).^22^ The perception surveys were qualitatively synthesised based on the main emerging themes, stratified by consumers (community) and healthcare providers.

### Role of the funding resource

The funder of the study had no role in study design, data collection, data analysis, data interpretation, or writing of the report.

## Results

### Study characteristics

**Figure 1:**
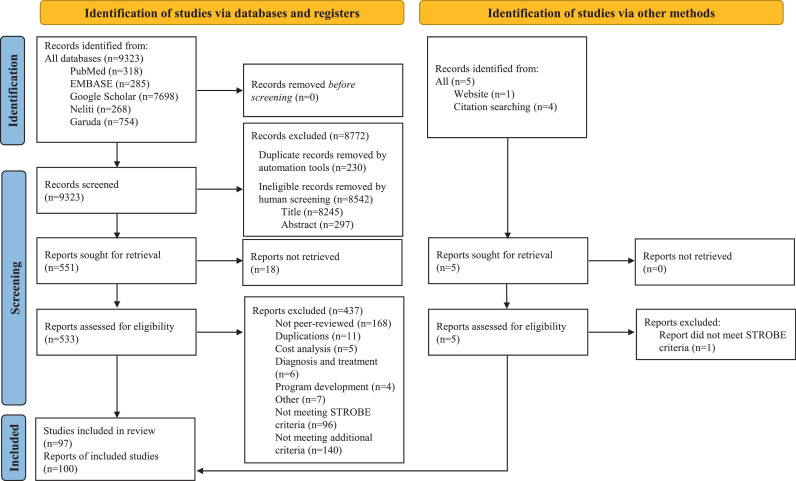
PRISMA flowchart of study selection.

**Figure 2:**
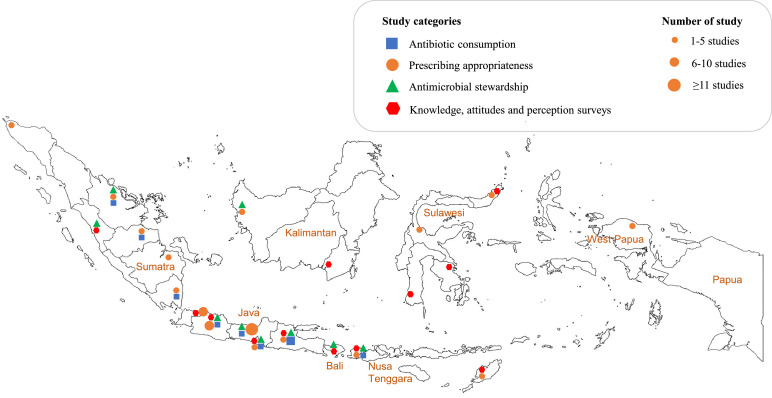
Geographical map of the 100 included reports on antibiotic use in Indonesia 2000–2021. The map includes 2 KAP surveys that were conducted nationwide, and 1 AMS study and 1 KAP survey that were conducted in multiple provinces. Editor note: The Lancet Group takes a neutral position with respect to territorial claims in published maps and institutional affiliations.

The search strategy collectively gave 9 323 hits. After title and abstract screening and duplicate removal, 551 articles remained (Figure 1). After full-text screening and quality assessment, 100 reports (covering 97 studies) were included. Study characteristics are summarized in Tables 1 and S3. 86.0% (86/100) of reports had an observational design, 13.0% (13/100) were pre-post intervention comparisons, and 1.0% (1/100) was a randomized study. Most reports were from the public sector (58.4%, 59/101). Most (53.0%, 53/100) studies were conducted in hospital settings followed by primary care (24.0%, 24) or in the community (17.0%, 17) (6 reports provided no information). The study populations were mostly patients (68.6%, 74/108), followed by communities (18.5%, 20/108) and health care providers (12.9%, 14/108). Most reports originated from Java (60.2%, 62/103), followed by Sumatra (15.5%, 16/103), Papua and Nusa Tenggara (7.8%, 8/103), Sulawesi (6.8%, 7/103), Kalimantan (5.8%, 6/103) and Bali (1.9%, 2/103) (Figure 2). 62.0% (62/100) of all reports were published during the most recent five years (2016–2021), 29.0% (29/100) during 2011–2015, and only 9.0% (9/100) during 2000–2010.

**Table 1:** Characteristics of included reports.

Total reports	N	%
	100	
**Domains**	**107**	
Antibiotic consumption	20	18.7
Appropriateness of antibiotic prescribing	49	45.8
Gyssens method	18	16.8
Reference guidelines	31	29.0
Antibiotic stewardship	13	12.1
Knowledge, attitude and practice survey	25	23.4
**Study design**	**100**	
Observational	86	86.0
Pre-post design	13	13.0
Randomized control trial	1	1.0
**Study population**	**108**	
Patients	74	68.6
Inpatients	56	51.9
Outpatients	18	16.7
Community	20	18.5
Healthcare providers a	14	12.9
**Study location**	**103**	
Java	62	60.2
Sumatra	16	15.5
Papua and Nusa Tenggara	8	7.8
Sulawesi	7	6.8
Kalimantan	6	5.8
Bali	2	1.9
National	2	1.9
**Year of study**	**100**	
2016-2021	62	62.0
2011-2015	29	29.0
2000-2010	9	9.0
**Healthcare level**	**100**	
Primary care	24	24.0
Hospitals^b^	53	53.0
Not applicable c	17	17.0
No information provided	6	6.0
**Health care sector**	**101**	
Public	59	58.4
Private	19	18.8
Not applicable (community)	19	18.8
No information provided	4	4.0

Total N for some characteristics are greater than N=100 because some reports were included in more than category, i.e. for domain (7 reports), population (8), location (3), and health care sector (1).

a Physicians (10 reports), pharmacists (1 report), mix of physicians, nurses, paramedics, and pharmacists (3 reports).

b Hospitals included secondary and tertiary levels.

c Studies conducted in the community (11 reports) and pharmacies (6 reports).

### Antibiotic consumption

**Figure 3:**
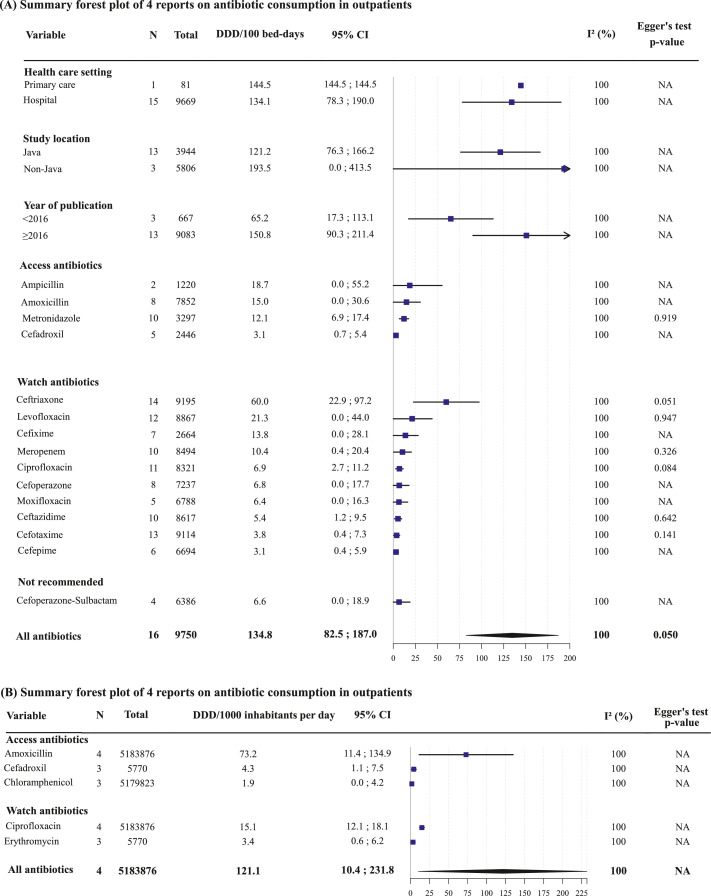
Summary forest plot of reports on antibiotic consumption in inpatients and outpatients. The figure summarizes (A) 16 inpatient reports and (B) 4 outpatient reports in the domain antibiotic consumption, expressed as DDD/100 bed-days (for inpatients) or DDD/1000 inhabitants per day (for outpatients) for all antibiotics that were reported in at least two studies, listing here up to 15 antibiotics (“top-15”). The antibiotics were grouped according to the 2021 WHO AwaRe (Access, Watch, and Reserve) classification. We noted some discrepancies between the 2021 WHO AWaRe classification and the 2021 Indonesian Ministry of Health AWaRe classification (Kementerian Kesehatan Republik Indonesia 2021); erythromycin and ciprofloxacin were classified as Watch vs Access; cefoperazone-sulbactam as Not recommended vs Watch; and cefepime and meropenem as Watch vs Reserve, respectively. Egger's tests to assess publication bias could only be performed for the primary analysis and the individual AWaRe antibiotics. Abbreviations: CI, confidence interval; DDD, defined daily dose; NA, not applicable.

There were 20 reports (5 193 626 patients) that reported data on antibiotic consumption, of which 16^23^, ^24^, ^25^, ^26^, ^27^, ^28^, ^29^, ^30^, ^31^, ^32^, ^33^ in inpatients and four^34^, ^35^, ^36^, ^37^ in outpatients. Most of these studies were conducted in hospital settings (16 reports, 76.2%), in Java (14 reports, 66.7%), and between 2016-2021 (17 reports, 81.0%; Table S4). The pooled estimate of overall antibiotic consumption was 134.8 DDD per 100 bed-days (95% confidence interval [CI] 82.5-187.0; I^2^=100%; Figure S1) for inpatients and 121.1 DID (95%CI 10.4-231.8; I^2^=100%; Figure S2) for outpatients. Among inpatients, ceftriaxone was the most consumed antibiotic (60.0 DDD per 100 bed-days [95%CI 22.9-97.2]), followed by levofloxacin (21.3 DDD per 100 bed-days [95%CI 0.0-44.0]) and ampicillin (18.7 DDD per 100 bed-days [95%CI 0.0-55.2]; Figure 3). Among outpatients, amoxicillin was the most consumed antibiotic (73.2 DID [95%CI 11.4-134.9]), followed by ciprofloxacin (15.1 DID [95%CI 12.1-18.1]) and cefadroxil (4.3 DID [95%CI 1.1-7.5]). Sensitivity analysis found that the pooled estimate for antibiotic consumption in inpatients was not dominated by a single report (Figure S3), while the pooled estimate for antibiotic consumption in outpatients was largely influenced by the study by Pradipta et al,^35^ suggesting a non-robust finding (Figure S4).

Among inpatients, subgroup analyses (Figure 3) showed higher antibiotic consumption in recent years (2016-2021: 150.8 DDD/100 bed-days [95%CI 90.3-211.4]) than previous years (2000-2015: 65.2 DDD/100 bed-days [95%CI 17.3-113.1]), and outside of Java (193.5 DDD/100 bed-days [95%CI 0.0-413.5]) than in Java (121.2 DDD/100 bed-days [95%CI 76.3-166.2]). Subgroup analyses for outpatients were not performed due to the limited number of studies in each subgroup which precluded a reliable estimate. Due to the lack of studies investigating outpatients (studies *n* < 10), publication bias assessment was only performed for inpatients, which showed an asymmetrical funnel plot (Egger's *p* = 0.050; Figure S5), indicating potential reporting bias or true heterogeneity in antibiotic consumption patterns between studies, hospitals and/or geographic areas.

### Appropriateness of antibiotic prescribing

There were 49 reports that reported data on the appropriateness of antibiotic prescribing, of which 18^27^^,^^38^, ^39^, ^40^, ^41^, ^42^, ^43^, ^44^, ^45^, ^46^, ^47^, ^48^, ^49^, ^50^, ^51^, ^52^, ^53^, ^54^ (3167 prescriptions) used Gyssens method and 31 (7826 prescriptions)^23^^,^^55^, ^56^, ^57^, ^58^, ^59^, ^60^, ^61^, ^62^, ^63^, ^64^, ^65^, ^66^, ^67^, ^68^, ^69^, ^70^, ^71^, ^72^, ^73^, ^74^, ^75^, ^76^, ^77^, ^78^, ^79^, ^80^, ^81^, ^82^, ^83^, ^84^ used reference guidelines. Most were conducted in hospitals (37 reports, 74.0%), in Java (33 reports, 66.0%), and in adults (29 reports, 58.0%) and between 2016-2021 (62 reports, 62.0%) (Tables S5 and S6).

**Figure 4:**
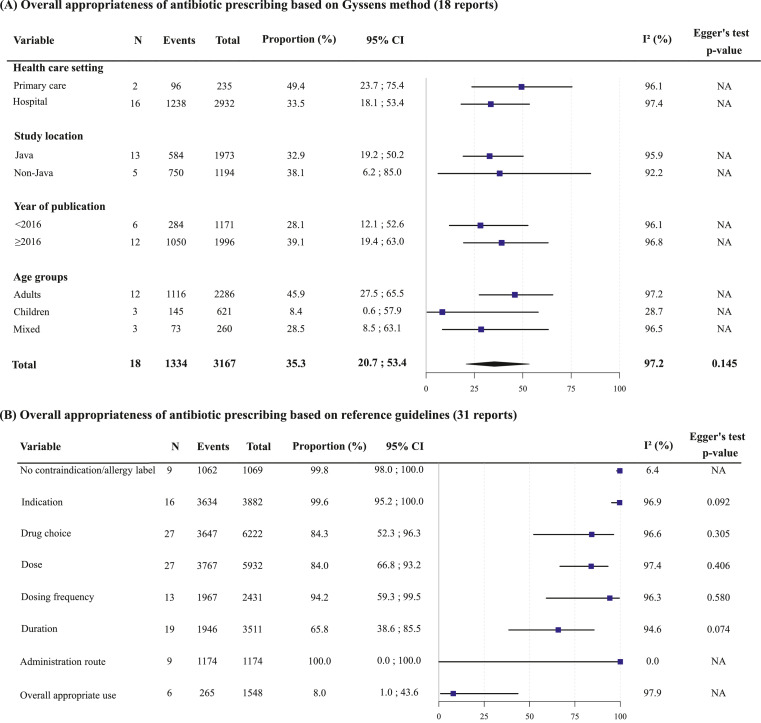
Summary forest plot on prescribing appropriateness. The figure summarizes 49 reports in the domain prescribing appropriateness based on Gyssens method (A) or references guidelines (B). The pooled proportions were weighted using a random-effects model. Indicators (B) were reported by different numbers of studies. Egger's tests to assess publication bias in Gyssens method (A) could only be performed for the primary analysis. Abbreviations: CI, confidence interval; NA, not applicable.

Based on the Gyssens method, the pooled estimate for the appropriateness of antibiotic prescribing was 35.3% (95%CI 20.7-53.4%; I^2^=97.2%) (Figure S6). Confidence analysis showed that the overall estimate was not dominated by a single study (Figure S7) and was similar between approximation methods (Figure S8). The symmetrical funnel plot (Egger's *p* = 0.108; Figure S9) indicated low risk of bias from small-study effects. Subgroup analyses showed higher appropriateness of antibiotic prescribing in adults (43.1% [95%CI 25.9-62.2%] than in children (8.4% [95%CI 0.6-57.9%]), but there were no significant differences between primary care 49.4% [95% CI 23.7-75.4%]) vs hospitals (33.5% [95%CI 18.1-53.4%]), Java (31.4% [95%CI 18.8-47.6%]) vs outside of Java (38.1% [95%CI 6.2-85.0%], and recent years (2016-2021: 39.1% [95%CI 19.4-63.0%] vs previous years (2000-2015: 26.1% [95%CI 12.4-47.1%] (Figure 4A).

Based on reference guidelines, most individual indicators of appropriateness scored excellent (>80%), except for duration (65.8% [95%CI 38.6-85.5%]) (Figure 4B) and overall appropriate antibiotic use (8.0% [95%CI 1.0-43.6%]). Between-study heterogeneity was substantial (75-100%) for all indicators, except for administration route (I^2^=0%, *p* > 0.999) and no contraindication/allergy label (I^2^=6.4%, *p* = 0.382). Possible small-study effects were found for indication and duration (Egger's test p=0.092 and p=0.074, respectively). Subgroup analysis showed higher appropriateness of duration in hospitals (80.0% [95%CI 66.0-89.2%]) than primary care (34.5% [95%CI 4.3-86.1%]), and in adults (83.2% [95%CI 65.3-92.9%] than children (17.9% [95%CI 1.2-79.4%]; Figure S10), and higher appropriateness of drug choice, dosing, and overall appropriate use outside of Java (95.2% [95%CI 73.9-99.3%], 90.0% [95%CI 71.1-97.1%], and 34.5% [95%CI 3.1-89.8%] than in Java (70.0% [95%CI 23.2-94.7%], 77.3% [95%CI 48.9-92.3%], and 3.3% [95%CI 0.3-32.0%]; Figs. S11–S13, respectively) –although the latter differences were not statistically significant. There were no significant differences for no contraindication/allergy label, indication, dosing frequency, or administration route (Figs. S14–17).

### Antimicrobial stewardship interventions

There were 13 reports that reported data on the effect of AMS interventions in hospitals (11 reports),^24^^,^^27^^,^^33^^,^^46^^,^^85^, ^86^, ^87^, ^88^, ^89^, ^90^, ^91^ primary care (1),^92^ and community pharmacies (1)^93^ (Table 2). Twelve reports used a pre-post design, and one was a randomized controlled trial. Five reports evaluated a bundled intervention and eight studies evaluated single interventions. Enabling interventions were most common (antibiotic prescribing guidelines or clinical pathway [7 reports], audit and performance feedback [3], and pharmacist counselling [1]), followed by education interventions [6], structural interventions (free blood cultures [1], integrated drug management [1]), and restrictive interventions (antibiotic restriction with pre-approval [1]). The most common reported outcome measures were changes in appropriateness of prescribing (10 reports), antibiotic consumption (8) and mortality (2), followed by prescribers’ scores for knowledge of and attitude towards antibiotic use, blood culture sampling, hand hygiene compliance, clinical outcomes, length of hospital stay, and antibiotic compliance (1 each).

**Table 2:** Summary of reports on antimicrobial stewardship interventions.

Author	Year of study	Location (city, province)	Health care setting	Study population	Study design	Intervention description	Outcome measures	Summary of study findings	Effect direction	Favourability of effect	Comments
Hadi et al (2008)^24^	2003-2004	Surabaya (East Java)	Tertiary hospital	Residents and specialists in the internal medicine department	Pre-post design	Combined (enabling, education, and structural): comprising guideline development, distribution of a guideline pocketbook, free-of-charge blood cultures, teaching sessions and refresher courses	Antibiotic consumption	Antibiotic consumption decreased from 99.8 to 73 (-26.9%) DDD/100 patient-days.	▼	(+)	The multifaceted intervention had limited success, with a very important drawback being the absence of adequate microbiological diagnostics.
							Appropriate antibiotic prescribing (Gyssens)	Appropriate antibiotic prescribing improved insignificantly from 16% to 25% overall (+9%, 95%CI -6% to 24%). Prescribing without indication insignificantly decreased from 53% to 40% (-13%, 95%CI 4% to -32%).	ᐃ	(+)	
							Guideline compliance	Guideline compliance did not change overall (from 87% to 88%), except for a significant increase for sepsis (from 49% to 72%; +23% [95%CI 4-41%]) and dengue fever (from 58% to 88%; +30% [95%CI 12-48%]).	◁▷	(+/-)	
							Blood culture sample taken	Taking blood cultures increased from 3% to 81%. However, only 3% of the blood cultures post-intervention were taken before starting antibiotic treatment.	▲	(+/-)	
							Mortality within 6 days of admission	Mortality in pre-post-period were similar (from 6.6% to 6.2%).	◁▷	(+/-)	
Murni et al (2014)^89^	2011-2013	Yogyakarta (Daerah Istimewa Yogyakarta)	Tertiary hospital	All doctors, nurses, and allied workers in the paediatric wards and PICU	Pre-post design	Combined (education and enabling): comprising educational seminars, audit and performance feedback	Primary: Proportion of patients with an HAI	Proportion of patients with HAI decreased from 22.6% (277/1227 patients) to 8.6% (123/1419 patients) (RR 0.38 [95% CI 0.31 to 0.46]).	▼	(+)	Multifaceted intervention reduced HAI rates, improved rational use of antibiotics, increased hand hygiene compliance, and reduced mortality in children
							Secondary: inappropriate antibiotic use (reference guideline), hand hygiene compliance and mortality rates	Inappropriate antibiotic use decreased from 43% to 20.6% (RR 0.46 [95%CI 0.40 to 0.55]).	▼	(+)	
								Hand hygiene compliance improved from 18.9% (319/1690) to 62.9% (1125/1789) (RR 3.33 [95%CI 2.99 to 3.70]).	▲	(+)	
								In-hospital mortality decreased from 10.4% (127/1227) to 8% (114/1419) (RR 0.78 [95%CI 0.61 to 0.9]7).	▼	(+)	
Hapsari et al (2006)^88^	2003-2004	Semarang (Central Java)	Tertiary hospital	Doctors in the paediatric ward	Pre-post design	Combined (education and enabling): comprising development of antibiotic prescribing guideline, prescriber training, and two rounds of feedback during a 6 months’ period	Antibiotic consumption	Antibiotic consumption decreased from 0.48 to 0.38 DDD/100 patient-days (*p*=0.01).	▼	(+)	
							Appropriate antibiotic prescribing (Gyssens)	Appropriate antibiotic prescribing improved from 37% to 70.4% (*p* < 0.01). Prescribing without indication did not change (from 43% to 42.7%).	▲	(+)	
Dwiprahasto (2004)^92^	1997-1998	West Kalimantan, West Sumatra, West Nusa Tenggara, East Java	Primary care	Doctors, nurses, and paramedics at 118 PHCs and provincial health office staff in 18 districts' warehouses	Pre-post design with control group	Combined (education and enabling): (1) Interactive problem-based pharmacotherapy training; (2) integrated drug management and use for provincial health office staff; followed by (3) self-monitoring of drug use at PHCs post-intervention, and monthly reporting to health office.	Proportion of patients with ARI who received an antibiotic	Significant decrease from 92.7-93.4% of patients to 64.6-72.4% after 6 months (*p* < 0.05) and 35.4-37.4% after 12 months (*p* < 0.01) post-intervention, compared to no decline in the control group (89.9-92.2%)	▼	(+)	
							The use of injection medication (analgesic or antibiotic combined with antihistamine) for myalgia	Significant decrease from 82.4-89.2% of patients to 76.2-72.5% after 6 months (*p* < 0.05) and 32.8-41.2% after 12 months (*p* < 0.01) post-intervention, compared to no significant decline in the control group (86.2-87.2%)	▼	(+)	
King & Ciptaningtyas (2015)^90^	2015	Semarang (Central Java)	Tertiary hospital	Prescribers In the digestive surgery ward	Pre-post design	Enabling: Implementation of a revised antibiotic prescribing guideline	Proportion of patients who received an antibiotic	Proportion of patients who received an antibiotic insignificantly decreased from 50% to 40.7% for therapeutic use, and from 32.5% to 29.6% for surgical prophylaxis (overall p-value = 0.410).	◁▷	(+/-)	
							Antibiotic consumption	Antibiotic consumption increased from 35.4 to 51.1 DDD/100 patient-days, mostly ceftriaxone and ciprofloxacin	ᐃ	(-)	
Rosdiana et al (2017)^46^	2016	Pekanbaru	Secondary hospital	Doctors in internal medicine ward	Pre-post design	Enabling: Development and implementation of antibiotic prescribing guideline	Appropriate antibiotic prescribing (Gyssens)	Appropriate antibiotic prescribing increased from 33.7% to 48.8% (p=0.020)	▲	(+)	
								Prescribing without indication decreased from 27.2% to 16.3% (p=0.038).	▼	(+)	
Narulita et al (2020)^86^	2018-2019	Pamekasan	Secondary hospital	Prescribers in surgery wards	Pre-post design	Enabling: Implementation of an antibiotic prescribing guideline	Antibiotic consumption	Antibiotic consumption decreased from 197.3 to 102.4 DDD/100 patient-days (p=0.065)	▽	(+)	
Lizikri et al (2020)^91^	2017-2018	No city mentioned (West Java)	Secondary hospital	Pediatricians at pediatric wards	Pre-post design	Enabling: Development and implementation of a pneumonia clinical pathway, with a patient management algorithm	Length of hospital stay (days)	Proportion of patients discharged <3 days decreased from 51.7% to 31.1% (*p*=0.065)	▽	(-)	The intervention could not achieve the targeted goals to reduce the length of hospitalization and improve antibiotic prescribing. Consumption almost doubled.
							Clinical outcomes (recovered, ICU/referred)	No discernible change in clinical outcomes as nearly all study patients had good clinical outcomes (*p*=1.00)	◁▷	(+/-)	
							Appropriate antibiotic prescribing (Gyssens)	Appropriate antibiotic use did not change from 3.3% to 3.3% (p=1.00)	◁▷	(+/-)	
							Antibiotic consumption	Antibiotic consumption increased from 222.4 to 408.4 DDD/100 patient-days (*p*=0.408)	ᐃ	(-)	
Widowati et al (2018)^93^	2018	Denpasar (Bali)	Community pharmacy	Pharmacy clients purchasing antibiotics	Randomized controlled trial	Enabling: Intervention group: counseling by a pharmacist; Control group: drug information according to the pharmacy service standard.	Antibiotic compliance was measured using the Morisky Medication Adherence Scale-8 questionnaire within 3-5 days after purchasing the medication	The proportion of compliance in the intervention group was 65.3% and the control group was 18.4% (proportion ratio 3.56, 95% CI: 1.90- 6.64).	▲	(+)	
Karuniawati et al (2021)^85^	2016-2018	Surakarta (Central Java)	Secondary hospital	Hospital-wide prescribers	Pre-post design	Enabling: Implementation of clinical practice guidelines	Antibiotic consumption	Antibiotic consumption decreased from 90.8 to 61.4 DDD/100 patient-days	▼	(+)	Shift from predominantly “red” antibiotics (mostly ceftriaxone) to “green” antibiotics (mostly ampicillin sulbactam)
							Appropriate antibiotic prescribing (Gyssens)	Appropriate antibiotic prescribing improved from 31.3% to 62.5%	▲	(+)	
								Prescribing without indication did not change (from 0% to 0%)	◁▷	(+/-)	
							Length of hospital stay (days)	Length of stay did not change significantly from 9.9 days to 10.0 days	◁▷	(+/-)	
Farida et al (2008)^87^	2003-2004	Semarang (Central Java)	Tertiary hospital	Doctors in the pediatric ward	Pre-post design	Combined (education and enabling): Prescriber training on appropriate antibiotic use (two-day training seminar) with feedback after one month	Prescribers' scores for knowledge of and attitude towards antibiotic use	Scores for knowledge of and attitude significantly increased from 59 to 77.5, and from 56 to 59, respectively.	▲	(+)	
							Appropriate antibiotic prescribing (Gyssens)	Appropriate antibiotic prescribing improved significantly from 36.3% to 58.2% (*p* < 0.01) overall, except for pneumonia (decreased) and typhoid fever (no change). Prescribing without indication significantly decreased from 42.7% to 23.3% (*p* < 0.05).	▲	(+)	
Kartika et al (2019)^27^	2018	Semarang (Central Java)	Secondary hospital	Doctors, nurses, and pharmacists in internal medicine wards	Pre-post design	Education: Antimicrobial resistance & stewardship training	Antibiotic consumption	Antibiotic consumption decreased insignificantly from 103.7 to 99.6 DDD/100 patient-days (*p*=0.092)	▽	(+)	
Susanto et al (2019)^33^	2017-2018	Pekanbaru	Secondary hospital	Hospital-wide prescribers	Pre-post design	Restrictive: Implementation of a hospital-wide antibiotic restriction programme. Pre-approval was required from the antibiotic stewardship team for third-line antibiotics	Antibiotic consumption for restricted and unrestricted antibiotics	Consumption decreased of restricted antibiotics meropenem (from 3.39 to 2.71; *p*=0.04); doripenem (from 0.42 to 0.08; *p*=0.003); imipenem (from 0.29 to 0.04; *p*=0.02); cefepime (from 1.53 to 0.28; *p*=0.001), whereas consumption of amikacin, tigecycline, vancomycin remained unchanged.	▼	(+)	
								Consumption decreased of the unrestricted antibiotics ceftriaxone (from 14.8 to 9.4; *p*=0.03) and levofloxacin (from 13.6 to 9.0; *p*=0.02). Consumption increased of the narrow-spectrum antibiotics ampicillin/sulbactam (from 1.82 to 3.66; *p*=0.003) and cefazoline (from 1.38 to 4.38; *p*=0.001)	◁▷	(+/-)	

The table summarizes 13 reports in the domain antimicrobial stewardship interventions. Findings are synthesized based on direction and favourability of effects. Antibiotic consumption is expressed as DDD/100 patient-days.

Abbreviations: ARI, acute respiratory infection; CI, confidence interval; DDD, defined daily dose; HAI, hospital-associated infection; ICU, intensive care unit; PHCs, primary health care centres; PICU, paediatric intensive care unit; RR, relative risk.

Studies that evaluated bundled interventions (5 reports) reported favourable effects on antibiotic consumption, prescribing appropriateness, guideline compliance, blood culture sampling, HAI rates, hand hygiene compliance, and mortality, although the authors of one study concluded that the multifaceted intervention had limited success, with an important drawback being the absence of adequate microbiological diagnostics.^24^^,^^87^, ^88^, ^89^^,^^92^

Studies that evaluated the implementation of antibiotic prescribing guidelines or clinical pathway (7 reports) reported mixed effects on antibiotic consumption and favourable effects on prescribing appropriateness.^24^^,^^46^^,^^85^^,^^86^^,^^88^^,^^90^^,^^91^ Studies that evaluated education interventions (6 reports) reported mixed effects on antibiotic consumption and prescribing appropriateness.^24^^,^^27^^,^^87^, ^88^, ^89^^,^^92^

The one study that evaluated antibiotic restriction with pre-approval found that consumption of restricted antibiotics decreased and of unrestricted narrow-spectrum antibiotics increased.^33^ The one randomised study that evaluated the effect of a pharmacist counselling session of outpatient antibiotic users in community pharmacies found that self-reported antibiotic adherence was significantly higher in the intervention group compared to the control group.^93^

### Knowledge, attitudes, and perceptions on antibiotic use

There were 25 reports that reported data on knowledge, attitudes, and perceptions among communities (consumers) (22)^94^, ^95^, ^96^, ^97^, ^98^, ^99^, ^100^, ^101^, ^102^, ^103^, ^104^, ^105^, ^106^, ^107^, ^108^, ^109^, ^110^, ^111^, ^112^, ^113^, ^114^, ^115^ and healthcare providers (3)^116^, ^117^, ^118^ (Tables S7 and S8). Interpretation was challenged by the considerable between-study clinical (e.g., study populations) and methodological (e.g., survey questionnaires) heterogeneity. The main themes that emerged were AMR awareness and antibiotic use knowledge, and antibiotic self-medication (including source of antibiotics, associated factors, and drivers) in the community reports and antibiotic dispensing without prescription in reports on healthcare providers.

Among community respondents, there was a substantial lack of AMR awareness (10 reports) and knowledge about antibiotics (16), with wide variations between communities; overall, 23–26% did not know that antibiotics treated bacterial infections and 58-74% stated that antibiotics can cure viral infections.^96^^,^^105^^,^^110^^,^^115^ Antibiotic knowledge was found to be associated with higher education and higher income (2).^105^^,^^111^ Further, antibiotic self-medication without prescription was reportedly common (20-100%) (9 reports). Community respondents reported they purchased antibiotics for self-medication at the pharmacy (46-90%),^94^, ^95^, ^96^^,^^99^^,^^104^^,^^106^^,^^109^^,^^112^ at the kiosk (20-44%),^96^^,^^104^ or received them from family and friends (9–12%), ^95^^,^^109^ 20–100% reported that they had ever self-medicated with an antibiotic (11),^94^, ^95^, ^96^^,^^99^^,^^102^^,^^104^^,^^106^^,^^109^^,^^112^, ^113^, ^114^ and 87–100% had ever purchased an antibiotic without a prescription (3).^104^^,^^109^^,^^112^ One study found that people without health insurance were more likely to self-medicate than those with health insurance,^111^ whereas another study reported the opposite.^104^ The main reported reasons for self-medication included positive previous experience (54–82%),^102^^,^^104^^,^^109^^,^^112^ self-medication being practical (61–83%),^94^ easy access from the pharmacy (71%),^102^ and doctor visit being expensive (44–72%)^102^^,^^109^ or unpractical (56%).^96^ The main advisors to self-medicate included health care providers (51–83%),^100^^,^^103^^,^^104^^,^^109^ family, relatives or friends (21–45%),^96^^,^^102^^,^^112^ internet (71%),^115^ or reliance on their own knowledge (71%).^96^ Antibiotic adherence levels were not associated with education level or employment status (2).^98^^,^^101^

Among health care providers, antibiotic dispensing without prescription was the most important theme reported with conflicting findings. A survey among 250 community pharmacists in Yogyakarta (Java), 68% reported that they would dispense antibiotics without prescription,^116^ whereas a survey among 110 health providers in community health centres in Padang (Sumatera) found that 98.8% did not prescribe antibiotics without a prescription, despite patient request.^117^

## Discussion

This systematic review represents a first attempt of an evidence synthesis of human antibiotic use in Indonesia, spanning the past 20 years. The evidence collected in this Review comes from a range of health care and community settings, including hospitals, primary care, pharmacies and communities, and includes a range of interventions targeting different types of health providers and consumers. The rising number of scientific reports published in the most recent years reflects the increasing momentum of AMR on the national health agenda. Nonetheless, the evidence base is uneven with hospital and urban contexts over-represented, and informal and formal private health providers, who play a major role in antibiotic distribution, particularly underrepresented.

Based on our review, most of the antibiotics listed in the top-15 most consumed antibiotics were beta-lactams, especially cephalosporins and penicillins. Among adult hospital inpatients (overall consumption 134.8 DDD per 100 bed-days [95%CI 82.5-187.0]), ceftriaxone and levofloxacin (both Watch) and ampicillin (Access) were the most consumed, and consumption was highest in the recent five years, and outside of Java. Available data among primary care outpatients were limited (overall consumption 121.1 DDD per 1000 inhabitants per day [10.4-231.8]), with amoxicillin, cefadroxil (both Access), and ciprofloxacin (Watch) being the most consumed antibiotics. According to national pharmaceutical sales data (2000-2015), antibiotic consumption increases were largely driven by the major classes broad-spectrum penicillins (2.6-fold), fluoroquinolones (7.1-fold), and cephalosporins (5.1-fold).^3^ In 2015, the antibiotic consumption rate per capita in Indonesia (3022 DDDs per 1000 inhabitants per year) fell in the same range as, for instance, China (3060) and Philippines (2600), but was still lower than, for instance, Vietnam (11 480) and Thailand (6682).^3^ About 69% of antibiotic consumption in Indonesia were Access antibiotics, which was above the WHO target of >60% Access antibiotics in total consumption.^3^ Our findings are consistent with the widespread use of broad-spectrum antibiotics, predominantly third-generation cephalosporins and fluoroquinolones, in other Asian countries^119^, ^120^, ^121^, ^122^, ^123^ and globally, and with the disproportionate rise in Watch antibiotic consumption in LMICs compared with high-income countries.^3^ Barring policy changes, antibiotic consumption is projected to increase worldwide by 200% between 2015 and 2030.^3^ The above concerning developments underscore the urgent need for regulation of antibiotic use in Indonesia, and other LMICs.

By comparison, the WHO Report on Surveillance of Antibiotic Consumption 2016-2018, representing 2015 data from 65 countries, including mostly high-income countries and no countries in Southeast Asia, found wide intra- and interregional variation in the total amount of antibiotics and the choice of antibiotics consumed,^124^ with overall consumption ranging from 4.4 to 64.4 DDD per 1 000 inhabitants per day. In the European Union, the average antibiotic consumption during 2010-2019 was 18.0 DDD per 1 000 inhabitants per day in the primary care sector, ranging from 8.7 in the Netherlands to 32.4 in Greece, and 1.8 DDD per 1 000 inhabitants per day in the hospital sector, ranging from 0.8 in the Netherlands to 2.5 in the UK.^125^ The wide variation in antibiotic consumption, both in inpatients and outpatients, is explained by differences in infectious disease burdens, health system and sector, antibiotic accessibility and regulatory policies, among many others.^126^^,^^127^

In Indonesia, among the studied populations, appropriateness of antibiotic prescribing was found to be poor overall (35.3%), 49.4% in primary care versus 33.5% in hospitals, despite nationwide implementation of hospital AMS programmes during the past five years.^9^ Several studies in primary healthcare settings in both developed and developing countries have also reported considerable rates of inappropriate antibiotic use, with a wide reported range of between 8 to 100%.^126^ In a global survey, guideline compliance of antimicrobial drug choice in hospitals in Latin America, Africa and Asia, was estimated <70% for each region.^128^ Inappropriate prescribing of antibiotics has been attributed to a range of complex factors, with variations across settings and countries, including physicians’ nonadherence to antibiotic guidelines, lack of diagnostic facilities or, where available, lack of utilization and quality, diagnostic uncertainty, pressure from pharmaceutical industry or patients.^129^^,^^130^ Around 60% of total health care spending in Indonesia is in the private sector, where financial incentives potentially promote prescribing practices that deviate from guidelines. The scale and consequences of non-prescription and private sector antibiotic consumption in Indonesia are an urgent priority for further study and action. Additionally, the local implementation of universally applicable quality indicators for antibiotic prescribing will be essential to identify targets for AMS interventions and measure their effectiveness.^131^ Point prevalence surveys to assess antibiotic use in hospitals have proven to be useful tools for identifying targets for improvement and evaluating the effect of interventions.^128^^,^^132^

Reports from high-income settings have shown that AMS can optimise the use of antimicrobials, improve patient outcomes, reduce AMR and health-care-associated infections, and save health-care costs amongst others.^22^^,^^133^ In Indonesia, AMS programmes are typically in an early stage of implementation,^134^^,^^135^ with many hospitals lacking the basic infrastructure to adequately measure process, outcome and structural indicators, e.g., access to microbiology services, hospital antibiotic guidelines, AMS staff training and education, human resources (including infectious disease specialist or clinical microbiologist), and information and communication technology support.^136^^,^^137^ Whereas to date government policy has focused on assessing hospital antibiotic use as an AMS outcome indicator (using DDD and Gyssens method),^137^ a recently launched national guideline incorporated additional outcome measures, such as cost-effectiveness, mortality, and AMR rates.^138^

The few AMS intervention studies conducted to date reported clear benefits from implementing bundled interventions combining antibiotic prescribing guidelines, trainings, review and feedback, restriction with pre-approval, among others. These findings demonstrate that AMS interventions are feasible in the local context and that there is considerable potential for reducing antibiotic consumption, particularly of restricted antibiotics, improving prescribing appropriateness, and reducing prescriptions without indication. However, the interventions evaluated to date were mostly single-centre and short-term (<1 year), and data are lacking about the sustained benefits of AMS programmes in the Indonesian context.

Previous evidence has shown that given the varying priorities and contextual issues in LMICs, such as health system processes, patient demands, varying cultures of care, availability of universal access to quality antimicrobials, laboratory infrastructure and surveillance systems, multipronged interventions combining different restrictive and enabling strategies are most likely to be effective.^139^^,^^140^ Indeed, the available Indonesian data confirm that a stand-alone guideline distribution approach may not work.^141^ Further enhancement of post-prescription review and feedback efforts holds potential to decrease antibiotic consumption and antibiotic duration.^142^ For pre-prescription approval, local data suggested that the decreased use of last-resort antibiotics might cause a “squeezing the balloon” phenomenon –the increased use of non-restricted antibiotics due to restrictions of the restricted antibiotics.^143^ The challenge for stewardship teams lies in selecting locally tailored change interventions based on a careful assessment of context-specific barriers and facilitators.^144^ Limited progress will be made with implementing AMS in Indonesian hospitals, and in settings with similar structural features, without addressing a considerable spectrum of challenges and constraints, such as lack of sustainable management-level support, competing interests and profit generation in the private sector, limited national insurance reimbursements, and limited functionality of enabling AMS infrastructures.^145^, ^146^, ^147^

Key themes identified from perception surveys were a substantial lack of AMR awareness and knowledge about antibiotics, particularly among the poor and lower educated, widespread antibiotic self-medication without prescription, and over-the-counter non-prescription antibiotic dispensing in community pharmacies −although representative data are lacking to quantify these issues. A recent mixed-method study in drug outlets in urban (West Java) and rural (South Kalimantan) settings in Indonesia reported antibiotic dispensing without prescription in 69% of simulated-patient visits, with non-prescription antibiotic sales being driven by strong patient demand, unqualified drug sellers dispensing medicines, business interests, and weak enforcement of regulations.^148^ The available data corroborate findings of a global review in community pharmacies that over-the-counter non-prescription antibiotics comprised 62% of all dispensed antibiotics, most commonly for urinary and upper respiratory tract infections.^149^ A review in Southeast Asia reported a prevalence of self-medication with antibiotics ranging from 7.3 to 85.6% (median 42.6%), highest among men, health students and professionals, with the most common illnesses or symptoms being common cold, sore throat, fever, gastrointestinal and respiratory diseases.^150^ In our review, the main reasons for self-medication included positive previous experience, easy access from the pharmacy, doctor visit being expensive or unpractical, and the main advisors to self-medicate included health care providers, family, relatives or friends, which largely concurred with findings in other Southeast Asian countries.^150^

The data included in this Review highlighted several critical evidence gaps. First, there were important limitations in data heterogeneity and study methodology, study-level confounding, and publications were predominantly from Java Island, which limited our ability to draw firm conclusions on the contemporary nationwide antibiotic use situation in Indonesia. This is especially relevant given the substantial within-country variations in access to quality health care^4^, and therefore, the data should be interpreted cautiously. Representative, high-quality data will be essential for benchmarking between healthcare facilities, districts, provinces, and internationally. Second, the exclusion of non-peer-reviewed, grey literature meant that we might not have included evidence on successful interventions implemented by government and/or non-government institutions. However, their exclusion likely improved the quality of the evidence as grey literature may not always follow gold-standard or recommended guidelines for evaluation.^151^ Third, apart from the key themes from perception surveys among antibiotic prescribers and consumers, there was a gap in the literature analysing the broader health system drivers of antibiotic use, especially concerning the effects of the national health insurance roll-out since 2014, enforcement of antibiotic regulations in informal and private health sectors, as well as on models of community antibiotic stewardship.

Progress in implementing Indonesia's National Action Plan on AMR to date includes strengthened national capacities for microbiological laboratories and surveillance,^13^ supported by the government and international funding agencies. Indonesia enrolled in the WHO Global Antimicrobial Resistance and Use Surveillance System (GLASS) and submitted first batches of AMR and antibiotic consumption data in 2020.^152^ In 2021, the Ministry of Health launched new guidelines for antibiotic prescribing and AMS programmes,^138^ which have also adopted the AWaRe classification. Despite this progress, however, the National Action Plan for AMR has not yet generated the required sustainable capacity to contain AMR. A recent cross-country analysis of the current national action plans for AMR in Southeast Asia, including Indonesia and 9 other ASEAN countries (The Association of Southeast Asian Nations), listed Indonesia's priorities towards optimising antibiotic usage as the development of stronger regulatory frameworks, evidence-based AMS programmes in ambulatory and community settings and as part of hospital accreditation qualifying criteria, and standard antibiotic treatment guidelines, including an essential antibiotics list.^13^ Interventions to optimise antimicrobial use need to be based on a health systems approach, beyond AMS, informed by a broad research base, including addressing the wider drivers of antibiotic use –such as inequitable burdens of ill health and fractured care cascades. Both universal health coverage and AMR require strong human-centered care with accessible health care facilities, medicines and diagnostics, with a focus on quality and equity.^4^^,^^153^^,^^154^ This requires further investments in health care infrastructure, training of health workers, community participation and health literacy, at the country, province and district levels. Modifiable factors related to the patient (e.g., awareness, knowledge) and the health system (e.g., strict policies, medicine quality, financial incentives, infrastructure gaps) need to be further identified and addressed when designing context-specific interventions aimed at curtailing inappropriate antibiotic use.

In conclusion, this Review can be a guiding tool for policy-makers and academics as it summarizes the state of antibiotic use in humans in Indonesia over the past 20 years, and highlights important areas where critical information is lacking. There are critical evidence gaps on antibiotic use in the informal and formal private health care sectors as well as geographic areas outside of Java Island, and what are health system drivers of antibiotic use. There is a need to strengthen the local research base to develop context-specific sustainable AMS models that consider country-specific socio-cultural circumstances. Optimisation of antimicrobial use, based on robust surveillance data and feasible interventions, should be a priority of the national agenda for universal health coverage.

## Contributors

RLH conceptualised the study. RLH and RL obtained the funding. RL, PD and RLH designed the study protocol and data extraction instrument. PD, GL, MM, and MA collected and verified the data, overseen by RL and RLH. RL, GL and RLH performed the data analysis and had full access to all study data. RL, GL, PD, and RLH drafted the paper, with critical inputs from EJN, RS, AK and HRvD. All authors had full access to all the data in the study, critically revised the manuscript, accept responsibility to submit for publication, and gave approval for the final version to be published.

## Data sharing statement

No additional data are available.

## Declaration of interests

AK serves as the current Chair of the National AMR Committee (KPRA). HRVD serves as an Executive Board Member of The Surveillance and Epidemiology of Drug-resistant Infections Consortium (SEDRIC). The other authors declare no competing interests.

## Appendix Supplementary materials

Image, application 1

Image, application 2
