# Supplementary material for: Optimizing antibiotic use in Indonesia: A systematic review and evidence synthesis to inform opportunities for intervention
Source: Lancet Reg Health Southeast Asia. 2022 May 26;2:100013. doi: 10.1016/j.lansea.2022.05.002 (PMC10305907; doi:10.1016/j.lansea.2022.05.002)
Supplement: Supplementary file 1 [file mmc1.docx]

SUPPLEMENTARY MATERIAL

Optimizing antibiotic use in Indonesia: a systematic review and evidence synthesis to inform opportunities for intervention

TABLE AND CONTENTS

| **Supplementary Material** | **Page** |
| --- | --- |
| **Methods** | 3 |
| Table S1. Search terms | 3 |
| Table S2. Quality assessment criteria for study inclusion | 3 |
| **Results** | 4 |
| Table S3. Characteristics of included studies | 4 |
| Table S4. Summary of studies on antibiotic consumption in inpatients and outpatients | 10 |
| Figure S1. Forest plot of 16 reports on the defined daily dose (DDD) of total antibiotic use in inpatients | 12 |
| Figure S2. Forest plot of 4 reports on the defined daily dose (DDD) of total antibiotic use in outpatients | 12 |
| Figure S3. Results of leave-one-out sensitivity analysis for meta-analysis of the defined daily dose (DDD) of total antibiotic use in inpatients | 12 |
| Figure S4. Results of leave-one-out sensitivity analysis for meta-analysis of the defined daily dose (DDD) of total antibiotic use in outpatients | 13 |
| Figure S5. Funnel plot indicating evidence of publication bias (as shown by asymmetry) for meta-analysis of the defined daily dose (DDD) of total antibiotic use in inpatients | 13 |
| Table S5. Summary of studies on appropriateness of antibiotic prescribing (according to Gyssens method) | 14 |
| Table S6. Summary of studies on appropriateness of antibiotic prescribing (according to reference guidelines) | 16 |
| Figure S6. Forest plot showing results of meta-analysis on the overall appropriateness of antibiotic prescribing according to Gyssens method (18 reports) | 18 |
| Figure S7. Results of leave-one-out sensitivity analysis for meta-analysis on the overall appropriateness of antibiotic prescribing according to Gyssens method | 18 |
| Figure S8. Results of sensitivity analysis for meta-analysis on the overall appropriateness of antibiotic prescribing according to Gyssens method by using alternative methods other than generalized linear mixed model (GLMM) method | 18 |
| Figure S9. Funnel plot for the meta-analysis on the overall appropriateness of antibiotic prescribing according to Gyssens method | 19 |
| Figure S10. Summary forest plot of 19 reports on the appropriateness of antibiotic prescribing according to the “duration” indicator in the reference guidelines | 19 |
| Figure S11. Summary forest plot of 27 reports on the appropriateness of antibiotic prescribing according to the “drug choice” indicator in the reference guidelines | 20 |
| Figure S12. Summary forest plot of 27 reports on the appropriateness of antibiotic prescribing according to the “dose” indicator in the reference guidelines | 20 |
| Figure S13. Summary forest plot of 6 reports on the appropriateness of antibiotic prescribing according to the “overall appropriate use” indicator in the reference guidelines | 21 |
| Figure S14. Summary forest plot of 9 reports on the appropriateness of antibiotic prescribing according to the “no contraindication/allergy label” indicator in the reference guidelines | 21 |
| Figure S15. Summary forest plot of 16 reports on the appropriateness of antibiotic prescribing according to the “indications” indicator in the reference guidelines | 22 |
| Figure S16. Summary forest plot of 13 reports on the appropriateness of antibiotic prescribing according to the “dosing frequency” indicator in the reference guidelines | 22 |
| Figure S17. Summary forest plot of 9 reports on the appropriateness of antibiotic prescribing according to the “administration route” indicator in the reference guidelines | 23 |
| Table S7. Summary of studies on knowledge, attitudes and perceptions regarding antibiotic use | 24 |
| Table S8. Summary of findings of the knowledge, attitudes and practice surveys | 26 |
| **Reference list of all reports included in the systematic review** | 32 |

METHODS

Table S1. Search terms

| Database | Keywords |
| --- | --- |
| PubMed | ("Anti-Bacterial Agents"[Mesh] OR "Antibiotic Prophylaxis"[Mesh]) OR ((“anti-bacterial”[tiab] OR “antibiotic”[tiab] OR “antimicrobial”[tiab]) AND (“use”[tiab] OR “usage”[tiab] OR “prescription*”[tiab] OR “prescribing”[tiab] OR “consumption”[tiab] OR “dispensing”[tiab] OR “stewardship”[tiab])) AND ("Indonesia"[tiab] OR "Indonesia"[MeSH]) |
| EMBASE | (((antibiotic or antimicrobial or antibacterial) and (us* or consum* or prescri* or dispensi* or stewardship) and indonesia) not animal not environment not plant not cancer not chemotherapy not carriage not etiology not susceptib* not molecular not vaccine not probiotic).ti,ab. |
| Google Scholar | indonesia antibiotic\|antimicrobial use\|usage\|prescription\|prescribing\|consumption\|dispensing\|stewardship -animal -environment -plant -cancer -chemotherapy -carriage -etiology -susceptible -susceptibility -molecular -vaccine -probiotic |
| Neliti | (antibiotik) |
| Garba Rujukan Digital (Garuda) | antibiotik (in title) |

Table S2. Quality assessment criteria for study inclusion

| No | Items | Antibiotic consumption | Antibiotic appropriateness | Antimicrobial stewardship | Knowledge, attitudes, practices |
| --- | --- | --- | --- | --- | --- |
| STROBE checklist core items | | | | | |
| 1 | Presents key elements of study design early in the paper (item 4) | ● | ● | ● | ● |
| 2 | Describe the setting, locations, and relevant dates, including periods of recruitment, exposure, follow-up, and data collection (item 5) | ● | ● | ● | ● |
| 3 | Give the eligibility criteria, and the sources and methods of selection of participants (item 6) | ● | ● | ● | ● |
| 4 | For each variable of interest, give sources of data and details of methods of assessment (measurement) (item 8) | ● | ● | ● | ● |
| 5 | Explain how the study size was arrived at (item 10) | ● | ● | ● | ● |
| 6 | Report numbers of individuals at each stage of study—e.g., numbers potentially eligible, examined for eligibility, confirmed eligible, included in the study, completing follow-up, and analysed (item 13) | ● | ● | ● | ● |
| 7 | Give characteristics of study participants (e.g., demographic, clinical, social) and information on exposures and potential confounders (item 14) | ● | ● | ● | ● |
| Additional theme-specific items | | | | | |
| 8 | Minimum study sample size | 50 | 50 |  | 100 |
| 9 | DDD method correctly applied ^a^ | ● |  |  |  |
| 10 | Gyssens flowchart method correctly applied (including evaluation by at least 2 independent expert reviewers) ^b^ |  | ● |  |  |
| 11 | Reference prescribing guidelines specified |  | ● |  |  |
| 12 | Survey questionnaire/questions available in paper |  |  |  | ● |
| 13 | Information that is crucial for data interpretation is missing or conflicting | ● | ● | ● | ● |

Abbreviations: DDD, defined daily dose; ^a^ Definitions and general considerations available from: <https://www.whocc.no/ddd/definition_and_general_considera/>; ^b^ Gyssens IC, J Antimicrob Chemother 1992:30:724–7; Van Der Meer JWM, Clin Microbiol Infect 2001;suppl6:12

RESULTS

Table S3. Characteristics of included studies

Table S3a. Antibiotic consumption (20 studies)

| No | Author | Year of publication | Year of Study | City, province | Island | Healthcare level | Healthcare sector | Age groups | Study population | No. of population | Study design |
| --- | --- | --- | --- | --- | --- | --- | --- | --- | --- | --- | --- |
| Inpatients | |  |  |  |  |  |  |  |  |  |  |
|  | Dirga^2,*^ | 2021 | 2017 | Lampung, Lampung | Sumatra | Secondary | Public | Adults | Internal medicine inpatients | 164 | Cross-sectional |
|  | Hadi^3,*^ | 2008 | 2003-2004 | Surabaya, East Java | Java | Tertiary | Public | Adults | Fever inpatients (pre-intervention) | 212 | Quasi-experimental |
|  | Herawati^4^ | 2019 | 2016 | Surabaya, East Java | Java | Secondary | Private | Adults | Surgery inpatients | 343 | Cross-sectional |
|  | Kartika ^5,*^ | 2019 | 2018 | Semarang, Central Java | Java | Secondary | Public | Adults | Internal medicine inpatients (pre-intervention) | 50 | Quasi-experimental |
|  | Kusuma^6^ | 2016 | 2013 | Banyumas, Central Java | Java | Secondary | Public | Adults | Ob/Gyn inpatients | 247 | Cross-sectional |
|  | Mahmudah^7^ | 2016 | 2013 | Bandung,  West Java | Java | Secondary | Private | Adults | Digestive surgery inpatients | 208 | Cross-sectional |
|  | Massey^8^ | 2021 | 2019 | Mataram, West Nusa Tenggara | Nusa Tenggara | Secondary | Public | Adults | Surgery inpatients | 323 | Cross-sectional |
|  | Muliani^9^ | 2021 | 2019 | Surabaya, East Java | Java | Secondary | Private | Adults | Surgery inpatients | 164 | Cross-sectional |
|  | Narulita^10,*^ | 2020 | 2018-2019 | Pamekasan, East Java | Java | Secondary | Public | Adults | Surgery inpatients (pre-intervention) | 200 | Quasi-experimental |
|  | Pratama^13^ | 2019 | 2016-2017 | Surabaya,  East Java | Java | Secondary | Public | Adults | Surgery inpatients | 463 | Cross-sectional |
|  | Putri^14^ | 2021 | 2016-2018 | Yogyakarta, DIY | Java | Secondary | Private | Adults | Adult inpatients with pneumonia | 251 | Cross-sectional |
|  | Rachmawati^15^ | 2020 | 2017 | Pasuruan,  East Java | Java | Secondary | Public | Adults | Internal medicine inpatients | 973 | Cross-sectional |
|  | Sholih^16^ | 2019 | 2018 | Karawang,  West Java | Java | Primary | Public | Adults | Inpatients | 81 | Cross-sectional |
|  | Susanto^17^ | 2019 | 2017-2018 | Pekanbaru, Riau | Sumatra | Secondary | Private | Adults | Inpatients (pre-intervention) | 5,319 | Quasi-experimental |
|  | Wikantiananda^19^ | 2019 | 2016 | (City NA), West Java | Java | Tertiary | Public | Adults | Intensive Care Unit inpatients | 57 | Cross-sectional |
|  | Yulia^20^ | 2017 | 2016 | Surabaya,  East Java | Java | Secondary | Private | Adults | Inpatients | 695 | Cross-sectional |
| Outpatients | |  |  |  |  |  |  |  |  |  |  |
|  | Andriani^1^ | 2020 | 2018-2019 | Jambi, Jambi | Sumatra | Primary | Public | Adults | Outpatients | 462 | Cross-sectional |
|  | Perdaka^11^ | 2020 | 2017-2018 | Jambi, Jambi | Sumatra | Primary | Public | Adults | Outpatients | 1255 | Cross-sectional |
|  | Pradipta^12^ | 2015 | 2008-2010 | Bandung,  West Java | Java | Primary | Public | Adults | Outpatients | 5,178,106 | Cross-sectional |
|  | Trisia^18^ | 2020 | 2017-2018 | Jambi, Jambi | Sumatra | Primary | Public | Adults | Outpatients | 4,053 | Cross-sectional |

*Studies included in multiple domains.

Abbreviations: NA, Not applicable/available, DIY, Daerah Istimewa Yogyakarta; Ob/Gyn, obstetrics and gynaecology

Table S3b. Prescribing appropriateness (49 studies)

| No | First author | Year of publication | | Year of study | | City, Province | | Island | | Health care level | | Healthcare sector | | Age groups | | Study population | | No. of prescription^a^ | Study design |
| --- | --- | --- | --- | --- | --- | --- | --- | --- | --- | --- | --- | --- | --- | --- | --- | --- | --- | --- | --- |
| Gyssens method | | |  | |  | |  | |  | |  | |  | |  | |  |  |  |
| 1. 1 | Adani^21^ | 2015 | | 2014 | | Semarang,  Central Java | | Java | | Primary | | Public | | Children | | Paediatric inpatients | | 173 | Cross-sectional |
| 1. 2 | Aljufri^22^ | 2021 | | 2017-2019 | | Semarang, Central Java | | Java | | Tertiary | | Public | | Adults | | Inpatients with pneumonia | | 98 | Cross-sectional |
| 1. 3 | Anggraini^23^ | 2018 | | 2014 | | Pontianak,  West Kalimantan | | Kalimantan | | Primary | | Public | | Mixed | | Typhoid inpatients | | 62 | Cross-sectional |
| 1. 4 | Hanifah^24^ | 2018 | | 2017 | | Semarang,  Central Java | | Java | | Secondary | | Private | | Mixed | | Typhoid inpatients | | 98 | Cross-sectional |
| 1. 5 | Hardiana^25^ | 2021 | | 2019 | | Jakarta, DKI Jakarta | | Java | | Tertiary | | Public | | Adults | | Inpatients with pneumonia | | 88 | Cross-sectional |
| 1. 6 | Ibrahim^26^ | 2020 | | 2019 | | Surabaya,  East Java | | Java | | Tertiary | | Public | | Adults | | Inpatients with infection | | 84 | Cross-sectional |
| 1. 7 | Inez^27^ | 2019 | | 2018 | | Tanjungpura, West Kalimantan | | Kalimantan | | Secondary | | Public | | Children | | Inpatients | | 63 | Quasi-experimental |
| 1. 8 | Kartika^5,*^ | 2019 | | 2018 | | Semarang, Central Java | | Java | | Secondary | | Public | | Adults | | Internal medicine inpatients | | 50 | Quasi-experimental |
| 1. 9 | Maakh^28^ | 2019 | | 2018 | | Atambua, East Nusa Tenggara | | Nusa Tenggara | | Secondary | | Public | | Adults | | Ob/Gyn inpatients | | 100 | Cross-sectional |
| 1. 1 | Magdalena^29^ | 2018 | | 2017 | | Pekanbaru, Riau | | Sumatra | | Secondary | | Private | | Adults | | ICU and medical ward inpatients | | 877 | Cross-sectional |
| 1. 1 | Muthoharoh^30^ | 2018 | | 2017 | | Pekalongan, Central Java | | Java | | Secondary | | Public | | Mixed | | Surgical inpatients | | 100 | Cross-sectional |
| 1. 1 | Purwaningsih^31^ | 2015 | | 2014-2015 | | Semarang,  Central Java | | Java | | Secondary | | Private | | Children | | Inpatients | | 385 | Cross-sectional |
| 1. 1 | Rosdiana^32,*^ | 2018 | | 2016 | | Pekanbaru, Riau | | Sumatra | | Secondary | | Public | | Adults | | Inpatients | | 92 | Quasi-experimental |
| 1. 1 | Setiawan^33^ | 2018 | | 2016 | | Surabaya,  East Java | | Java | | Tertiary | | Public | | Adults | | ICU inpatients | | 110 | Cross-sectional |
| 1. 1 | Sumiwi^34^ | 2014 | | 2013 | | Bandung,  West Java | | Java | | NA | | NA | | Adults | | Digestive surgery inpatients | | 344 | Cross-sectional |
| 1. 1 | Sutrisno^35^ | 2013 | | 2011-2012 | | Yogyakarta, DIY  DIY | | Java | | Tertiary | | Public | | Adults | | Pneumonia inpatients | | 57 | Cross-sectional |
| 1. 1 | Waridiarto^36^ | 2015 | | 2015 | | Semarang,  Central Java | | Java | | Tertiary | | Public | | Adults | | Orthopaedic inpatients | | 150 | Cross-sectional |
| 1. 1 | Yoanitha^37^ | 2018 | | 2016 | | Bandung,  West Java | | Java | | Tertiary | | Public | | Adults | | Ob/Gyn inpatients | | 236 | Cross-sectional |
| Reference guidelines | | | | |  | |  | |  | |  | |  | |  | |  |  |  |
|  | Andrajati^38^ | 2016 | | 2012 | | Depok, West Java | | Java | | Primary | | Public | | Mixed | | Outpatients with various indications | | 392 | Cross-sectional |
|  | Anggraini^39^ | 2020 | | 2013 | | Jakarta, DKI Jakarta | | Java | | NA | | Public | | Adults | | Pre-surgical inpatients | | 837 | Cross-sectional |
|  | Bakhtiar^40^ | 2019 | | 2018 | | Sorong, West Papua | | Papua | | Secondary | | Public | | Adults | | Post-surgical inpatients | | 83 | Cross-sectional |
|  | Benua^41^ | 2019 | | 2018 | | Poso,  Central Sulawesi | | Sulawesi | | Primary | | Public | | Mixed | | Acute respiratory infection outpatients | | 126 | Cross-sectional |
|  | Bestari^42^ | 2017 | | 2016 | | (City NA), Central Java | | Java | | Tertiary | | Public | | Children | | Paediatric pneumonia inpatients | | 90 | Cross-sectional |
|  | Dania^43^ | 2016 | | 2014 | | Yogyakarta,  DIY | | Java | | Secondary | | Private | | Adults | | Ob/Gyn inpatients receiving prophylaxis and therapeutic antibiotics | | 59 | Cross-sectional |
|  | Dewi^44^ | 2018 | | 2017 | | Jakarta, DKI Jakarta | | Java | | Tertiary | | Public | | Adults | | ICU inpatients with sepsis or septic shock | | 60 | Cross-sectional |
|  | Dewi^45^ | 2020 | | 2018 | | Jambi, Jambi | | Sumatra | | Primary | | Public | | Children | | Acute respiratory infection outpatients | | 51 | Cross-sectional |
|  | Dewi^46^ | 2020 | | 2019 | | Jambi, Jambi | | Sumatra | | Primary | | Public | | Children | | Acute respiratory infection outpatients | | 70 | Cross-sectional |
|  | Dirga^2,*^ | 2021 | | 2017 | | Lampung, Lampung | | Sumatra | | Secondary | | Public | | Adults | | Internal medicine inpatients | | 168 | Cross-sectional |
|  | Elvina^47^ | 2017 | | 2016 | | Jakarta, DKI Jakarta | | Java | | NA | | NA | | Adults | | Inpatients with pneumonia | | 96 | Cross-sectional |
|  | Fakhrunnisa^48^ | 2020 | | 2018 | | Tegal, Central Java | | Java | | Primary | | Public | | Mixed | | Acute respiratory infection outpatients | | 632 | Cross-sectional |
|  | Fithria^49^ | 2015 | | 2013 | | Semarang,  Central Java | | Java | | Secondary | | Private | | Children | | Inpatients with acute diarrhoea | | 54 | Cross-sectional |
|  | Grassella^50^ | 2019 | | 2018 | | Pontianak,  West Kalimantan | | Kalimantan | | Secondary | | Public | | Children | | Acute respiratory infection outpatients | | 340 | Cross-sectional |
|  | Harartasyahrani^51^ | 2021 | | 2020 | | Prabumulih, South Sumatra | | Sumatra | | Secondary | | Private | | Adults | | Surgery inpatients | | 119 | Cross-sectional |
|  | Hasyul^52^ | 2019 | | 2017 | | Garut, West Java | | Java | | Primary | | Public | | Mixed | | Typhoid outpatients | | 705 | Cross-sectional |
|  | Herlina^53^ | 2021 | | 2017 | | Mataram, West Nusa Tenggara | | Nusa Tenggara | | Secondary | | Public | | Adults | | Urinary tract infection inpatients | | 105 | Cross-sectional |
|  | Islam^54^ | 2017 | | 2014 | | Jakarta, DKI Jakarta | | Java | | Secondary | | Public | | Adults | | Pneumonia inpatients | | 139 | Cross-sectional |
|  | Jamiati^55^ | 2019 | | 2017 | | Gayo Lues, Aceh | | Sumatra | | Primary | | Public | | Mixed | | Outpatients | | 86 | Cross-sectional |
|  | Kaparang^56^ | 2014 | | 2013 | | Manado,  North Sulawesi | | Sulawesi | | Tertiary | | Public | | Children | | Pneumonia inpatients | | 112 | Cross-sectional |
|  | Kurniawati^57^ | 2021 | | 2019 | | Yogyakarta, DIY | | Java | | Secondary | | NA | | Adults | | Urinary tract infection inpatients | | 61 | Cross-sectional |
|  | Limato^58^ | 2021 | | 2019 | | Jakarta, DKI Jakarta | | Java | | Secondary, Tertiary | | Public, Private | | Mixed | | Inpatients receiving systemic antimicrobials | | 915 | Cross-sectional |
|  | Nawakasari^59^ | 2019 | | 2017 | | Klaten, Central Java | | Java | | Tertiary | | Public | | Adults | | Urinary tract infection inpatients | | 76 | Cross-sectional |
|  | Nyoman^60^ | 2017 | | 2015 | | Bandung, West Java | | Java | | Primary | | Private | | Children | | Acute respiratory infection outpatients | | 425 | Cross-sectional |
|  | Octavia^61^ | 2019 | | 2018 | | Lamongan, East Java | | Java | | Secondary | | Private | | Adults | | Ob/Gyn inpatients (prophylaxis) | | 54 | Cross-sectional |
|  | Ofisya^62^ | 2020 | | 2019 | | Pontianak,  West Kalimantan | | Kalimantan | | Secondary | | Public | | Mixed | | Pneumonia inpatients | | 86 | Cross-sectional |
|  | Oktaviani^63^ | 2015 | | 2014-2015 | | (City NA), Riau | | Sumatra | | Secondary | | Public | | Adults | | Ob/Gyn inpatients (prophylaxis) | | 140 | Cross-sectional |
|  | Ovikariani^64^ | 2019 | | 2019 | | Semarang, Central Java | | Java | | Primary | | Public | | Mixed | | Pneumonia outpatients | | 79 | Cross-sectional |
|  | Rusdiana^65^ | 2016 | | 2014 | | Tangerang, West Java | | Java | | Secondary | | Private | | Adults | | Ob/Gyn inpatients (prophylaxis) | | 256 | Cross-sectional |
|  | Sugiarti^66^ | 2015 | | 2014 | | Malang, East Java | | Java | | Primary | | Public | | Children | | Acute respiratory infection outpatients | | 120 | Cross-sectional |
|  | Zazuli^67^ | 2015 | | 2009 | | Bandung, West Java | | Java | | Secondary | | Private | | Adults | | Pre- and post-surgical inpatients | | 1290 | Cross-sectional |

*Studies included in multiple domains. ^a^ For reference guidelines, we reported as total number of prescriptions the highest number reported in a given domain; if no number of prescriptions was reported, we reported total number of patients.

Abbreviations: NA, Not Applicable/available; DIY, Daerah Istimewa Yogyakarta; DKI, Daerah Khusus Ibukota; Ob/Gyn, Obstetrics and gynaecology

Table S3c. Antimicrobial stewardship (13 studies)

| No | First author | Year of publication | Year of Study | City, province | Island | Healthcare level | Healthcare sector | Age groups | Study population | No. of population | Study design |
| --- | --- | --- | --- | --- | --- | --- | --- | --- | --- | --- | --- |
| 1 | Dwiprahasto^68^ | 2004 | 1997-1998 | West Kalimantan, West Sumatra, West Nusa Tenggara | Kalimantan,  Sumatra, Nusa Tenggara | Primary | Public | NA | Doctors, nurses, and paramedics | NA | Quasi-experimental |
| 2 | Farida^69^ | 2008 | 2003-2004 | Semarang,  Central Java | Java | Tertiary | Public | Children | Doctors | 22 | Quasi-experimental |
| 3 | Hadi^3,*^ | 2008 | 2003-2004 | Surabaya, East Java | Java | Tertiary | Public | Adults | Doctors | 155 | Quasi-experimental |
| 4 | Hapsari^70^ | 2006 | 2003-2004 | Semarang,  Central Java | Java | Tertiary | Public | Children | Doctors | NA | Quasi-experimental |
| 5 | Kartika^5,*^ | 2019 | 2018 | Semarang, Central Java | Java | Secondary | Public | Adults | Doctors, nurses, and pharmacists | NA | Quasi-experimental |
| 6 | Karuniawati^71^ | 2021 | 2016-2018 | Surakarta, Central Java | Java | Secondary | Public | Adults | Doctors | NA | Quasi-experimental |
| 7 | King^72^ | 2015 | 2014-2015 | Semarang, Central Java | Java | Tertiary | Public | Adults | Doctors | NA | Quasi-experimental |
| 8 | Lizikri^73^ | 2020 | 2017 | (City NA), West Java | Java | NA | Private | Children | Doctors | NA | Quasi-experimental |
| 9 | Murni^74^ | 2014 | 2011-2013 | Yogyakarta, DIY | Java | Tertiary | Public | Children | Doctors, nurses, and allied health workers | NA | Quasi-experimental |
| 10 | Narulita^10,*^ | 2020 | 2018-2019 | Pamekasan, East Java | Java | Secondary | Public | Adults | Doctors | 200 | Quasi-experimental |
| 11 | Rosdiana^32,*^ | 2018 | 2016 | Pekanbaru, Riau | Sumatra | Secondary | Public | Adults | Doctors | NA | Quasi-experimental |
| 12 | Susanto^17^ | 2019 | 2017-2018 | Pekanbaru, Riau | Sumatra | Secondary | Private | Adults | Doctors | NA | Quasi-experimental |
| 13 | Widowati^75^ | 2018 | 2018 | Denpasar, Bali | Bali | Primary, community pharmacy | NA | Adults | Pharmacy visitors | 98 | Experimental (Randomized Control Trial) |

*Studies included in multiple domains

Abbreviation: NA, Not Applicable/available; DIY, Daerah Istimewa Yogyakarta

Table S3d. Knowledge, attitudes and perception surveys (25 studies)

| No | First author | Year of publication | Year of Study | City, province | Island | Healthcare level | Healthcare sector | Study population | No. of population | Study design |
| --- | --- | --- | --- | --- | --- | --- | --- | --- | --- | --- |
| 1 | Artini^76^ | 2016 | 2014-2015 | Denpasar, Bali | Bali | NA | NA | Community respondents: medical and non-medical university students | 240 | Cross-sectional |
| 2 | Asvinigita^77^ | 2019 | 2019 | Yogyakarta, DIY | Java | NA | NA | Healthcare providers: community pharmacists | 250 | Cross-sectional |
| 3 | Djawaria^78^ | 2018 | 2014-2015 | Surabaya, East Java | Java | NA | NA | Community respondents at pharmacy | 267 | Cross-sectional |
| 4 | Fatmah^79^ | 2019 | 2018 | Mataram, West Nusa Tenggara | Nusa Tenggara | NA | NA | Community respondents: medical and non-medical university students | 400 | Cross-sectional |
| 5 | Fernandez^80^ | 2013 | 2012 | Manggarai Barat, East Nusa Tenggara | Nusa Tenggara | NA | NA | Community respondents at pharmacies | 108 | Cross-sectional |
| 6 | Fimanggara^81^ | 2016 | 2013 | Jatinangor, West Java | Java | NA | NA | Community respondents: non-medical college students | 250 | Cross-sectional |
| 7 | Fitriah^82^ | 2021 | 2019 | Banjarbaru, South Kalimantan | Kalimantan | NA | NA | Community respondents: household members | 380 | Cross-sectional |
| 8 | Hamid^83^ | 2020 | 2019 | Pangkajene and Kepulauan, South Sulawesi | Sulawesi | NA | NA | Community respondents: teachers in public schools | 236 | Cross-sectional |
| 9 | Insany^84^ | 2015 | 2014 | Bandung, West Java | Java | Primary | Public | Outpatients from primary health centres and pharmacies | 508 | Cross-sectional |
| 10 | Kondoj^85^ | 2020 | 2019-2020 | Manado, North Sulawesi | Sulawesi | NA | NA | Community respondents | 290 | Cross-sectional |
| 11 | Kristina^86^ | 2020 | 2018 | Yogyakarta, DIY | Java | Primary | NA | Outpatients at clinics and pharmacies | 268 | Cross-sectional |
| 12 | Kurniawan^87^ | 2017 | 2015 | Manado, North Sulawesi | Sulawesi | Primary | Public | Community respondents at primary health centres | 400 | Cross-sectional |
| 13 | Novelni^88^ | 2020 | 2019 | Padang, West Sumatra | Sumatra | NA | NA | Community respondents | 100 | Cross-sectional |
| 14 | Nuraini^89^ | 2018 | 2017-2018 | Bangkalan, East Java | Java | Secondary | Public | Outpatients at secondary hospital | 103 | Cross-sectional |
| 15 | Pratama^90^ | 2018 | 2016 | Jember, East Java | Java | NA | NA | Community respondents: non-medical university students | 324 | Cross-sectional |
| 16 | Salsabila^91^ | 2020 | 2019 | Yogyakarta, DIY | Java | NA | NA | Community respondents in rural and urban areas | 125 | Cross-sectional |
| 17 | Siahaan^92^ | 2017 | 2015 | No city mentioned, West Java, DKI Jakarta, Southeast Sulawesi | Java, Sulawesi | NA | NA | Community respondents: household members | 1271 | Cross-sectional |
| 18 | Siswati^93^ | 2009 | 2000 | Padang, West Sumatra | Sumatra | Primary | Public | Healthcare providers | 110 | Cross-sectional |
| 19 | Tandjung^94^ | 2021 | 2020-2021 | Manado, North Sulawesi | Sulawesi | NA | NA | Community respondents | 323 | Cross-sectional |
| 20 | WHO^95^ | 2015 | 2015 | National | National | NA | NA | Community respondents: household members | 1027 | Cross-sectional |
| 21 | Widayati^96^ | 2011 | 2010 | Yogyakarta, DIY | Java | NA | NA | Community respondents: household members | 559 | Cross-sectional |
| 22 | Widayati^97^ | 2012 | 2010 | Yogyakarta, DIY | Java | NA | NA | Community respondents: household members | 559 | Cross-sectional |
| 23 | Yulia^98^ | 2019 | 2019 | Bukittinggi, West Sumatra | Sumatra | Primary | Public | Community respondents | 100 | Cross-sectional |
| 24 | Yuliani^99^ | 2014 | 2014 | Kupang, East Nusa Tenggara | Nusa Tenggara | NA | NA | Community respondents | 100 | Cross-sectional |
| 25 | Zhang^100^ | 2020 | 2018 | National | National | NA | NA | Healthcare providers: General practitioners and paediatricians | 100 | Cross-sectional |

Abbreviations: NA, Not Applicable/available; DIY, Daerah Istimewa Yogyakarta; DKI, Daerah Khusus Ibukota

Table S4. Summary of studies on antibiotic consumption in inpatients and outpatients

Table S4a. Summary of studies on antibiotic consumption based in inpatients (expressed as defined daily dose [DDD]/100 bed-days)

| First author | Year of publication | Health care level | Location | Study population | No. of patients | Total antibiotics | Access Antibiotics | | | | Watch Antibiotics | | | | | | | | | | Not recommended |
| --- | --- | --- | --- | --- | --- | --- | --- | --- | --- | --- | --- | --- | --- | --- | --- | --- | --- | --- | --- | --- | --- |
|  |  |  |  |  |  |  | Amoxicillin | Ampicillin | Cefadroxil | Metronidazole | Cefepime | Cefixime | Cefoperazone | Cefotaxime | Ceftazidime | Ceftriaxone | Ciprofloxacin | Levofloxacin | Meropenem | Moxifloxacin | Cefoperazone-Sulbactam |
| Dirga^2^ | 2021 | Secondary | Lampung | Internal medicine inpatients | 164 | 116.3 | - | - | - | 22.4 | - | 1.8 | 1.5 | 4.0 | 3.2 | 62.3 | 1.5 | 11.7 | 0.4 | - | - |
| Hadi^3^ | 2008 | Tertiary | Surabaya | Fever inpatients (pre-intervention) | 212 | 99.8 | - | - | - | - | - | - | - | - | - | - | - | - | - | - | - |
| Herawati^4^ | 2019 | Tertiary | Surabaya | Surgery inpatients | 343 | 30.4 | - | - | - | - | - | - | - | - | - | - | - | - | - | - | - |
| Kartika^5^ | 2019 | Secondary | Semarang | Internal medicine inpatients (pre-intervention) | 50 | 103.7 | 0.0 | - | - | 0.0 | - | 1.9 | - | 3.8 | - | 45.9 | 20.8 | 27.4 | - | - | - |
| Kusuma^6^ | 2016 | Secondary/ Tertiary | Banyumas | Ob/Gyn inpatients | 247 | 77.7 | 22.7 | 37.3 | 7.1 | - | - | - | 2.0 | 7.2 | - | 0.4 | - | - | - | - | - |
| Mahmudah^7^ | 2016 | Tertiary | Bandung | Digestive surgery inpatients | 208 | 18 | - | - | 0.1 | 4.6 | 0.9 | 1.1 | 0.1 | 0.3 | 0.2 | 8.8 | 0.7 | 0.1 | 0.4 | - | 0.1 |
| Massey^8^ | 2021 | Secondary | Mataram | Surgery inpatients | 323 | 414.7 | 9.0 | - | 2.0 | 3.1 | - | 45.5 | 2.1 | 23.7 | 9.6 | 258.6 | 2.0 | 16.1 | - | 26.5 | - |
| Muliani^9^ | 2021 | Secondary | Surabaya | Surgery inpatients | 164 | 144.3 | 0.7 | - | - | 12.2 | 3.1 | - | - | 0.1 | 0.1 | 75.6 | - | 0.8 | 20.9 | - | 25.5 |
| Narulita^10^ | 2020 | Secondary | Pamekasan | Surgery inpatients (pre-intervention) | 200 | 197.4 | - | - | - | 12.7 | - | - | 0.2 | 0.1 | - | 160.2 | 17.3 | 0.1 | 1.1 | 1.7 | - |
| Pratama^13^ | 2019 | Secondary | Surabaya | Surgery inpatients | 463 | 102.9 | - | - | - | 27.3 | - | - | - | 2.3 | 1.8 | 53.9 | - | 1.1 | 3.3 | - | - |
| Putri^14^ | 2021 | Secondary | Yogyakarta | Inpatients with pneumonia | 251 | 43.8 | - | - | - | - | 0.7 | 2.8 | - | 0.1 | 13.6 | 19.5 | 0.3 | 4.0 | 0.3 | 0.2 | - |
| Rachmawati^15^ | 2020 | Secondary | Pasuruan | Internal medicine inpatients | 973 | 75.3 | 0.3 | 0.1 | 4.2 | 11.7 | - | 5.3 | - | 2.3 | 1.1 | 27.8 | 8.4 | 3.6 | 2.6 | - | - |
| Sholih^16^ | 2019 | Primary | Karawang | Inpatients | 81 | 144.5 | 65.8 | - | - | - | - | - | 45.9 | - | - | 2.7 | 12.3 | - | - | - | - |
| Susanto^17^ | 2019 | Secondary | Pekanbaru | Inpatients (pre-intervention) | 5319 | 49.5 | 0.8 | - | - | - | 1.5 | - | 1.0 | 0.8 | 2.9 | 14.8 | 0.6 | 13.6 | 3.4 | 0.9 | 0.4 |
| Wikantiananda^19^ | 2019 | Tertiary | West Java province | ICU inpatients | 57 | 296.8 | - | - | - | 17.0 | 2.9 | - | - | 3.0 | 19.7 | 30.6 | 3.4 | 143.2 | 49.9 | - | - |
| Yulia^20^ | 2017 | Secondary | Surabaya | Hospital-wide inpatients | 695 | 241.3 | 20.6 | - | 2.0 | 10.4 | 9.8 | 38.1 | 1.4 | 2.0 | 1.5 | 79.2 | 9.0 | 34.5 | 21.9 | 2.8 | 0.3 |

Abbreviations: -, not available; ICU, intensive care unit; Ob/Gyn, Obstetrics and gynaecology

The table summarizes 16 studies on antibiotic consumption in inpatients, expressed as (or converted to) Defined Daily Dose (DDD) per 100 bed-days, overall and/or for individual antibiotics, and divided by primary versus secondary/tertiary care settings. We listed the top 15 antibiotics with the highest DDD reported in at least 2 studies, grouped according to the 2021 WHO AWaRe classification. We noted some discrepancies between the 2021 WHO AWaRe classification and the 2021 Indonesian Ministry of Health AWaRe classification (Kementerian Kesehatan Republik Indonesia 2021); ciprofloxacin was classified as Watch vs Access; cefoperazone-sulbactam as Not recommended vs Watch; and cefepime and meropenem as Watch vs Reserve, respectively. The ATC/DDD versions used in the studies were 2003 (Hadi 2008), 2011 (Dirga 2021, Sholih 2019), 2013 (Kusuma 2016, Trisia 2020), 2017 (Herawati 2019, Narulita 2020), 2018 (Rachmawati 2020), or not reported (Kartika 2019, Mahmudah 2016, Massey 2021, Muliani 2021, Putri 2021, Susanto 2019, Wikantiananda 2019, Yulia 2017).

Table S4b. Summary of studies on antibiotic consumption based in outpatients (expressed as defined daily dose [DDD]/1000 inhabitants per day)

| First author | Year of publication | Health care level | Location | Study population | No. of patients | Total antibiotics | Access Antibiotics | | | Watch Antibiotics | |
| --- | --- | --- | --- | --- | --- | --- | --- | --- | --- | --- | --- |
|  |  |  |  |  |  |  | Amoxicillin | Cefadroxil | Chloramphenicol | Ciprofloxacin | Erythromycin |
| Andriani^1^ | 2020 | Primary | Jambi | Outpatients | 462 | 69.3 | 40.4 | 6.4 | 0.2 | 18.5 | 3.8 |
| Perdaka^11^ | 2020 | Primary | Jambi | Outpatients | 1,255 | 63.5 | 45.7 | 1.1 | 1.2 | 14.7 | 0.8 |
| Pradipta^12^ | 2015 | Primary | Bandung | Outpatients | 5,178,106 | 290.5 | 167.6 | - | 4.2 | 16.1 | - |
| Trisia^18^ | 2020 | Primary | Jambi | Outpatients | 4,053 | 61.1 | 38.9 | 5.5 | - | 11.1 | 5.6 |

Abbreviations: -, not available.

The table summarizes 4 studies on antibiotic consumption in outpatients, expressed as (or converted to) Defined Daily Dose (DDD) per /1000 inhabitants per day, overall and/or for individual antibiotics, and divided by primary versus secondary/tertiary care settings. We listed all antibiotics with the highest DDD reported in at least 2 studies, grouped according to the 2021 WHO AWaRe classification. We noted one discrepancy between the 2021 WHO AWaRe classification and the 2021 Indonesian Ministry of Health AWaRe classification (Kementerian Kesehatan Republik Indonesia 2021); erythromycin was classified as Watch vs Access, respectively. The ATC/DDD versions used in the studies were 2013 (Trisia 2020), 2018 (Perdaka 2020), 2019 (Andriani 2020), or not reported (Pratama 2019).

Figure S1. Forest plot of 16 reports on the defined daily dose (DDD) of total antibiotic use in inpatients


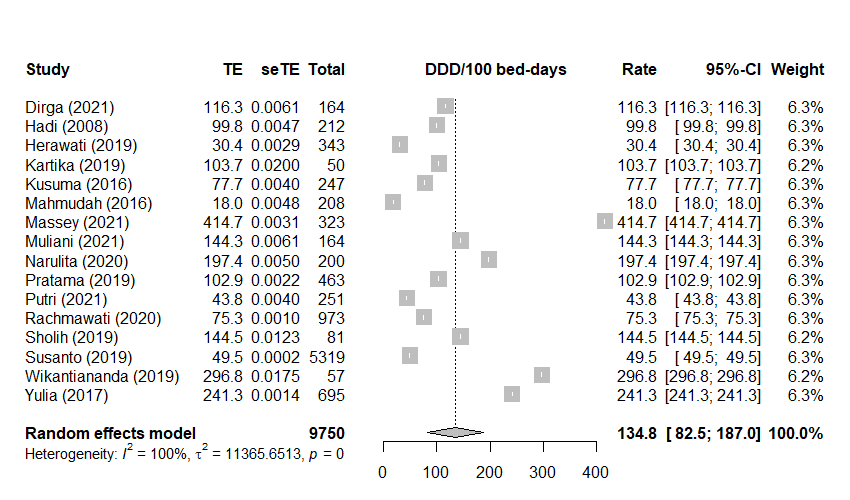


Figure S2. Forest plot of 4 reports on the defined daily dose (DDD) of total antibiotic use in outpatients


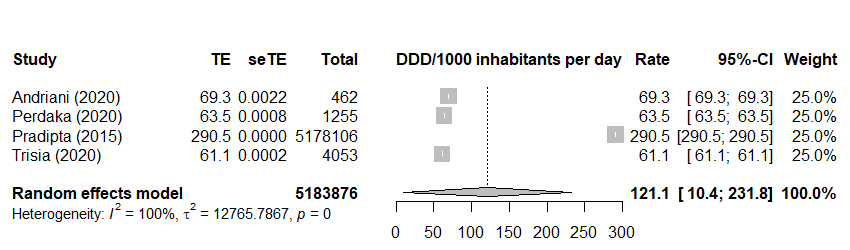


Figure S3. Results of leave-one-out sensitivity analysis for meta-analysis of the defined daily dose (DDD) of total antibiotic use in inpatients.


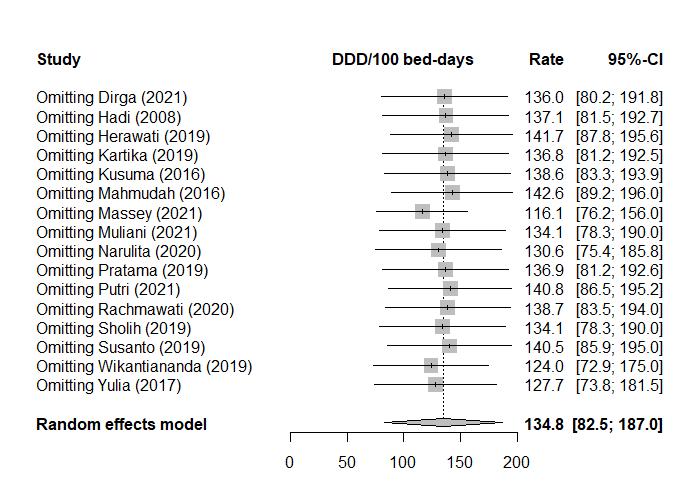


Figure S4. Results of leave-one-out sensitivity analysis for meta-analysis of the defined daily dose (DDD) of total antibiotic use in outpatients.


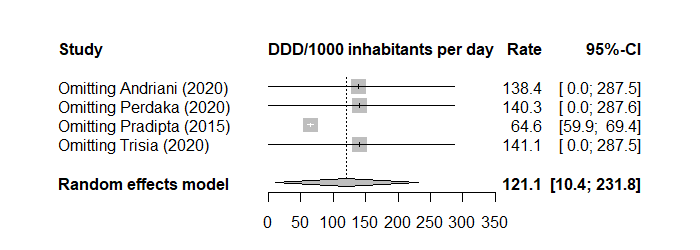


Figure S5. Funnel plot indicating evidence of publication bias (as shown by asymmetry) for meta-analysis of the defined daily dose (DDD) of total antibiotic use in inpatients


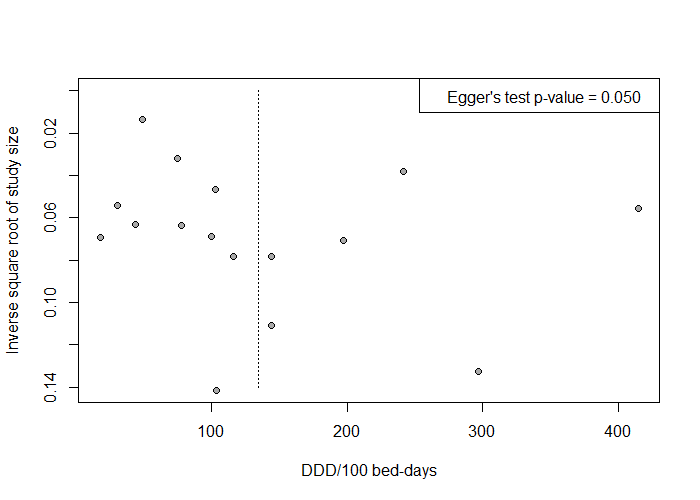


Table S5. Summary of studies on appropriateness of antibiotic prescribing (according to Gyssens method)

| First author | Year of study | Location | Healthcare level | Study population | No. of prescriptions | Classification | | | | | | | | | | | | |
| --- | --- | --- | --- | --- | --- | --- | --- | --- | --- | --- | --- | --- | --- | --- | --- | --- | --- | --- |
|  |  |  |  |  |  | iv | v | iv | | | | iii | | ii | | | i | Appropriate  (0) |
|  |  |  |  |  |  |  |  | iva | ivb | ivc | ivd | iiia | iiib | iia | iib | iic |  |  |
| Secondary/ tertiary healthcare | | |  |  |  |  |  |  |  |  |  |  |  |  |  |  |  |  |
| Aljufri^22^ | 2017-2019 | Semarang | Tertiary | Pneumonia inpatients | 98 | 0.0 | 0.0 | 5.1 | 0.0 | - | 0.0 | 0.0 | 0.0 | 1.02 | 5.1 | 0.0 | 0.0 | 88.8 |
| Hanifah^24^ | 2017 | Semarang | Secondary | Typhoid inpatients | 98 | 0.0 | 0.0 | 25.5 | 0.0 | 15.3 | 0.0 | 0.0 | 11.2 | 22.4 | 5.1 | 0.0 | 0.0 | 20.4 |
| Hardiana^25^ | 2019 | Jakarta | Tertiary | Pneumonia inpatients | 88 | 0.0 | 0.0 | 7.9 | 0.0 | 2.3 | 0.0 | 5.7 | 10.2 | 0.0 | 0.0 | 0.0 | 0.0 | 73.9 |
| Ibrahim^26^ | 2019 | Surabaya | Tertiary | Inpatients with infection | 84 | 36.9 | 11.9 | 6.0 | 0.0 | 0.0 | 0.0 | 0.0 | 0.0 | 0.0 | 1.2 | 0.0 | 0.0 | 44.1 |
| Inez^27^ | 2018 | Tanjungpura | Secondary | Paediatric inpatients | 63 | 4.8 | 82.5 | 11.1 | 0.0 | 0.0 | 0.0 | 0.0 | 1.6 | 0.0 | 0.0 | 0.0 | 0.0 | 0.0 |
| Kartika^5^ | 2018 | Semarang | Secondary | Internal medicine inpatients (pre-intervention) | 50 | 0.0 | 46 | 12 | 2.0 | 0.0 | 2.0 | 0.0 | 2.0 | 6.0 | 0.0 | 2.0 | 0.0 | 28.0 |
| Maakh^28^ | 2018 | Atambua | Secondary | Ob/gyn inpatients | 100 | 9.0 | 7.0 | 0.0 | 0.0 | 0.0 | 0.0 | 0.0 | 0.0 | 0.0 | 0.0 | 0.0 | 0.0 | 84.0 |
| Magdalena^29^ | 2017 | Pekanbaru | Secondary | ICU inpatients | 307 | 0.0 | 2.6 | 6.5 | 0.0 | 0.0 | 4.9 | 10.1 | 1.6 | 3.9 | 2.6 | 0.0 | 0.0 | 67.8 |
|  |  |  |  | Medical ward inpatients | 570 | 0.0 | 5.3 | 9.1 | 0.0 | 0.0 | 5.4 | 4.2 | 1.8 | 3.7 | 1.8 | 1.4 | 0.0 | 67.4 |
| Muthoharoh^30^ | 2017 | Pekalongan | Secondary | Surgical inpatients | 100 | 0.0 | 13.0 | 73.0 | 0.0 | 1.0 | 0.0 | 3.0 | 0.0 | 0.0 | 0.0 | 0.0 | 0.0 | 10.0 |
| Purwaningsih^31^ | 2014-2015 | Semarang | Secondary | Paediatric inpatients | 385 | - | 8.6 | 22.1 | 0.0 | 20.0 | 1.6 | 0.0 | 0.0 | 44.4 | 37.7 | 0.0 | 0.0 | 23.9 |
| Rosdiana^32^ | 2016 | Pekanbaru | Secondary | Inpatients (pre-intervention) | 92 | 0.0 | 27.2 | 35.9 | 0.0 | 2.2 | 0.0 | 0.0 | 0.0 | 1.1 | 0.0 | 0.0 | 0.0 | 33.7 |
| Setiawan^33^ | 2016 | Surabaya | Tertiary | ICU inpatients | 110 | 31.8 | 7.3 | - | - | - | - | - | - | - | - | - | - | 52.7 |
| Sumiwi^34^ | 2013 | Bandung | Secondary | Digestive surgery inpatients | 344 | - | 35.2 | 57.8 | 0.0 | 0.0 | 0.0 | 2.0 | 0.30 | 1.5 | 0.3 | 0.0 | 0.0 | 2.9 |
| Sutrisno^35^ | 2011-2012 | Yogyakarta | Tertiary | Pneumonia inpatients | 57 | 0.0 | 2.0 | 5.9 | 0.0 | 1.0 | 0.0 | 7.9 | 6.9 | 2.0 | 6.9 | 17.8 | - | 49.5 |
| Waridiarto^36^ | 2015 | Semarang | Tertiary | Orthopaedic inpatients | 150 | 1.3 | 42 | 0.0 | 0.0 | 0.0 | 8.0 | 3.3 | 0.0 | 0.0 | 0.0 | 0.0 | - | 45.3 |
| Yoanitha^37^ | 2016 | Bandung | Tertiary | Ob/gyn inpatients | 236 | 0.0 | 40.3 | - | - | - | - | 0.0 | 0.0 | 0.0 | 0.0 | 0.0 | 0.0 | 22.0 |
| Primary healthcare | |  |  |  |  |  |  |  |  |  |  |  |  |  |  |  |  |  |
| Adani^21^ | 2014 | Semarang | Primary | Paediatric inpatients | 173 | 0.0 | 62.4 | 1.7 | 0.0 | 0.0 | 0.0 | 0.0 | 0.0 | 0.0 | 0.0 | 5.2 | - | 30.6 |
| Anggraini^23^ | 2014 | Pontianak | Primary | Typhoid inpatients | 62 | 0.0 | 4.8 | 3.2 | 0.0 | 0.0 | 0.0 | 0.0 | 16.1 | 0.0 | 0.0 | 0.0 | 6.5 | 69.4 |

Abbreviations: -, not available; ICU, intensive care unit; PICU, paediatric intensive care unit; Ob/Gyn, Obstetrics and gynaecology.

The table summarizes 18 studies on the appropriateness of antibiotic prescriptions based on the Gyssens method. Gyssens evaluates the quality of each antibiotic prescription by sequentially categorizing 6 indicators from vi to i: vi=insufficient data; v=antibiotic is not indicated; iv=alternative antibiotic is available that is more effective (iva), less toxic (ivb), less costly (ivc); has narrower spectrum (ivd); iii=inappropriate duration: too long (iiia) or too short (iiib); ii=incorrect: dose (iia), interval (iib), route (iic); i=incorrect timing.

Table S6. Summary of studies on appropriateness of antibiotic prescribing (according to reference guidelines)

| First author | Year of study | Location | Health care level | Study population | No. of prescriptions | | Appropriate (%) | | | | | | | | | | | | | | | | Reference guidelines | |  |  |
| --- | --- | --- | --- | --- | --- | --- | --- | --- | --- | --- | --- | --- | --- | --- | --- | --- | --- | --- | --- | --- | --- | --- | --- | --- | --- | --- |
|  |  |  |  |  |  |  | No contra-indication/ allergy label | | Indication | | Drug choice | | Dose | | Dosing frequency | | Duration | | Administration route | | Overall appropriate use | |  |  |  |  |
| Secondary/tertiary health care | | | | | |  | |  | |  | |  | |  | |  | |  | |  | |  | |  | |  |
| Anggraini^39^ | 2020 | Jakarta | Secondary | Pre-surgical inpatients | 837 | | - | | - | | 0.5 | | 0.5 | | - | | - | | 0.5 | | 0.0 | | IN | |  |  |
| Bakhtiar^40^ | 2018 | Sorong | Secondary | Post-surgical inpatients | 83 | | 94 | | 100 | | 100 | | 100 | | - | | 92.8 | | 100 | | 79.5^a^ | | IN | |  |  |
| Bestari^42^ | 2016 | Central Java | Tertiary | Paediatric pneumonia inpatients | 90 | | 100 | | 100 | | 72.2 | | 9.2 | | - | | - | | - | | 8.9 | | NH | |  |  |
| Dania^43^ | 2014 | Yogyakarta | Secondary | Ob/gyn inpatients (therapy) | 59 | | - | | - | | - | | - | | - | | 100 | | - | | - | | IHB | |  |  |
| Dewi^44^ | 2017 | Jakarta | Tertiary | Adult ICU inpatients with sepsis or septic shock | 60 | | - | | - | | 66.7 | | 48.3 | | - | | - | | - | | - | | I | |  |  |
| Dirga^2^ | 2017 | Lampung | Secondary | Internal medicine inpatients | 168 | | 100 | | 98.8 | | 89.3 | | 52.9 | | - | | - | | - | | - | | INBH | |  |  |
| Elvina^47^ | 2016 | Jakarta | Secondary | Adult inpatients with pneumonia | 96 | | - | | - | | 86.5 | | 91.7 | | - | | 74 | | - | | - | | INB | |  |  |
| Fithria^49^ | 2013 | Semarang | Secondary | Paediatric inpatients with acute diarrhoea | 54 | | - | | - | | 0 | | - | | - | | - | | - | | - | | IB | |  |  |
| Grassella^50^ | 2018 | Pontianak | Secondary | Paediatric ARI outpatients | 340 | | 99.7 | | 91.7 | | 72.9 | | 72.6 | | 96.5 | | 56.8 | | 100 | | - | | NBW | |  |  |
| Harartasyahrani^51^ | 2020 | Prabumulih | Secondary | Pre-surgical inpatients | 119 | | - | | - | | 92.4 | | 86.4 | | 7.3 | | 40 | | 100 | | 6.7 | | IN | |  |  |
| Herlina^53^ | 2017 | Mataram | Secondary | UTI inpatients | 105 | | - | | - | | 100 | | 100 | | 99.1 | | 88.6 | | - | | - | | I | |  |  |
| Islam^54^ | 2014 | Jakarta | Tertiary | Pneumonia inpatients | 139 | | - | | - | | 5.7 | | 88.5 | | - | | 60.4 | | - | | - | | INBW | |  |  |
| Kaparang^56^ | 2013 | Manado | Tertiary | Paediatric pneumonia inpatients | 112 | | 100 | | 100 | | 100 | | 91.1 | | - | | 88.4 | | - | | - | | NHB | |  |  |
| Kurniawati^57^ | 2019 | Yogyakarta | Secondary | UTI inpatients | 61 | | - | | - | | 100 | | 88.5 | | 98.4 | | 88.5 | | 100 | | - | | IB | |  |  |
| Limato^58^ | 2019 | Jakarta | Secondary / Tertiary | Hospital-wide inpatients | 915 | | - | | - | | 52.2 | | - | | - | | - | | - | | - | | HN | |  |  |
| Nawakasari^59^ | 2017 | Klaten | Tertiary | UTI inpatients | 76 | | 100 | | 100 | | 96.1 | | 27.6 | | - | | - | | - | | - | | INBH | |  |  |
| Octavia^61^ | 2018 | Lamongan | Secondary | Ob/gyn inpatients (prophylaxis) | 54 | | - | | - | | 98.1 | | 100 | | 100 | | - | | 100 | | - | | I | |  |  |
| Ofisya^62^ | 2019 | Pontianak | Secondary | Pneumonia inpatients | 86 | | - | | 100 | | 93 | | 68.6 | | - | | 62.8 | | - | | - | | IN | |  |  |
| Oktaviani^63^ | 2014-2015 | Riau | Secondary | Ob/gyn inpatients (prophylaxis) | 140 | | - | | - | | 34.3 | | 65.7 | | 100 | | 72.9 | | 100 | | - | | INB | |  |  |
| Rusdiana^65^ | 2014 | Tangerang | Secondary | Ob/gyn inpatients (prophylaxis) | 256 | | - | | - | | 0.0 | | 7.8 | | 0 | | - | | 100 | | - | | B | |  |  |
| Zazuli^67^ | 2009 | Bandung | Secondary | Pre- and post-surgical inpatients | 1290 | | - | | 99.6 | | - | | 97.2 | | - | | - | | - | | - | | IB | |  |  |
| Primary health care | | | | | | | | | | | | | | | | | | | | | | | | | | |
| Andrajati^38^ | 2012 | Depok | Primary | Outpatients with various indications | 392 | | - | | - | | 87.2 | | - | | 98.2 | | 59.4 | | - | | 43.9 | | N | |  |  |
| Benua^41^ | 2018 | Poso | Primary | ARI outpatients | 126 | | - | | 100 | | - | | 80.9 | | 80.9 | | 100 | | 100 | | - | | N | |  |  |
| Dewi^45^ | 2018 | Jambi | Primary | Paediatric ARI outpatients | 70 | | 98.5 | | 100 | | 54.2 | | - | | 48.5 | | 1.4 | | - | | - | | N | |  |  |
| Dewi^46^ | 2019 | Jambi | Primary | Toddler ARI outpatients | 51 | | 100 | | 100 | | - | | 100 | | - | | 0 | | - | | - | | N | |  |  |
| Fakhrunnisa^48^ | 2018 | Tegal | Primary | ARI outpatients | 632 | | - | | - | | 78.0 | | 90.5 | | 98.2 | | 33.7 | | - | | 1.7 | | N | |  |  |
| Hasyul^52^ | 2017 | Garut | Primary | Typhoid outpatients | 705 | | - | | 96.9 | | 58.3 | | 63.3 | | - | | 49.8 | | - | | - | | N | |  |  |
| Jamiati^55^ | 2017 | Aceh | Primary | Outpatients | 86 | | - | | 54.6 | | - | | 60.5 | | 83.7 | | 26.7 | | - | | - | | N | |  |  |
| Nyoman^60^ | 2015 | Bandung | Primary | Paediatric ARI outpatients | 425 | | - | | 100 | | 96.5 | | 42.2 | | - | | 52.8 | | - | | - | | I | |  |  |
| Ovikariani^64^ | 2019 | Semarang | Primary | ARI outpatients | 79 | | 100 | | 23.0 | | 23.0 | | 82.3 | | - | | - | | - | | - | | N | |  |  |
| Sugiarti^66^ | 2014 | Malang | Primary | Paediatric ARI outpatients | 120 | | - | | 24.2 | | 100 | | 8.9 | | - | | - | | - | | - | | N | |  |  |

Abbreviations: ARI, acute respiratory infection; -, not available; UTI, urinary tract infection; ICU, intensive care unit; Ob/Gyn, obstetrics and gynaecology; I, international; N, national; H, hospital; B, book; W, website

The table summarizes 31 studies on appropriateness of antibiotic prescribing, based on antibiotic prescribing reference guidelines (as provided in the article), divided by primary and secondary/tertiary care settings. Appropriateness was defined by the following indicators: indication, drug choice, dose, frequency/interval, duration, and route of administration. An antibiotic prescription was classified as appropriate use if all indicators were scored as compliant with the reference guideline by the authors.

Figure S6. Forest plot showing results of meta-analysis on the overall appropriateness of antibiotic prescribing according to Gyssens method (18 reports)


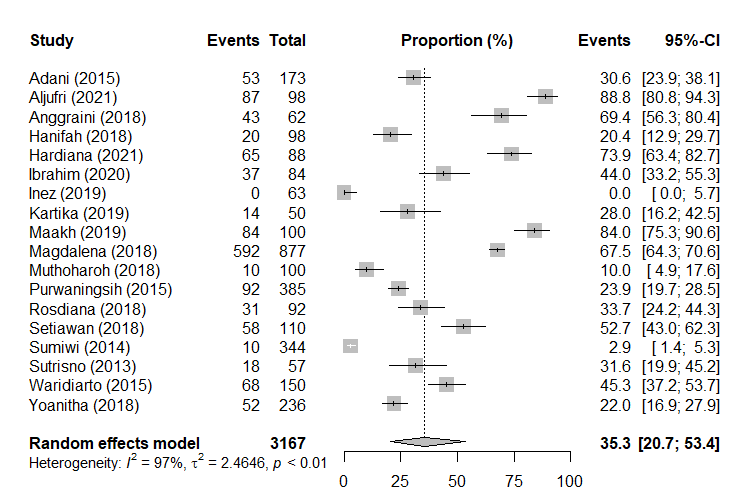


Figure S7. Results of leave-one-out sensitivity analysis for meta-analysis on the overall appropriateness of antibiotic prescribing according to Gyssens method


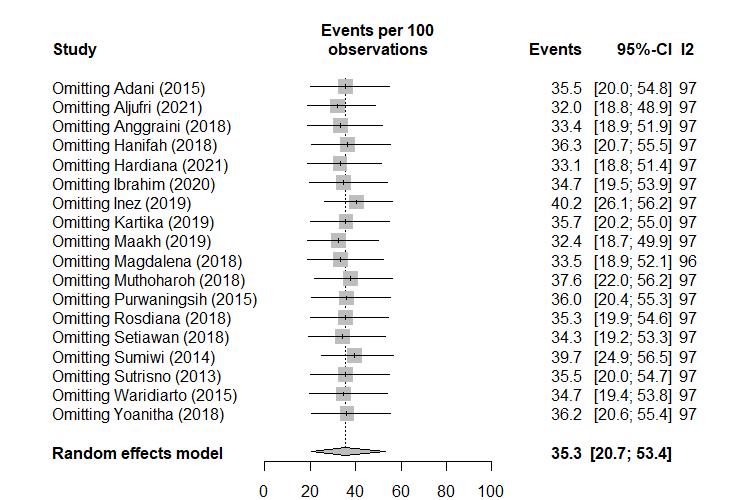


Figure S8. Results of sensitivity analysis for meta-analysis on the overall appropriateness of antibiotic prescribing according to Gyssens method by using alternative methods other than generalized linear mixed model (GLMM) method.


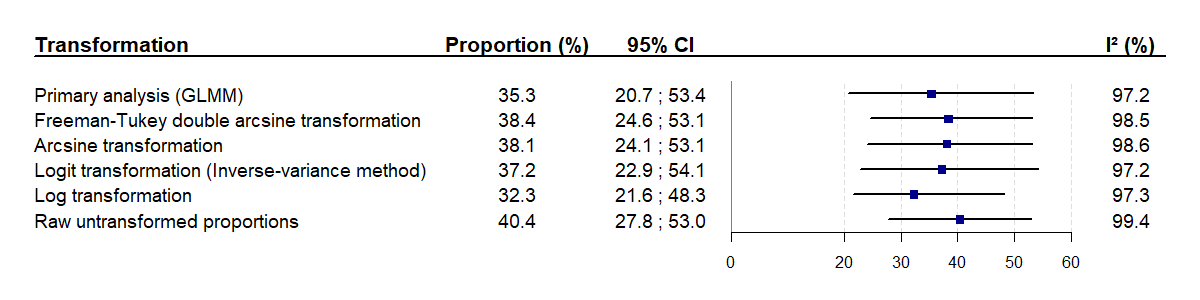


Figure S9. Funnel plot for the meta-analysis on the overall appropriateness of antibiotic prescribing according to Gyssens method. The relatively symmetrical funnel plot and the non-significant Egger’s test indicate that no publication bias is detected.


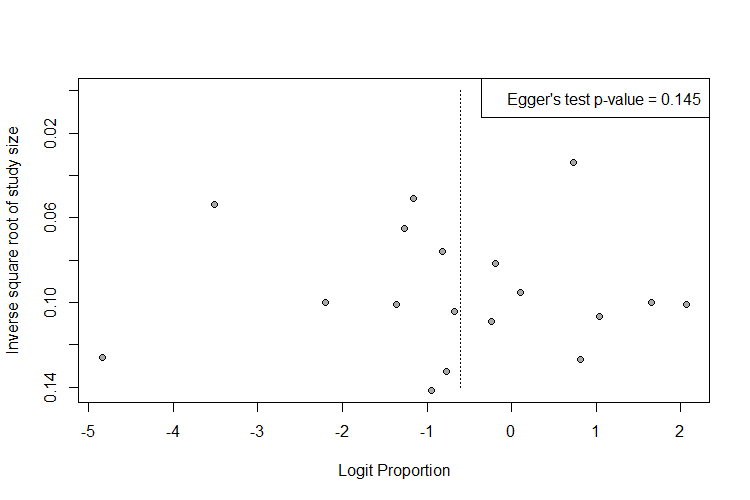


Figure S10. Summary forest plot of 19 reports on the appropriateness of antibiotic prescribing according to the “duration” indicator in the reference guidelines


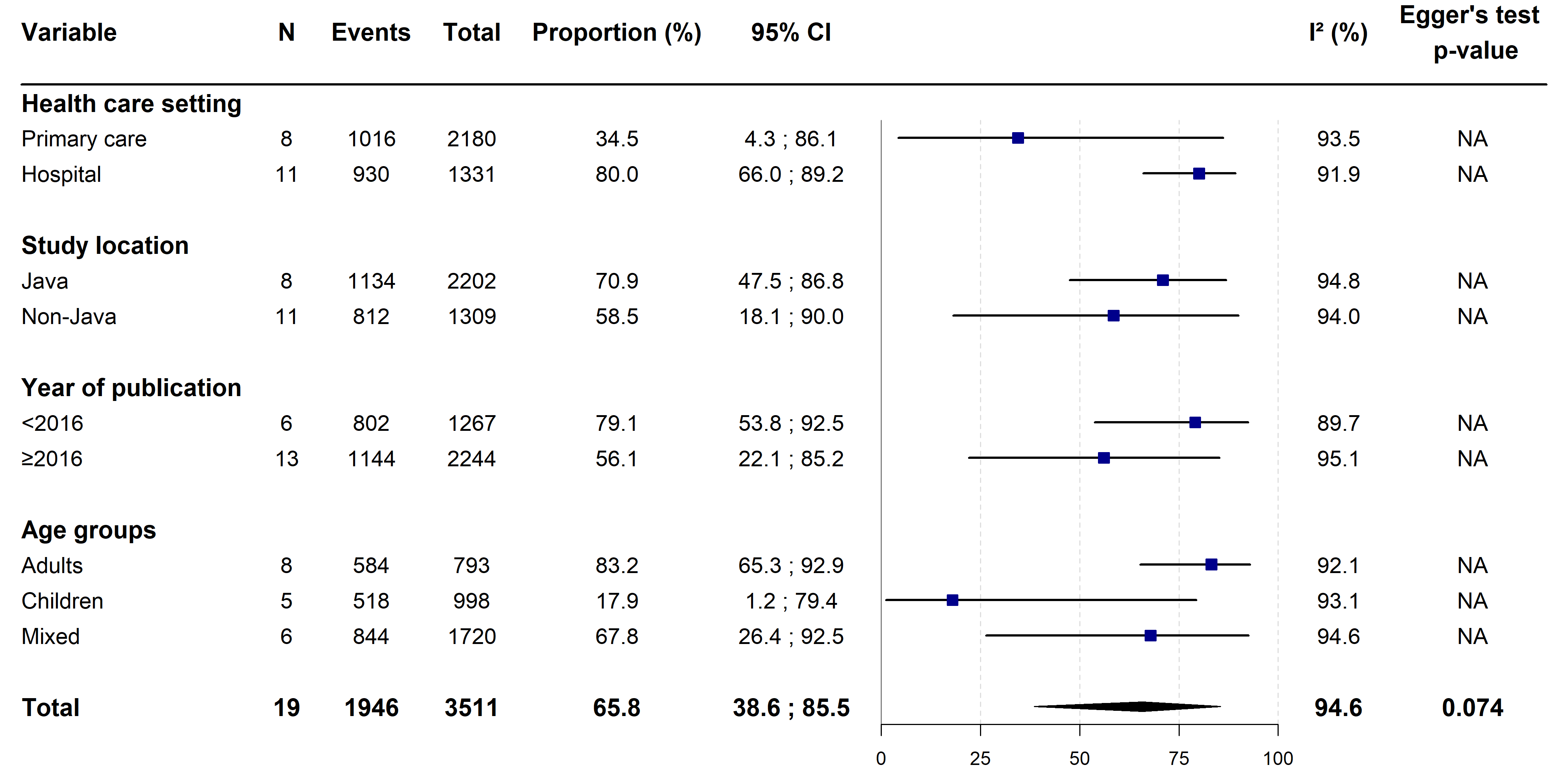


Figure S11. Summary forest plot of 27 reports on the appropriateness of antibiotic prescribing according to the “drug choice” indicator in the reference guidelines


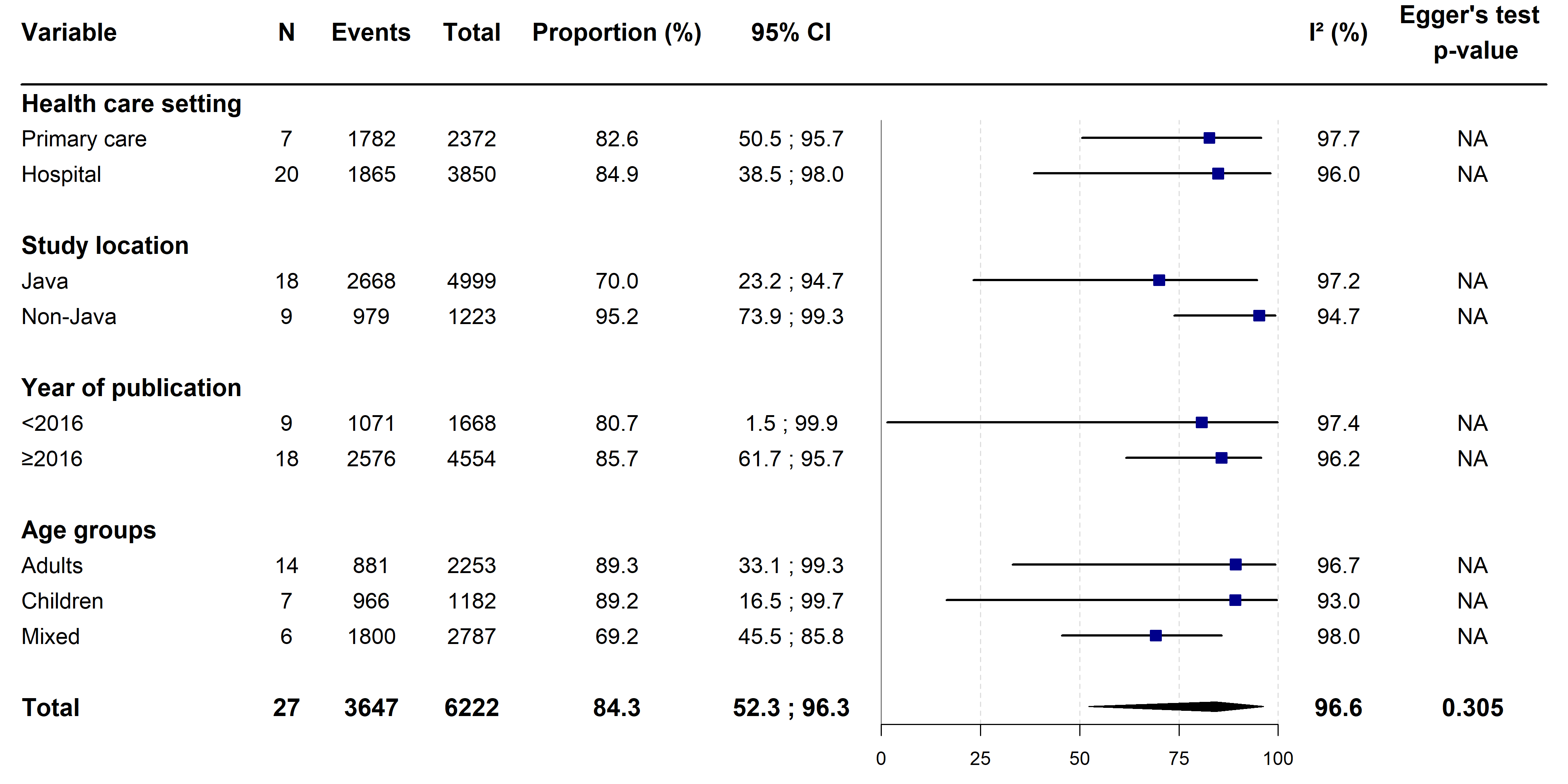


Figure S12. Summary forest plot of 27 reports on the appropriateness of antibiotic prescribing according to the “dose” indicator in the reference guidelines


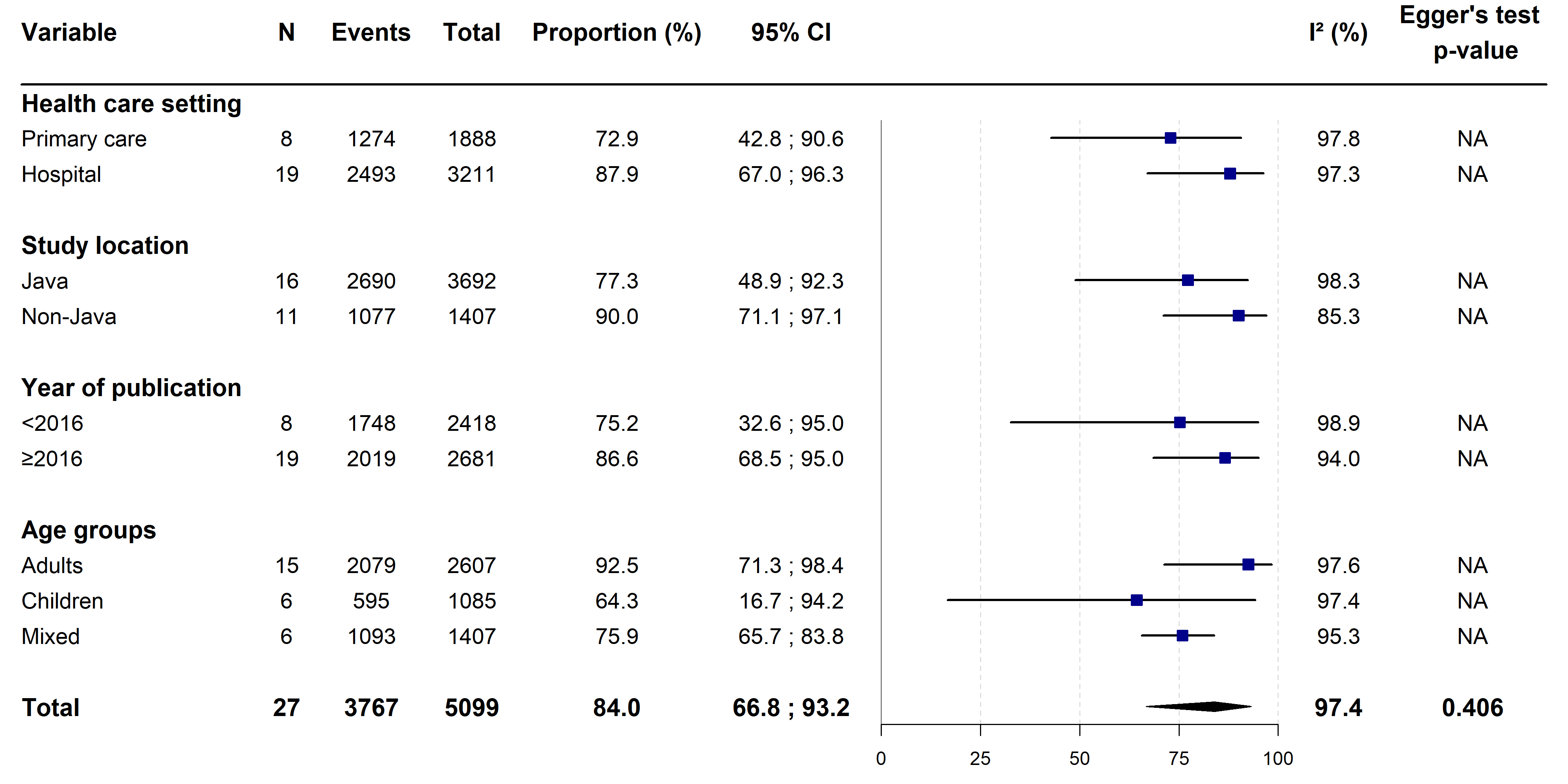


Figure S13. Summary forest plot of 6 reports on the appropriateness of antibiotic prescribing according to the “overall appropriate use” indicator in the reference guidelines


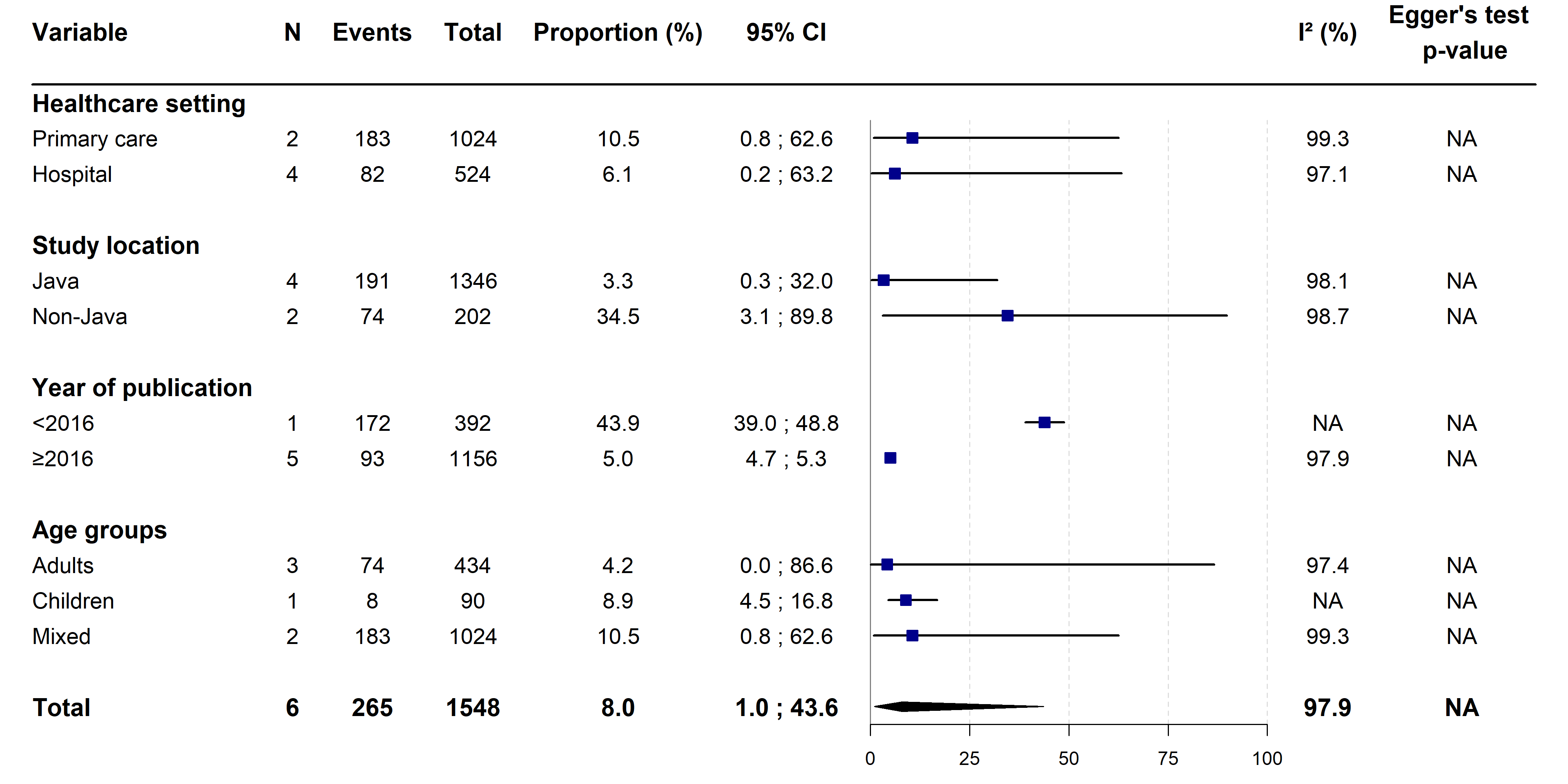


Figure S14. Summary forest plot of 9 reports on the appropriateness of antibiotic prescribing according to the “no contraindication/allergy label” indicator in the reference guidelines


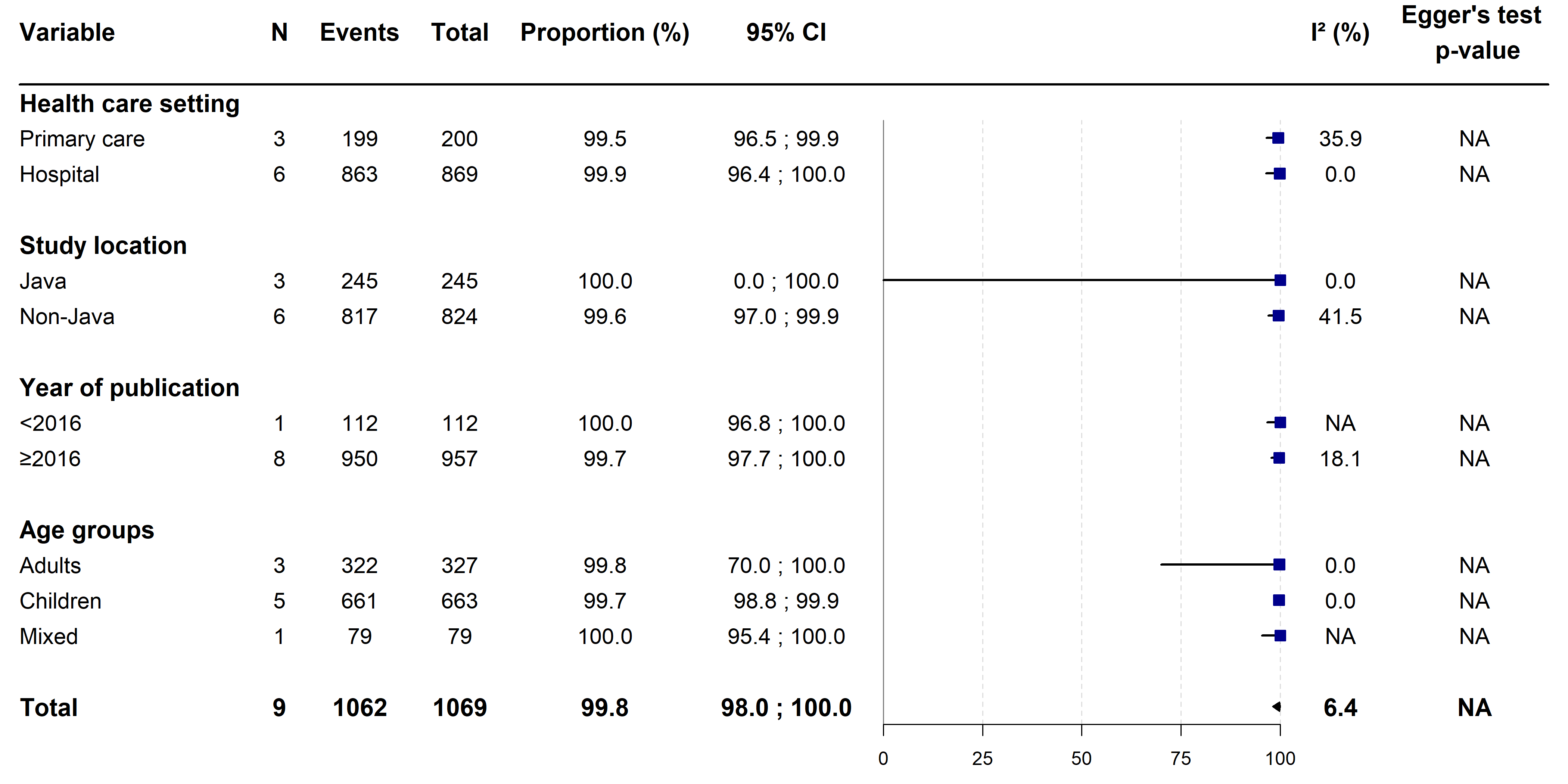


Figure S15. Summary forest plot of 16 reports on the appropriateness of antibiotic prescribing according to the “indications” indicator in the reference guidelines


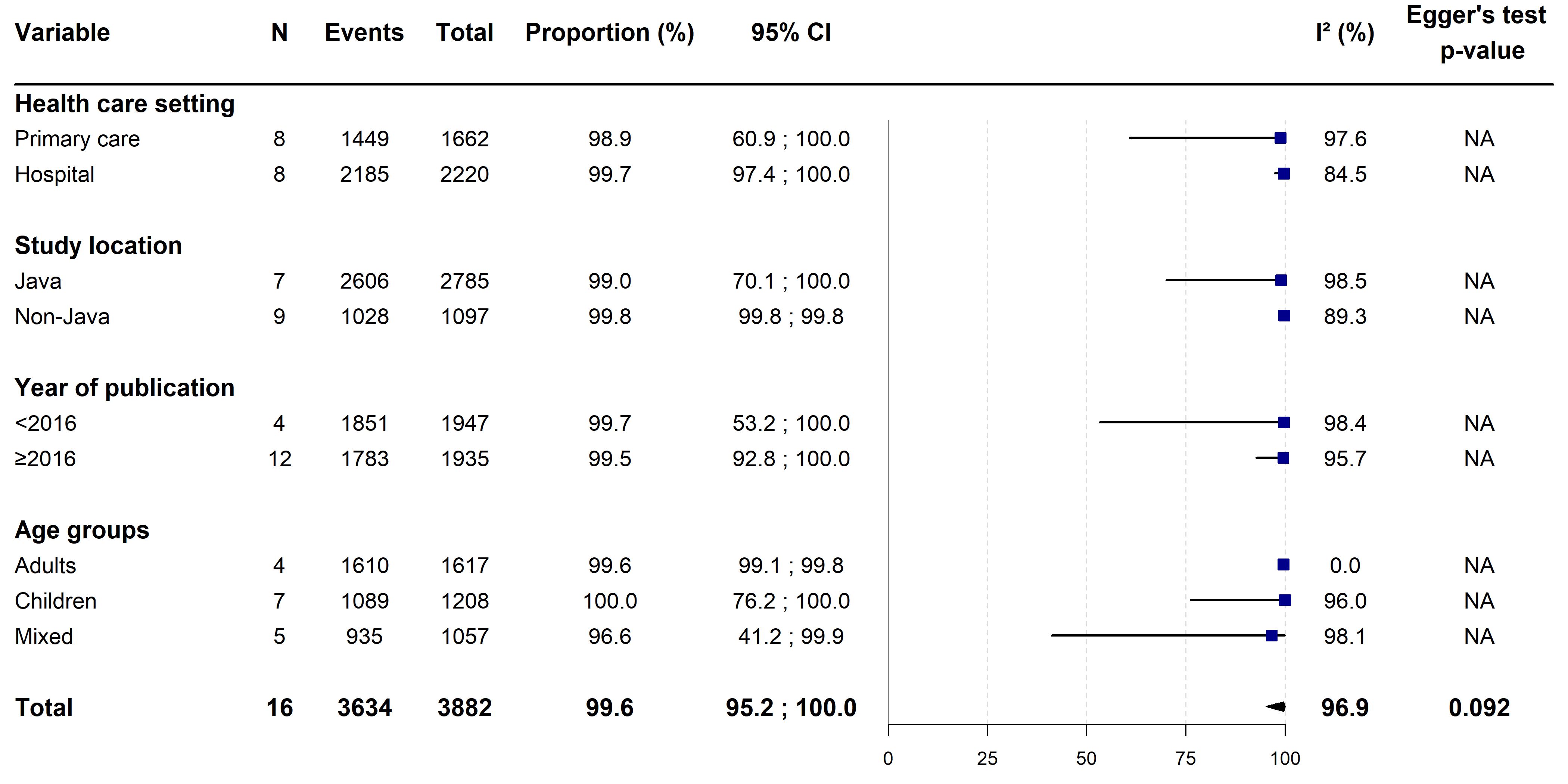


Figure S16. Summary forest plot of 13 reports on the appropriateness of antibiotic prescribing according to the “dosing frequency” indicator in the reference guidelines


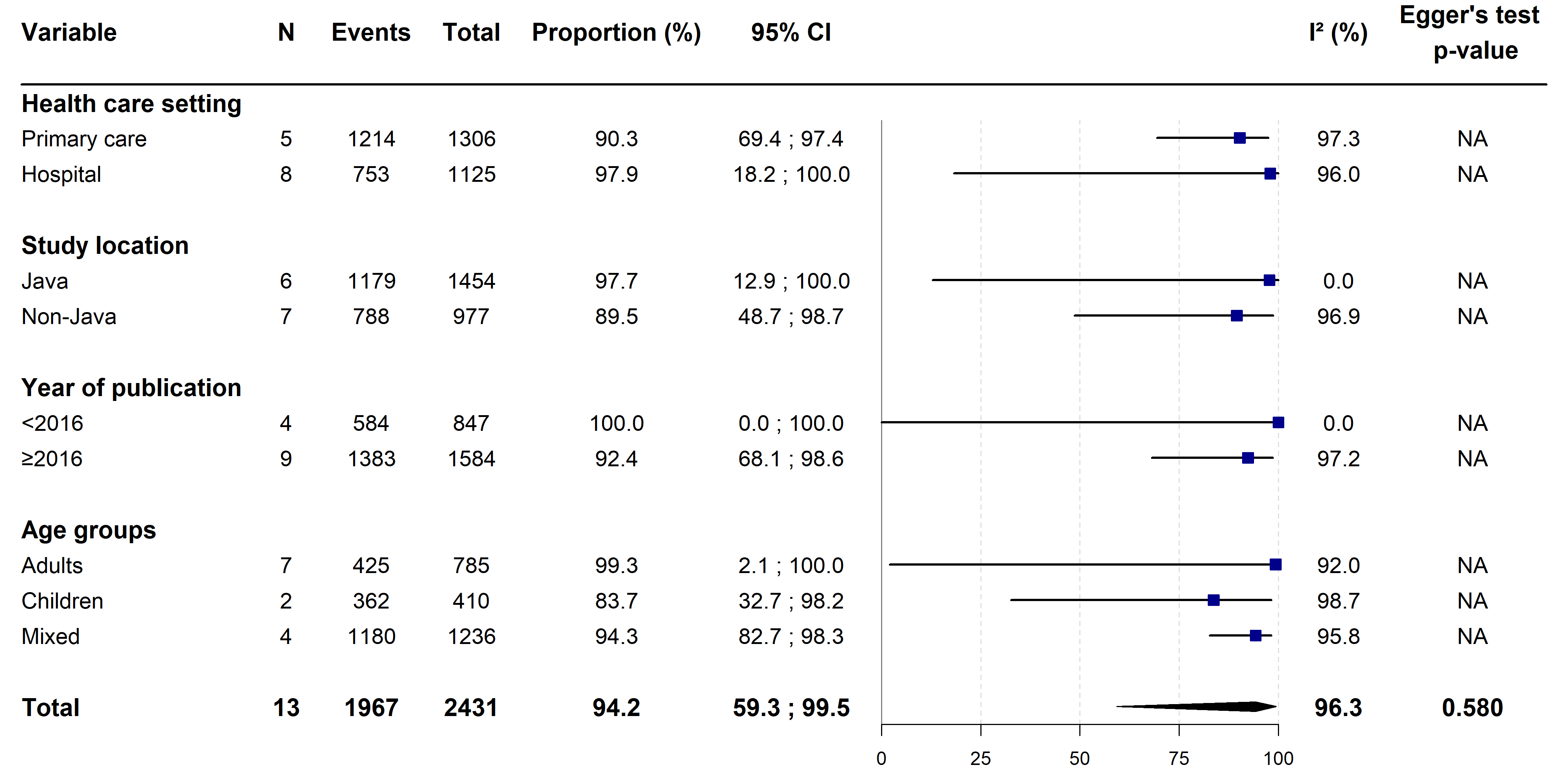


Figure S17. Summary forest plot of 9 reports on the appropriateness of antibiotic prescribing according to the “administration route” indicator in the reference guidelines


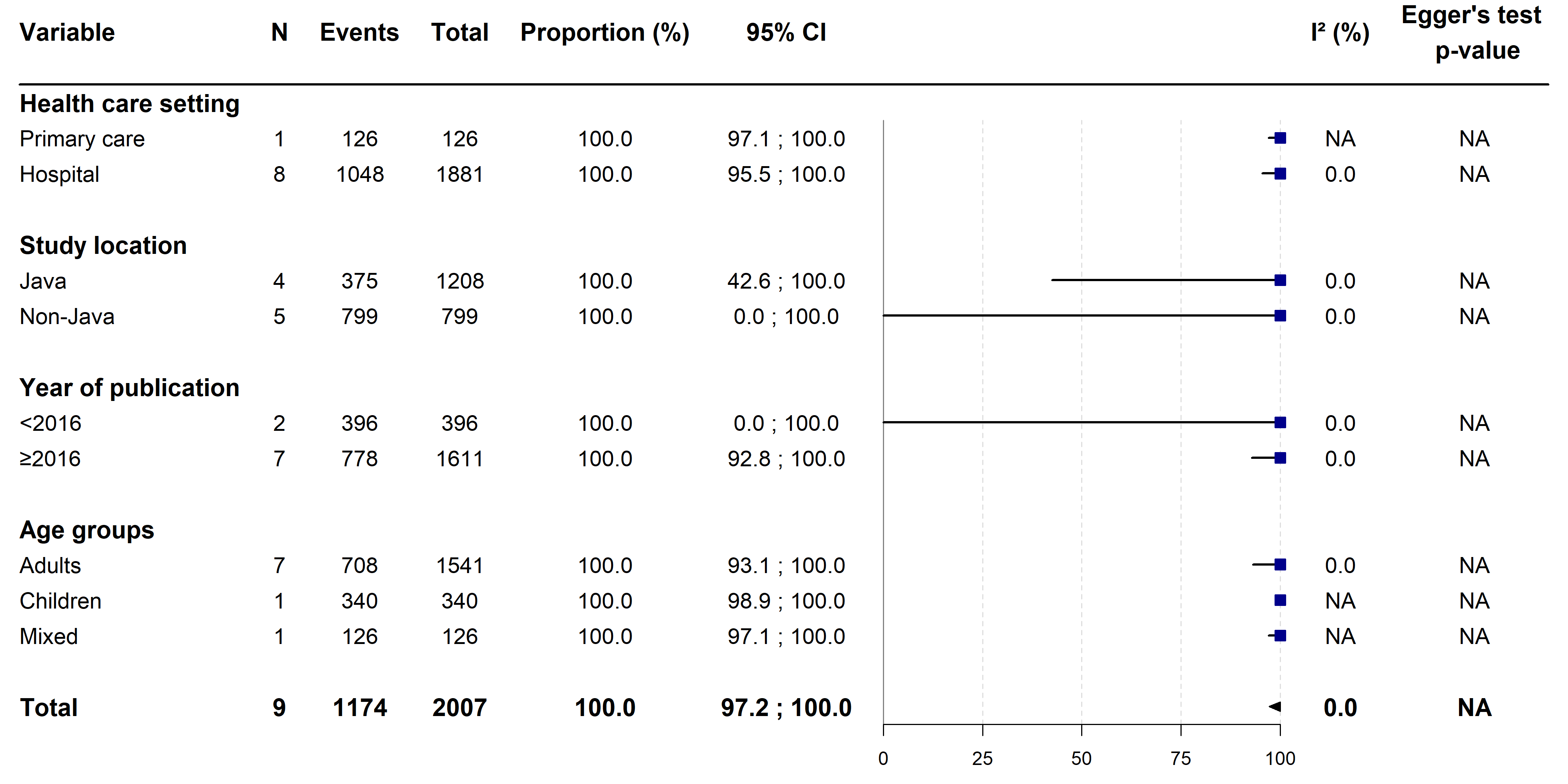


Table S7. Summary of studies on knowledge, attitudes and perceptions regarding antibiotic use

| First Author | Year of study | Location | Study population | No respondents | Questionnaire | AMR awareness | Antibiotic use knowledge | Self-medication | | Where to buy antibiotic | Advice to take antibiotic/ to practice self-medication | Reasons for self-medication | | Adherence to treatment | Antibiotic stewardship |
| --- | --- | --- | --- | --- | --- | --- | --- | --- | --- | --- | --- | --- | --- | --- | --- |
| Community | | | | | | 8 | 16 | 11 | | 9 | 10 | 6 | | 2 | 1 |
| Artini^76^ | 2014-2015 | Denpasar, Bali | Community respondents: medical and non-medical university students | 240 | Developed by the authors |  | ● | ● | | ● | ● | ● | |  |  |
| Djawaria^78^ | 2014-2015 | Surabaya, Easy Java | Community respondents at pharmacy | 267 | Existing questionnaire (Djawaria, 2018) |  |  | ● | | ● | ● |  | |  |  |
| Fatmah^79^ | 2018 | Mataram, West Nusa Tenggara | Community: medical and non-medical university students | 400 | Based on previous studies |  | ● | ● | | ● |  |  | |  |  |
| Fernandez^80^ | 2012 | Manggarai Barat, East Nusa Tenggara | Community respondents at pharmacies | 108 | Developed by the authors |  | ● | ● | | ● | ● | ● | |  |  |
| Fimanggara^81^ | 2013 | Jatinangor, West Java | Community respondents: non-medical college students | 250 | Modified from previous study | ● | ● |  | |  |  |  | |  |  |
| Fitriah^82^ | 2019 | Banjarbaru, South Kalimantan | Community respondents | 380 | Developed by the authors |  | ● |  | |  |  |  | |  |  |
| Hamid^83^ | 2019 | Pangkajene and Kepulauan, South Sulawesi | Community respondents: teachers in public schools | 236 | Developed by the authors |  |  | ● | | ● | ● | ● | |  |  |
| Insany^84^ | 2014 | Bandung, West Java | Outpatient respondents from primary health centres and pharmacies | 508 | Based on previous studies |  |  | ● | |  |  |  | |  |  |
| Kondoj^85^ | 2019-2020 | Manado, North Sulawesi | Community respondents | 290 | Developed by the authors | ● | ● | ● | |  | ● |  | |  |  |
| Kristina^86^ | 2018 | Yogyakarta, DIY | Patients at outpatient clinics and pharmacies | 268 | Modified from previous study | ● | ● |  | |  | ● |  | |  |  |
| Kurniawan^87^ | 2015 | Manado, North Sulawesi | Community respondents at primary health centre | 400 | Modified from previous studies |  | ● | ● | | ● | ● | ● | |  |  |
| Novelni^88^ | 2019 | Padang, West Sumatra | Community respondents | 100 | Developed by the authors | ● | ● |  | |  |  |  | |  |  |
| Nuraini^89^ | 2017-2018 | Bangkalan, East Java | Outpatients at secondary hospital | 103 | Developed by the authors |  | ● |  | |  |  |  | | ● |  |
| Pratama^90^ | 2016 | Jember, East Java | Community respondents: non-medical university students | 324 | Developed by the authors |  |  | ● | | ● |  |  | |  |  |
| Salsabila^91^ | 2019 | Yogyakarta, DIY | Community respondents in rural and urban areas | 125 | Based on previous studies | ● | ● |  | |  | ● |  | |  |  |
| Siahaan^92^ | 2015 | West Java, DKI Jakarta, Southeast Sulawesi | Community respondents: household members | 1271 | Developed by the authors |  |  |  | |  |  |  | | ● |  |
| Tandjung^94^ | 2020-2021 | Manado, North Sulawesi | Community respondents | 323 | Developed by the authors |  | ● | ● | |  |  | ● | |  |  |
| WHO^95^ | 2015 | National | Community respondents: household members | 1027 | Developed by specialized research agency, in collaboration with WHO | ● | ● |  | | ● | ● |  | |  | ● |
| Widayati^96^ | 2010 | Yogyakarta, DIY | Community respondents: household members | 559 | Based on previous studies in Asia |  |  | ● | | ● | ● | ● | |  |  |
| Widayati^97^ | 2010 | Yogyakarta, DIY | Community respondents: household members | 559 | Based on previous studies in Asia | ● | ● |  | |  |  |  | |  |  |
| Yulia^98^ | 2019 | Bukittinggi, West Sumatra | Community respondents | 100 | Developed by the authors | ● | ● |  | |  |  |  | |  |  |
| Yuliani^99^ | 2014 | Kupang, East Nusa Tenggara | Community respondents | 100 | Developed by the authors |  | ● |  | |  |  |  | |  |  |
| Healthcare provider | | | | | | Antibiotic dispensing (3) | | | Antibiotic use knowledge (1) | | | | The use of antibiotic guidelines (1) | | |
| Asvinigita^77^ | 2019 | Yogyakarta, DIY | Pharmacists | 250 | Developed by the authors | ● | | |  | | | |  | | |
| Siswati^93^ | 2000 | Padang, West Sumatra | Health care workers | 110 | Developed by the authors | ● | | | ● | | | | ● | | |
| Zhang^100^ | 2018 | National | General practitioners and paediatricians | 100 | Modified from existing questionnaire developed by World Gastroenterology Organization | ● | | |  | | | |  | | |

Abbreviations: AMR, antimicrobial resistance; DIY, Daerah Istimewa Yogyakarta; DKI, Daerah Khusus Ibukota; WHO, World Health Organization

The table summarizes 25 surveys in the domain attitudes and perceptions on antibiotic use, conducted among health care providers and communities, here organised by key emerging themes. Further details are provided in Table S5.

Table S8. Summary of findings of the knowledge, attitudes and practice surveys

| First author | Year of publication | Year of study | Location | Study population | No. of population | | Themes | Summary of findings |
| --- | --- | --- | --- | --- | --- | --- | --- | --- |
| Community | | | | | |  | | |
| Artini^76^ | 2016 | 2014-2015 | Denpasar, Bali | Community respondents: medical and non-medical university students | 240 | | Antibiotic use knowledge | Both medical and non-medical students were aware that antibiotic use can drive antibiotic resistance (91.7% and 84.2%) and antibiotic cannot treat all disease (90% and 92.5%). Medical students had better knowledge than non-medical student that antibiotics aim to treat bacterial infection (90.8% versus 76.7%) and not to treat viral infection (64.2% versus 49.2%). |
|  |  |  |  |  |  |  | Self-medication | Medical students were more likely to self-medicate (67.5%) than non-medical students (19.2%). |
|  |  |  |  |  |  |  | Where to buy antibiotic | >90% at the pharmacy, <10% at kiosks. |
|  |  |  |  |  |  |  | Advice to take antibiotic/ to practice self-medication | Past experience (36.5%), family (36.5%), and pharmacist (27%). |
|  |  |  |  |  |  |  | Reasons for self-medication | Self-medication was practical (77.9%) and cheaper (13.5%), doctor is far (8.6%). |
| Djawaria^78^ | 2018 | 2014-2015 | Surabaya, East Java | Community respondents buying antibiotics without a prescription at pharmacy | 267 | | Self-medication | Majority (76%) seldom self-medicate, and 12% purchased antibiotics using a previous doctor prescription. |
|  |  |  |  |  |  |  | Where to buy antibiotic | 51.3% at the pharmacy (51.3%), 9% from family and friends; others obtained antibiotics from kiosks, previous prescriptions, or unspecified. |
|  |  |  |  |  |  |  | Advice to take antibiotic/ to practice self-medication | Friends or family who work in the health sector (24%), family (21%), pharmacist (11%), self (6%), or friends who do not work in health sector (4%). |
| Fatmah^79^ | 2 019 | 2018 | Mataram, West Nusa Tenggara | Community respondents: medical and non-medical university students | 400 | | Antibiotic use knowledge | 67% of respondents used antibiotics for upper respiratory tract infection for 1-3 days as recommended by health care workers. |
|  |  |  |  |  |  |  | Self-medication | 47.5% self-medicated because it was easy to get, and many places sell antibiotics without prescriptions. Many were recommended to self-medicate by physicians or pharmacists. |
|  |  |  |  |  |  |  | Where to buy antibiotic | 88.1% respondents bought antibiotics from the pharmacy, 6.6% used the leftover antibiotics, 3.2% bought at kiosks, and 2.1% purchased at drugstores. |
| Fernandez^80^ | 2013 | 2012 | Manggarai Barat, East Nusa Tenggara | Community respondents at pharmacies | 108 | | Antibiotic use knowledge | 88% knew that antibiotics treat bacterial infection; 60-65% knew that the antibiotic course must be completed, cannot treat headache and can incur side-effects when used inappropriately. |
|  |  |  |  |  |  |  | Self-medication | All participants (100%) ever used antibiotics without a prescription. Most purchased the suggested full-course (84.2%) and kept antibiotic at home (65.7%). |
|  |  |  |  |  |  |  | Where to buy antibiotic | At the pharmacy (87%), got it from family (12%) or friends (1%). |
|  |  |  |  |  |  |  | Advice to take antibiotic/ to practice self-medication | Doctor (40%), pharmacist (28%), self (18%), friends/family (8%), or brochure (6%). |
|  |  |  |  |  |  |  | Reasons for self-medication | 44.4% reported that a doctor’s visit is too expensive, and 77% used the same antibiotic because it previously resulted in clinical improvement. |
| Fimanggara^81^ | 2016 | 2013 | Jatinangor, West Java | Community respondents: non-medical students | 250 | | AMR awareness and antibiotic use knowledge | Knowledge level was poor for 56.4%, moderate for 26.4% and good for 17.2%. Misconceptions were related to taking antibiotics for common cold, taking leftover antibiotics, and antibiotics are effective to treat the same symptoms as a previous infection and to treat inflammation. Good knowledge was shown on overuse antibiotic can cause AMR, antibiotics kill bacteria, and antibiotic should be used in according with instructions. |
| Fitriah^82^ | 2021 | 2019 | Banjarbaru | Community respondents | 380 | | Antibiotic use knowledge | ﻿58.2% respondents had a good level of knowledge about antibiotic use. 98.4% had a positive attitude towards antibiotic use. Higher education (*p*=0.007) and higher income (*p*=0.038) was correlated with better antibiotic use knowledge. |
| Hamid^83^ | 2020 | 2019 | Pangkajene and Kepulauan, South Sulawesi | Community respondents: teachers in public schools | 236 | | Self-medication | 78.4% respondents had ever used antibiotics without prescription, and 53% kept antibiotics at home for future use. |
|  |  |  |  |  |  |  | Where to buy antibiotic | 78.9% at the pharmacy, and 21.1% at kiosks. |
|  |  |  |  |  |  |  | Advice to take antibiotic/ to practice self-medication | The highest source of information to self-medicate was relatives (38.4%), followed by pharmacists (30.3%), friends (12.4%), internet (12.4%), books (2.7%), kiosk staffs (1.6%), nurses (1.1%), midwives (0.5%), and television (0.5%). |
|  |  |  |  |  |  |  | Reasons for self-medication | 74.1% stated that previous good experience was the reason to self-medicate, and the rest mentioned no influence of previous experience. |
| Insany^84^ | 2015 | 2014 | Bandung, West Java | Outpatients from 43 primary health centres and 8 pharmacies | 508 | | Self-medication | 86% respondents reported had ever practice antibiotic self-medication. Based on health belief model framework, no statistically significant was found in all 4 variables as follows: perceived benefit for not practicing self-medication (*p*=0.989): perceived barrier of no obstacles to practice antibiotic self-medication in terms of drug cost and access to healthcare (*p*=0.928), perception of the threat of antibiotic resistance *(p*=0.232), and the self-efficacy for not practicing antibiotic self-medication (*p*=0.241). |
| Kondoj^85^ | 2020 | 2019-2020 | Manado, North Sulawesi | Community respondents buying antibiotics | 290 | | AMR awareness | 43.8% respondents were aware that inappropriate use of antibiotics could lead to AMR. |
|  |  |  |  |  |  |  | Antibiotic use knowledge | 69% respondents had a low level of knowledge on antibiotic use, 14% moderate level of knowledge, and 17% had good level of knowledge. |
|  |  |  |  |  |  |  | Self-medication | Only 20% respondents stated that they always bought antibiotics with prescriptions whereas 12.7% often, 40.6% sometimes, and 26.5% never bought antibiotics with prescriptions. |
|  |  |  |  |  |  |  | Advice to take antibiotic/ to practice self-medication | 21% stated that they always self-medicated themselves according to advice from friends and relatives. |
| Kristina^86^ | 2020 | 2018 | Yogyakarta, DIY | Outpatient at clinics and pharmacies | 268 | | AMR awareness | Low awareness of AMR was shown in survey as 80.7% respondents stated that AMR is only a problem for people who take antibiotic regularly, 76.9% said that AMR occurs when the body becomes resistant to antibiotics, and 55.2% were aware that overuse antibiotic can drive AMR. |
|  |  |  |  |  |  |  | Antibiotic use knowledge | 91% respondent understood that antibiotics are aimed to treat bacterial infections, but 78% stated antibiotics can treat viral infections, and can be used to treat colds and cough (75%). |
|  |  |  |  |  |  |  | Advice to take antibiotic/ to practice self-medication | 71% respondents agreed that internet is major source of information on antibiotics, while only 58% and 45% consider pharmacists and medical professionals respectively. |
| Kurniawan^87^ | 2017 | 2015 | Manado, North Sulawesi | Community respondents at primary health centre | 400 | | Antibiotic use knowledge | Participants showed good knowledge that antibiotics kill bacteria (73%); moderate knowledge that inappropriate antibiotic use can lead to resistance (59.5%); poor knowledge related to self-medication (67.8%) and the usefulness of antibiotic i.e., for cough or runny nose (74%), does not play a role in viral infection (18%). Participants with lower level of knowledge have higher probabilities to self-medicate with antibiotics than their counterparts. |
|  |  |  |  |  |  |  | Self-medication | Of all participants, 45% self-medicated in the past six months. |
|  |  |  |  |  |  |  | Where to buy antibiotic | 52.2% at the pharmacies and 43.9% at kiosks. |
|  |  |  |  |  |  |  | Advice to take antibiotic/ to practice self-medication | Majority (70.6%) were based on own knowledge, 25,6% from family/friends. The rest from medical workers and pharmacists. |
|  |  |  |  |  |  |  | Reasons for self-medication | Doctor visit is unpractical (55.6%) or too expensive (3.3%), participants were too busy (26.7%) and used the same antibiotic previously to treat similar symptoms (14.4%). |
| Novelni^88^ | 2020 | 2019 | Padang, West Sumatra | Community respondents | 100 | | AMR awareness | 54% respondents were aware that AMR may lessen the potency of antibiotics to kill the bacteria. |
|  |  |  |  |  |  |  | Antibiotic use knowledge | 71.2% respondents have moderate-level knowledge on antibiotic use, with the lowest level of knowledge was found in low-education level group. |
| Nuraini^89^ | 2018 | 2017-2018 | Bangkalan, East Java | Outpatients at secondary hospital | 103 | | Antibiotic use knowledge | Based on health belief model framework, the perceived benefit of antibiotic use (*p*=0.021) was statistically significant, while perceived barrier to antibiotic use (*p*=0.426), perceived threat to antibiotic use (*p*=0.167), and self-efficacy of antibiotic treatment (*p*=0.872) were not statistically significant. |
|  |  |  |  |  |  |  | Adherence to treatment | Based on leftover pill counting, 55.3% completed the full antibiotic course and 44.7% did not. Participants stopped the antibiotic course because they felt better and fear of side-effects on the renal function. |
| Pratama^90^ | 2018 | 2016 | Jember, East Java | Community respondents: non-medical university students | 324 | | Self-medication | 68.5% ever used antibiotics of which 58.6% reported to purchase antibiotic without prescription. Those who have insurance were less likely to self-medicate (50%) compared to those who did not have an insurance (64%). |
|  |  |  |  |  |  |  | Where to buy antibiotic | 46.3% at the pharmacy, 12.9% at the grocery store, 12.4% from family/friends, and 10.1% at drug store. |
| Salsabila^91^ | 2020 | 2019 | Yogyakarta, DIY | Community respondents in rural and urban areas | 125 | | AMR awareness | 76.8% respondents were aware that bacteria could be resistant to antibiotics. However, 53.6% had low level of overall knowledge on antibiotic resistance. |
|  |  |  |  |  |  |  | Antibiotic use knowledge | 53.6% respondents had low level of on antibiotics use and resistance, while 46.4% had high level of knowledge. |
|  |  |  |  |  |  |  | Advice to take antibiotic/ to practice self-medication | 78.4% respondents sought healthcare professional for information on antibiotics, while 21.6% consulted internet and social media. |
| Siahaan^92^ | 2017 | 2015 | No city mentioned, West Java, DKI Jakarta, Southeast Sulawesi | Community respondents: household members | 1271 | | Adherence to treatment | 66.5% of participants took antibiotic according to doctor's instructions, and 33.9% took it only until they would feel better. |
| Tandjung^94^ | 2021 | 2020-2021 | Manado, North Sulawesi | Community respondents | 323 | | Antibiotic use knowledge | 50.5% respondents had low level of knowledge based on the response on 13 questions. 21% practiced irrational antibiotic use based on 6 indicators: information about how to use antibiotics, indication of antibiotic use, duration, dose, follow up, and dispensing. |
|  |  |  |  |  |  |  | Self-medication | 80% respondents reported ever bought antibiotics without prescriptions opposed to the rest of 20% respondents. |
|  |  |  |  |  |  |  | Reasons for self-medication | Previous use (82%), cost-effectiveness (72%), previous similar symptoms (80%). Pharmacists allowed antibiotic purchase without prescriptions (71%). |
|  |  |  |  |  |  |  | Advice to take antibiotic/ to practice self-medication | 78% respondents used unprescribed antibiotics suggested by friends or relatives. |
| WHO^95^ | 2015 | 2015 | National | Community respondents: household members | 1027 | | AMR awareness | 84% were familiar with the term AMR, 68% considered antibiotic resistance is one of the biggest world problems and 67% believed that many infections are becoming resistant to antibiotics. |
|  |  |  |  |  |  |  | Antibiotic use knowledge | 66% knew what type of disease that can be treated with antibiotic, but 63% thought cold and flu could be treated with antibiotic. 76% knew they have to take the full course of antibiotic treatment. 86% thought people should use antibiotics only when they are prescribed by doctors, but 51% thought it is ok to buy the same antibiotic if it helped previously. However, 64% disapproved of using an antibiotic given by family/friend even it was aimed to treat the same illness.  The majority took antibiotics in the past month (30%) and past six months (36%). In the last doctor's visit, 83% were prescribed with antibiotics; 87% got advice on how to take antibiotic. |
|  |  |  |  |  |  |  | Where to buy antibiotic | 96% at the pharmacy, 1% at stall or hawker, 1% from the internet, 1% saved up from previous time. |
|  |  |  |  |  |  |  | Advice to take antibiotic/ to practice self-medication | 83% from doctors/nurses, 15% did not get advice from doctors/nurses, and 1% could now remember. |
|  |  |  |  |  |  |  | Antibiotic stewardship | 88% stated that doctors should only prescribe antibiotics when needed, and 83% thought governments should promote the development of new antibiotics. However, the majority (75%) thought that lay people cannot do anything to stop AMR and believed that medical experts will solve the problem before it becomes too serious |
| Widayati^96^ | 2011 | 2010 | Yogyakarta, DIY | Community respondents: household members | 559 | | Self-medication | Of all participants who self-medicated, 80% had previous experience in using such antibiotics. A positive association was found between self-medication and having health insurance. |
|  |  |  |  |  |  |  | Where to buy antibiotic | 64% at the pharmacy, 19.5% at both pharmacy, drug store, and kiosk. |
|  |  |  |  |  |  |  | Advice to take antibiotic/ to practice self-medication | Physician or pharmacist (51.2%); friends/relatives (31.7%); multiple sources (9.8%); magazine, advertising, and brochure (4.9%); and past experiences (2.4%). |
|  |  |  |  |  |  |  | Reasons for self-medication | Reasons for self-medication were previous positive experience (54%), saving time and saving money. |
| Widayati^97^ | 2012 | 2010 | Yogyakarta, DIY | Community respondents: household members | 559 | | AMR awareness | 85% have appropriate knowledge regarding antibiotic resistance, 76% about antibiotic use for bacterial infections, but 71% had incorrect knowledge regarding antibiotics for viral infections. 50% knew that antibiotics not to be used immediately for fever. Good knowledge correlated with being male, young, higher education, and higher income. |
|  |  |  |  |  |  |  | Antibiotic use knowledge | 74% believe that antibiotics can prevent illnesses from becoming worse and 40% believe antibiotic can cure any illness. |
| Yulia^98^ | 2019 | 2019 | Bukittinggi, West Sumatra | Community respondents | 100 | | AMR awareness | 65% respondents were aware that AMR may lessen the potency of antibiotics to kill the bacteria, while 35% respondents were not. 68% did not aware that when pathogens are resistant to one antibiotic, other antibiotics might be potent to kill the pathogen. |
|  |  |  |  |  |  |  | Antibiotic use knowledge | 40% respondents stated antibiotic can cure all disease and 67% stated antibiotic can cure fungal infection. 80% were aware that full antibiotic course must be completed and 99% aware that antibiotic must be used based on doctor’s instruction. |
| Yuliani^99^ | 2014 | 2014 | Kupang, East Nusa Tenggara | Community respondents | 100 | | Antibiotic use knowledge | Important misconceptions: 94% thought antibiotics can treat every disease, 92% thought that every medicine is in the antibiotic group, 94% thought that every disease can be treated with antibiotics and 68% think that non-infectious disease, such as cold, fever, headache can be treated with antibiotics. |
| Health care providers | | | | | |  | | |
| Asvinigita^77^ | 2019 | 2019 | Yogyakarta, DIY | Healthcare providers: community pharmacists | 250 | | Antibiotic dispensing | Only 76% of pharmacists would refer the patients to physicians when symptoms are suggestive of an infection. 68% stated they would dispense antibiotic without prescriptions. Respondents had generally positive view on antimicrobial stewardship programme |
| Siswati^93^ | 2009 | 2000 | Padang, West Sumatra | Healthcare providers | 110 | | Antibiotic dispensing | 98.8% did not respond to patients’ antibiotic demand, but 12.1% gave antibiotic so the patient could leave quickly. Advice to prescribe antibiotics were obtained from community health centre doctor (33.3%), paediatricians (17.2%), and the rests were unspecified. Factors that were associated with antibiotic prescribing were level of antibiotic knowledge, attitude toward clinical guidelines, and supervision. |
|  |  |  |  |  |  |  | Antibiotic use knowledge | 54.5% participants had high knowledge and 45.5% had low knowledge. Most respondents (77.9%) never received training on rational drug use. Only 44% had other health care providers as role models in using antibiotics, while 55.6% did not. |
|  |  |  |  |  |  |  | The use of antibiotic guideline | 63.6% showed a positive attitude towards treatment guidelines, 35.4% showed a slightly positive attitude, 1% showed a slight negative attitude, and no one showed a negative attitude. |
| Zhang^100^ | 2020 | 2018 | National | Healthcare providers (General practitioners and paediatricians) | 100 | | Antibiotic dispensing | Physicians stated that around 47% medical consultation led to antibiotic dispensing, with the most common age-group to receive antibiotics was 0-1year old. Physicians in Indonesia considered side effects of antibiotics as the highest consideration that detracted them from prescribing antibiotics to children. Only 19% respondents stated they would prescribe antibiotics to a 4-yo child with a 2-day history of upper respiratory tract infection. The major determinant to prescribing antibiotics was the severity of specific clinical symptoms. |

Abbreviations: AMR, antimicrobial resistance; DIY, Daerah Istimewa Yogyakarta; DKI, Daerah Khusus Ibukota; WHO, World Health Organization

Reference list of all reports included in the systematic review

1 Andriani Y, Meirista I, Aprio Y. Evaluasi Penggunaan Antibiotik dengan Metode ATC/DDD dan DU 90% di Puskesmas Kebun Handil Kota Jambi Periode 2018 dan 2019. *JOURNAL OF HEALTHCARE TECHNOLOGY AND MEDICINE* 2020; 6: 700.

2 Dirga D, Khairunnisa SM, Akhmad AD, Setyawan IA, Pratama A. Evaluasi Penggunaan Antibiotik pada Pasien Rawat Inap di Bangsal Penyakit Dalam RSUD. Dr. H. Abdul Moeloek Provinsi Lampung. *Jurnal Kefarmasian Indonesia* 2021; : 65–75.

3 Hadi U, Keuter M, … HVA. Optimizing antibiotic usage in adults admitted with fever by a multifaceted intervention in an Indonesian governmental hospital. *Tropical Medicine & International Health* 2008; 13: 888–99.

4 Herawati F, Yulia R, Hak E, *et al.* A Retrospective Surveillance of the Antibiotics Prophylactic Use of Surgical Procedures in Private Hospitals in Indonesia. *Hospital Pharmacy* 2019; 54: 323–9.

5 Kartika N, Lestari E, Farida H, Ciptaningtyas VR. Kualitas dan Kuantitas Penggunaan Antibiotik pada Kasus Penyakit Dalam Sebelum dan Setelah Penyuluhan PPRA Di Rumah Sakit Nasional Diponegoro. *Diponegoro Medical Journal (Jurnal Kedokteran Diponegoro)* 2019; 8: 1306–18.

6 Kusuma A, Galistiani G, Wijayanti D. Evaluasi Kuantitatif Penggunaan Antibiotik pada Pasien Caesarean Section di RSUD se-Kabupaten Banyumas. *Jurnal Farmasi Indonesia* 2016; 8. http://www.jfionline.org/index.php/jurnal/article/view/245 (accessed May 8, 2019).

7 Mahmudah F, Sumiwi SA, Hartini S. Study of the Use of Antibiotics with ATC/DDD System and DU 90% in Digestive Surgery in Hospital in Bandung. *Indonesian Journal of Clinical Pharmacy* 2016; 5: 293–8.

8 Massey FK, Yulia R, Herawati F. Profil Kualitas dan Kuantitas Penggunaan Antibiotik Profilaksis pada Pre, On, dan Pos Bedah di Rumah Sakit Provinsi (RSP) NTB. *Jurnal Sains Farmasi & Klinis* 2021; 8: 43.

9 Muliani N, Herawati F, Yulia R, Wijono H. Quantity and quality profiles of antibiotics pre, on, and post surgery in a hospital setting. *International Journal of Clinical Pharmacy* 2021. DOI:10.1007/s11096-021-01251-0.

10 Narulita L, Bilal R, Akram M, Suharjono S. Analysis of Antibiotics on Patients Surgery, before and after Used Guidelines for Antibiotics (PPAB). *Jurnal Pharmascience* 2020; 7: 51.

11 Perdaka W, Sagita D, Pratama S. Studi Penggunaan Antibiotik Berdasarkan ATC / DDD Dan Du 90 % Di Puskesmas X Kota Jambi Periode 2017-2018 Study of Antibiotic Use Based on ATC / DDD and Du 90 % in Community Health Centers X City OfJambi Period 2017-2018. *Journal of Healthcare Technology and Medicine* 2020; 6: 26–32.

12 Pradipta IS, Ronasih E, Kartikawati AD, *et al.* Three years of antibacterial consumption in Indonesian Community Health Centers: The application of anatomical therapeutic chemical/defined daily doses and drug utilization 90% method to monitor antibacterial use. *Journal of Family and Community Medicine* 2015; 22: 101.

13 Pratama NYI, Suprapti B, Ardhiansyah AO, Shinta DW. Analisis Penggunaan Antibiotik pada Pasien Rawat Inap Bedah dengan Menggunakan Defined Daily Dose dan Drug Utilization 90% di Rumah Sakit Universitas Airlangga. *Indonesian Journal of Clinical Pharmacy* 2019; 8: 256.

14 Putri Hadiningrat Ayunda, Akrom A, Risdiana I. Jurnal Farmasi Sains dan Praktis ANALISIS PENGGUNAAN ANTIBIOTIK PADA PASIEN COMMUNITY-ACQUIRED PNEUMONIA (CAP) DI RS PKU MUHAMMADIYAH GAMPING ANALYSIS OF THE USE ANTIBIOTICS IN COMMUNITY-ACQUIRED PNEUMONIA (CAP) PATIENTS AT PKU MUHAMMADIYAH GAMPING HOSPIT. 2021 DOI:10.31603/PHARMACY.V7I1.3251.

15 Rachmawati S, Fazeri RL, Norcahyanti I. Gambaran Penggunaan Antibiotik di Bangsal Penyakit Dalam RSUD Bangil Kabupaten Pasuruan. *JPSCR: Journal of Pharmaceutical Science and Clinical Research* 2020; 01: 12–21.

16 Sholih MG, Sudarjat H, Saula LS. Gambaran Penggunaan Antibiotik Berdasarkan metode ATC / DDD dan DU 90 % di salah satu PUSKESMAS Karawang. *Health Science Growth* 2019; : 31–7.

17 Susanto M, Qanti PO, Anggraini D, Enda WG. Decline in Antibiotic Use after Implementation of Restriction Program at Eka Hospital, Pekanbaru, Riau. In: The 5th International Conference on Public Health. 2019: 1–7.

18 Trisia FJ, Sagita D, Pratama S, *et al.* Evaluasi Penggunaan Antibiotik Dengan Metode ATC / DDD Dan DU 90 % Di Dua Puskesmas Kota Jambi Periode 2017-2018 Evaluation Of Antibiotic Use By Method ATC / DDD And DU 90 % In Two Health Centers Jambi Period 2017-2018. *Journal of Healthcare Technology and Medicine* 2020; 6: 125–38.

19 Wikantiananda T, Tjahjadi AI, Sudjud RW. Antibiotic Utilization Pattern in the Intensive Care Unit of Tertiary Hospital in West Java, Indonesia. *International Journal of Integrated Health Sciences* 2019; 7: 81–7.

20 Yulia R, Yuaraningtyas G, Wijono H. Profil penggunaan antibiotik dan peta kuman di ruang rawat RS Husada Utama Surabaya. 2017. http://repository.ubaya.ac.id/31867/ (accessed May 7, 2019).

21 Adani NL, Arkhaesi N, Anam M. Hubungan usia anak dan diagnosis dengan rasionalitas penggunaan antibiotik pada pasien anak di Puskesmas Rowosari Semarang. *Jurnal Kedokteran Diponegoro* 2015; 4: 1277–85.

22 Aljufri AQ, Yasin NM, Wahyono D. Rasionalitas Terapi Antibiotik Empiris Pada Pasien Pneumonia di Instalasi Rawat Inap RSUP Dr. Kariadi Semarang. *Majalah Farmaseutik* 2021; 17: 89.

23 Anggraini ENF, Wicaksono A, Armyanti I. Rasionalitas Penggunaan Antibiotik pada Kasus Typhus Abdominalis di Puskesmas Siantan Hilir Kota Pontianak Tahun 2014. *Jurnal Mahasiswa PSPD FK Universitas Tanjungpura* 2018; 2. http://jurnal.untan.ac.id/index.php/jfk/article/view/23543 (accessed May 8, 2019).

24 Hanifah H, Sari I, Nuryastuti T. Evaluasi Penggunaan Antibiotik Empiris dan Analisis Biaya Demam Tifoid di Sebuah RS Swasta Kota Semarang. *jsfk.ffarmasi.unand.ac.id* 2018; 5: 1–6.

25 Hardiana I, Ratih Laksmitawati D, Utami Ramadaniati H, *et al.* EVALUASI PENGGUNAAN ANTIBIOTIKA PADA PASIEN PNEUMONIA KOMUNITAS DI INSTALASI RAWAT INAP RSPAD GATOT SUBROTO. *Original Article MFF* 2021; 25: 1–6.

26 Ibrahim AM, Widyati W, Prasetyadi FOH. Analisis Kualitatif Penggunaan Antibiotik pada Pasien Rujukan dengan Metode Analisis Alur Gyssen di RSPAL Dr. Ramelan Surabaya. *MPI (Media Pharmaceutica Indonesiana)* 2020; 3: 88.

27 Inez A, Susanti Program Studi Farmasi R, Kedokteran Universtas Tanjungpura Jl Profesor Dokter Hadari Nawawi FH, *et al.* Evaluasi Rasionalitas Penggunaan Antibiotik pada Pasien Anak Rawat Inap di Rumah Sakit Universitas Tanjungpura Periode Januari-Juni 2018 Evaluation of Antibiotic Rationality for Pediatric Inpatient at Hospital of Tanjungpura University Period. 2019 https://jurnal.untan.ac.id/index.php/jmfarmasi/article/view/37615 (accessed Feb 7, 2021).

28 Maakh DT, Da A, Fraga SS, Firdaus A. Rationality Evaluation of The Use of Prophylactic Antibiotics In Sectio Caesarea Patients In Hospitals. Mgr. Gabriel Manek, Svd Atambua in 2018. Universitas Citra Bangsa, 2019 http://www.cyber-chmk.net/ojs/index.php/farmasi/article/view/641 (accessed Feb 4, 2021).

29 Magdalena R, Bachtiar A. Antimicrobial Resistance Control Program on The Rational Use of Antibiotics In Eka Hospital Pekanbaru, Indonesia. *publications.inschool.id* 2018. http://publications.inschool.id/index.php/icash/article/view/258 (accessed May 9, 2019).

30 Muthoharoh A, Iana ND, Rahmatullah St, Wirasti W. Evaluasi kualitatif penggunaan antibiotika profilaksis di instalasi bedah sentral secara retrospektif. 2019 http://repository.urecol.org/index.php/proceeding/article/view/386 (accessed May 8, 2019).

31 Purwaningsih AEDA, Rahmawati F, Wahyono D. Evaluasi Penggunaan Antibiotik Pada Pasien Pediatri Di Bangsal Rawat Inap Rumah Sakit Islam Sultan Agung Semarang. 2015. http://etd.repository.ugm.ac.id/index.php?act=view&buku_id=83867&mod=penelitian_detail&sub=PenelitianDetail&typ=html (accessed May 9, 2019).

32 Rosdiana D, Anggraini D, Balmas M, Effendi D, Bet A. Peningkatan Rasionalitas Penggunaan Antibiotik Pasca Implementasi Kebijakan Penggunaan Antimikroba di RSUD Arifin Achmad Pekanbaru. *Jurnal Kedokteran Brawijaya* 2018; 30: 36–40.

33 Setiawan S, Widyati W, Harijono P. Profil Penggunaan Antibiotik Pascapencanangan Penerapan Program Pengendalian Resistensi Antibiotik di Intensive Care Unit Rumah Sakit TNI-AL dr. Ramelan. *Jurnal Farmasi Klinik Indonesia* 2018; 7: 30–7.

34 Sumiwi SA. Quality of Antibiotics Use in Patients with Digestive Surgery in Hospital in Bandung City. *Indonesian Journal of Clinical Pharmacy* 2014; 3: 135–40.

35 Sutrisno E, Humardewayanti R, Bayupurnama P. Impact of irrational antibiotic therapy to hospital cost of care of pneumonia in patients in Dr. Sardjito Hospital. *Acta Interna: The Journal of Internal Medicine* 2013; 3: 67–71.

36 Waridiarto DS, Agus Priambodo, Endang Sri Lestari. Kualitas penggunaan antibiotik pada kasus bedah orthopedi di bangsal bedah RSUP Dr. Kariadi. *Media Medika Muda* 2015; 4: 618–25.

37 Yoanitha N, Wirakusumah F, Sukarsa R. Gambaran Rasionalitas Penggunaan Antibiotik berdasarkan Kriteria Gyssens di Bangsal Obstetri dan Ginekologi RSUP Dr. Hasan Sadikin Bandung. *obgynia.com* 2018. http://www.obgynia.com/obgyn/index.php/obgynia/article/view/37 (accessed May 9, 2019).

38 Andrajati R, Tilaqza A, Supardi S. Factors related to rational antibiotic prescriptions in community health centers in Depok City, Indonesia. *Journal of infection and public health* 2016; 10: 41–8.

39 Anggraini AB, Syachroni. Penggunaan Antibiotik Profilaksis pada Bedah Bersih di Rumah Sakit di Jakarta. *Jurnal Penelitian dan Pengembangan Pelayanan Kesehatan* 2020; 4: 7–12.

40 Bakhtiar, Makaba S, Hasmi, Mallongi A. Rationality of Antibiotic Drug Used to Medical Patient Post-Operatively in Selebe Solu Hospital Sorong City Papua Barat Province 2018. *International Journal of Science and Healthcare Research* 2019; 4. http://ijshr.com/IJSHR_Vol.4_Issue.1_Jan2019/IJSHR0022.pdf (accessed May 9, 2019).

41 Benua GP, Tiwow GAR, Untu SD, Karauwan FA. Evaluasi Rasionalitas Penggunaan Antibiotik Pada Pasien ISPA Di Puskesmas Tonusu Kecamatan Pamona Puselemba Kabupaten Poso. *Jurnal Biofarmasetikal Tropis* 2019; 2019: 136–40.

42 Bestari MP, Karuniawati H. Evaluasi Rasionalitas dan Efektifitas Penggunaan Antibiotik pada Pasien Pneumonia Pediatrik di Instalasi Rawat Inap Rumah Sakit Pusat Jawa Tengah. *Pharmacon: Jurnal Farmasi Indonesia* 2019; 14. DOI:10.23917/pharmacon.v14i2.6524.

43 Dania H, Baroroh F, Bachri MS. Evaluasi Penggunaan Antibiotik Pada Pasien Bedah Sesar Di Rumah Sakit PKU Muhammadiyah Bantul Yogyakarta. *Pharmacy: Jurnal Farmasi Indonesia* 2016; 13. https://www.neliti.com/id/publications/161113/evaluasi-penggunaan-antibiotik-pada-pasien-bedah-sesar-di-rumah-sakit-pku-muhamm (accessed May 10, 2019).

44 Dewi RS, Radji M, Andalusia R. Evaluation of Antibiotic Use Among Sepsis Patients in an Intensive Care Unit: A cross-sectional study at a referral hospital in Indonesia. *Sultan Qaboos University Medical Journal [SQUMJ]* 2018; 18: 367.

45 Dewi R, Sutrisno D, Purnamasari R. Evaluasi Penggunaan Antibiotik pada Pasien Balita dengan Diagnosa Infeksi Saluran Pernapasan Atas di Puskesmas Koni Kota Jambi. *J Sains Kes 2020* 2020; 2: 385–90.

46 Dewi R, Sutrisno D, Medina F, *et al.* Evaluasi Penggunaan Antibiotik Infeksi Saluran Pernapasan Atas pada Anak di Puskesmas Olak Kemang Kota Jambi Tahun 2018 Evaluation of Antibiotic Use for Upper Respiratory Tract Infections in Children in Olak Kemang Public Health Center, Jambi in 2018. 2020 DOI:10.30595/PHARMACY.V17I1.6936.

47 Ridha Elvina, Nur Rahmi, Sandra Ayu Oktavira. Evaluasi penggunaan antibiotik pada pasien Community-Acquired Pneumonia (CAP) di instalasi rawat inap rumah sakit “X” Jakarta. *Pharmacy* 2017; 14: 64–74.

48 Fakhrunnisa F, Andrajadi R, Radji M. Quality of Antibiotic Prescribing for Respiratory Tract Disease in Primary Healthcare Centers in the District of Tegal, Central Java, Indonesia. *Indonesian Journal of Clinical Pharmacy* 2020; 9: 95.

49 Risha Fillah Fithria, Akroman Rohmat Di’fain. Rasionalitas terapi antibiotik pada pasien diare akut anak usia 1-4 tahun di rumah sakit Banyumanik Semarang tahun 2013. *Pharmacy* 2015; 12: 197–209.

50 Grassella, Yuswar MA, Purwanti NU. ANTIBIOTICS USAGE RATIONALITY AND DRUG INTERACTIONS IN CHILDREN’S OUTPATIENT DIAGNOSED WITH ACUTE Studi rasionalitas penggunaan antibiotik dan interaksi obat pada pasien anak terdiagnosis Infeksi Saluran Pernapasan Akut (ISPA) di instalasi rawat jalan RSU. *Jurnal Mahasiswa Farmasi Fakultas Kedokteran UNTAN* 2019; 4. https://jurnal.untan.ac.id/index.php/jmfarmasi/article/view/35316 (accessed Feb 7, 2021).

51 Harartasyahrani RA, Simamora S. Evaluasi penggunaan antibiotik profilaksis pada pasien bedah kategori highly recommended di rumah sakit “X” kota Prabumulih. *Jurnal Muara Sains, Teknologi, Kedokteran dan Ilmu Kesehatan* 2021; 5: 121.

52 Hasyul SFP, Puspitan T, Nuari DA, Muntaqin EP, Wartini E, Eka MY. Evaluasi penggunaan obat antibiotik pada pasien demam tifoid di Kabupaten Garut pada Januari-Desember 2017. *Jurnal Ilmiah Farmako Bahari* 2019; 10: 160–70.

53 Herlina D, Hasina R, Dewi NMAR. Pola peresepan antibiotik pada pasien infeksi saluran kemih di instalasi rawat jalan RSUD Provinsi NTB tahun 2017. *Sasambo Journal of Pharmacy* 2021; 2: 11–5.

54 Islam Z, Qodariyah SM, Nursehah ENE. Penggunaan Antibiotik Pada Terapi Community Acquired Pneumonia di RSUD Pasar Rebo dan RSUD Tarakan di Jakarta Tahun 2014. *Jurnal Sains dan Teknologi Farmasi* 2017; 19: 1–8.

55 Jamiati J, Abadi H, Sari M. Evaluasi Peresepan Antibiotik Pada Pasien Rawat Jalan di Puskesmas Dabun Gelang Kabupaten Gayo Lues. *Jurnal Dunia Farmasi* 2019; 3: 115–22.

56 Kaparang PC, Tjitrosantoso H, Yamlean PV. Evaluasi kerasionalan penggunaan antibiotika pada pengobatan pneumonia anak di instalasi rawat inap RSUP Prof. Dr. R. D. Kandou manado periode Januari-Desember 2013. *PHARMACON* 2014; 3. https://ejournal.unsrat.ac.id/index.php/pharmacon/article/view/5440 (accessed May 13, 2019).

57 Kurniawati H, Auliyanah A. Pattern of antibiotics use in adult patients with Urinary Tract Infection (UTI). 2021 DOI:10.20527/JBK.V17I1.10244.

58 Limato R, Nelwan EJ, Mudia M, *et al.* A multicentre point prevalence survey of patterns and quality of antibiotic prescribing in Indonesian hospitals. *JAC-Antimicrobial Resistance* 2021; 3. DOI:10.1093/jacamr/dlab047.

59 Nawakasari N, Nugraheni AY. Evaluasi Penggunaan Antibiotik pada Pasien Infeksi Saluran Kemih di Instalasi Rawat Inap RSUP X di Klaten Tahun 2017. *Pharmacon: Jurnal Farmasi Indonesia* 2019; 16: 38–48.

60 Nyoman SMH N, Sumarsono T, Wahidatun N. Study on Use of Antibiotics for the Treatment of Acute Respiratory Infections (ARIs) in Children at a Clinic in Bandung, Indonesia. 2017. https://www.rjpbcs.com/pdf/2017_8(1S)/[24].pdf (accessed May 10, 2019).

61 Octavia DR. Evaluasi Penggunaan Antibiotika Profilaksis pada Pasien Bedah Sesar. *Indonesian Journal of Health Research* 2019; 2: 23–30.

62 Ofisya M, Laviesta, Susanti R, Purwanti NU. Evaluasi penggunaan antibiotik pada pasien pneumonia rawat inap di RSUD dr. Soedarso Pontianak. 2020 https://jurnal.untan.ac.id/index.php/jmfarmasi/article/view/42256 (accessed Feb 7, 2021).

63 Oktaviani F, Wahyono D, Yuniarti E. Evaluasi penggunaan antibiotik profilaksis terhadap kejadian infeksi luka operasi pada operasi sectio caesarea. *JURNAL MANAJEMEN DAN PELAYANAN FARMASI (Journal of Management and Pharmacy Practice)* 2015. https://journal.ugm.ac.id/jmpf/article/view/29449 (accessed May 7, 2019).

64 Ovikariani S, Saptawat T, Rahma FA. Evaluasi Rasionalitas Penggunaan Antibiotik Pada Pasien Ispa Di. *Jurnal Ilmu Keperawatan dan Kebidanan STIKES Telogorejo* 2019; XI: 76–82.

65 Rusdiana N, Safitri M, Resti A. Evaluasi penggunaan antibiotika profilaksis pada pasien bedah sesar terencana di rumah sakit ibu dan anak “x” di Tangerang. *Social Clinical Pharmacy Indonesia Journal* 2016. http://journal.uta45jakarta.ac.id/index.php/SCPIJ/article/view/275 (accessed May 9, 2019).

66 Sugiarti T, Sidemen A, Wiratmo. Studi Penggunaan Antibiotik pada Pasien Penyakit ISPA Usia Bawah Lima Tahun di Instalasi Rawat Jalan Puskesmas Sumbersari Periode 1 Januari-31 Maret 2014. *jurnal.unej.ac.id* 2015. https://jurnal.unej.ac.id/index.php/JPK/article/view/2583 (accessed May 9, 2019).

67 Zazuli Z, Sukandar EY, Lisni I. Evaluasi Penggunaan Obat pada Pasien Bedah di Suatu Rumah Sakit Swasta di Bandung. *Jurnal Farmasi Klinik Indonesia* 2015; 4: 87–97.

68 Dwiprahasto I. Ketersediaan obat di kabupaten dan mutu peresepan di pusat pelayanan kesehatan primer. *Berkala Ilmu Kedokteran* 2004. http://i-lib.ugm.ac.id/jurnal/detail.php?dataId=3283 (accessed May 9, 2019).

69 Farida H, Herawati H, Hapsari M, Notoatmodjo H, Hardian H. Penggunaan antibiotik secara bijak untuk mengurangi resistensi antibiotik, Studi Intervensi di Bagian Kesehatan Anak RS Dr. Kariadi. *Sari Pediatri* 2008; 10: 34–41.

70 Hapsari M, Farida H, Keuter M, PJ B, Pediatri UH-S, 2016 U. Penurunan Penggunaan Antibiotik pada Pasien Anak dengan Demam. *Sari Pediatri* 2006; 8: 16–24.

71 Karuniawati H, Yulianti T, Aini DK, Nurwienda FI. Impact of antimicrobial stewardship program on the use of antibiotics in pneumonia patients at teaching hospital in surakarta Indonesia. *International Journal of Applied Pharmaceutics* 2021; 13: 20–3.

72 King J, Ciptaningtyas VR. Kuantitas Penggunaan Antibiotik Sebelum Dan Setelah Pembuatan Pedoman Penggunaan Antibiotik (Ppab) Penelitian Pada Kasus Bedah Digestif Rsup Dr. Kariadi Semarang. *Jurnal Kedokteran Diponegoro* 2015; 4: 1072–82.

73 Lizikri A, Arozal W, Ramadaniati HU. Effects of Clinical Pathway Implementation in Management of Pediatric Inpatients with Pneumonia: A Cross-Sectional Study in Indonesia. *International Journal of Research in Pharmaceutical Sciences* 2020; 11: 7281–8.

74 Murni IK, Duke T, Kinney S, Daley AJ, Soenarto Y. Reducing hospital-acquired infections and improving the rational use of antibiotics in a developing country: an effectiveness study. *Archives of Disease in Childhood* 2015; 100: 454–9.

75 Widowati I, Wirawan I, Nopiyani N, Sari K. Pharmacist counseling intervention to improve patient antibiotic compliance. *phpmajournal.org* 2018. https://phpmajournal.org/index.php/phpma/article/view/158 (accessed May 9, 2019).

76 Artini I, Indrayani A. Penggunaan antibiotika secara mandiri pada mahasiswa kedokteran dan non kedokteran Universitas Udayana. *ojs.unud.ac.id* 2016. https://ojs.unud.ac.id/index.php/ach/article/view/27464 (accessed May 9, 2019).

77 Asvinigita LRM, Ikapuspitasari;, Kristina SA. Antibiotics stewardship practice among community pharmacists in indonesia: A cross-sectional survey. *International Journal of Pharmaceutical Research* 2019; 11: 176–81.

78 Djawaria DAP, Setiadi AP, Setiawan E. Analisis Perilaku dan Faktor Penyebab Perilaku Penggunaan Antibiotik Tanpa Resep di Surabaya Behavior Behavior Analysis and Attributed Factors to Non Prescription Antibiotic Used in Surabaya. *JURNAL MKMI* 2018; 14. DOI:10.30597/mkmi.v14i4.5080.

79 Fatmah S, Aini SR, Pratama IS. Tingkat Pengetahuan Mahasiswa Tahun Pertama Bersama (TPB) tentang Penggunaan Antibiotik dalam Swamedikasi. *Jurnal Sains Farmasi & Klinis* 2019; 6: 200.

80 Fernandez ABM. Studi Penggunaan Antibiotik Tanpa Resep Di Kabupaten Manggarai dan Manggarai Barat – NTT. 2013; 2: 1–17.

81 Fimanggara B, Istriati, Diposarosa R. Knowledge and Attitude towards Antibiotic Use among College Students in Jatinangor. *journal.fk.unpad.ac.id* 2016. http://journal.fk.unpad.ac.id/index.php/amj/article/view/792 (accessed May 9, 2019).

82 Fitriah R, Mardiati N, Tinggi S, *et al.* Pengaruh faktor sosiodemografi terhadap pengetahuan dan sikap pada penggunaan antibiotik di kalangan masyarakat pedesaan: Studi observasional di kecamatan Cempaka Banjarbaru. 2021 DOI:10.31603/PHARMACY.V7I1.3181.

83 Hamid F, Rasmin Kotto F, Prasetia W. Karakteristik Pengguna Antibiotik tanpa Resep Dokter di Kalangan Guru Kecamatan Labakkang, Kabupaten Pangkajene dan Kepulauan. 2020 DOI:10.24252/ALAMI.V4I2.15157.

84 Insany AN, Dika P. Destiani, Anwar Sani, Lilik Sabdaningtyas, Ivan S. Pradipta. Association between Perceived Value and Self-Medication with Antibiotics: An Observational Study Based on Health Belief Model Theory. *Jurnal Farmasi Klinik Indonesia* 2015; 4: 77–86.

85 Kondoj IV, Lolo WA, Jayanto I. Pengaruh Tingkat Pengetahuan Dan Sikap Terhadap Penggunaan Antibiotik Di Apotek Kimia Farma 396 Tuminting Kota Manado. *Pharmacon* 2020; 9.

86 Kristina SA, Wati R, Prasetyo SD, Fortwengel G. Public knowledge and awareness towards antibiotics use in Yogyakarta: A cross sectional survey. *Pharmaceutical Sciences Asia* 2020. DOI:10.29090/psa.2020.02.019.0008.

87 Kurniawan K, Posangi J, Rampengan N. Association between public knowledge regarding antibiotics and self-medication with antibiotics in Teling Atas Community Health Center, East Indonesia. *Medical Journal of Indonesia* 2017; 26: 62.

88 Novelni R, Azyenela L, Septiana Y. Hubungan tingkat pendidikan masyarakat terhadap pengetahuan dalam penggunaan antibiotik oral di apotek Kecamatan Koto Tangah Padang. *Jurnal Penelitian Farmasi Indonesia* 2020; 9: 2020.

89 Nuraini A, Yulia R, Herawati F, Setiasih S. The Relation between Knowledge and Belief with Adult Patient’s Antibiotics Use Adherence. *Journal of Management and Pharmacy Practice)* 2019; 8: 165.

90 Pratama AN, Rohmawati A, Rachmawati E. A survey of antibiotics purchased without prescription among non-health science students in Jember, Indonesia. *Jurnal Farmasi Sains dan Komunitas* 2018; 15: 47–54.

91 Salsabila NN, Kristina SA. Awareness on antibiotic resistance among lay people in Yogyakarta, Indonesia. *INTERNATIONAL JOURNAL OF PHARMACEUTICAL RESEARCH* DOI:10.31838/ijpr/2020.12.03.023.

92 Siahaan S, Usia T, Pujiati S, *et al.* Pengetahuan, sikap, dan perilaku masyarakat dalam memilih obat yang aman di tiga Provinsi di Indonesia. *neliti.com* 2017. https://www.neliti.com/publications/178574/pengetahuan-sikap-dan-perilaku-masyarakat-dalam-memilih-obat-yang-aman-di-tiga-p (accessed May 7, 2019).

93 Siswati S. Analisis penggunaan antibiotika yang tidak rasional pada balita penderita bukan penumonia di kota Padang. Lembaga Penelitian, Universitas Negeri Padang, 2009 http://ejournal.unp.ac.id/index.php/sainstek/article/view/153 (accessed May 8, 2019).

94 Tandjung H, Wiyono WI, Mpila DA. Pengetahuan dan penggunaan antibiotik secara swamedikasi pada masyarakat di kota Manado. 2021 DOI:10.35799/PHA.10.2021.34044.

95 WHO. Antibiotic Resistance: Multi-Country Public Awareness Survey. *WHO Press* 2015; : 1–51.

96 Widayati A, Suryawati S, de Crespigny C, Hiller JE. Self medication with antibiotics in Yogyakarta City Indonesia: a cross sectional population-based survey. *BMC Research Notes* 2011; 4: 491.

97 Widayati A, Suryawati S, de Crespigny C, Hiller JE. Knowledge and beliefs about antibiotics among people in Yogyakarta City Indonesia: a cross sectional population-based survey. *Antimicrobial Resistance and Infection Control* 2012; 1: 38.

98 Yulia R, Putri R, Wahyudi R. Studi Tingkat Pengetahuan Masyarakat terhadap Penggunaan Antibiotik di Puskesmas Rasimah Ahmad Bukittinggi. *Journal of Pharmaceutical And Sciences* 2019; 2: 43–8.

99 Yuliani NN, Wijaya C, Moeda G. Tingkat pengetahuan masyarakat RW IV Kelurahan Fontein Kota Kupang terhadap penggunaan antibiotik. 2014 https://media.neliti.com/media/publications/259672-tingkat-pengetahuan-masyarakat-rwiv-kelu-a98691f1.pdf (accessed May 10, 2019).

100 Zhang J, Cameron D, Quak SH, *et al.* Rates and determinants of antibiotics and probiotics prescription to children in Asia-Pacific countries. *Beneficial Microbes* 2020; 11: 329–38.
