# Supplementary material for: Optimizing antibiotic use in Indonesia: A systematic review and evidence synthesis to inform opportunities for intervention
Source: Lancet Reg Health Southeast Asia. 2022 May 26;2:100013. doi: 10.1016/j.lansea.2022.05.002 (PMC10305907; doi:10.1016/j.lansea.2022.05.002)
Supplement: Supplementary file 2 [file mmc2.docx]

**Abstrak**

**Latar belakang**

Pendorong utama resistensi antimikroba/*antimicrobial resistance* (AMR) dan keluaran klinis yang buruk adalah penggunaan antibiotik yang kurang optimal meskipun data tentang masalah ini masih kurang di area yang bersumber daya rendah. Kami menelaah berbagai penelitian tentang penggunaan antibiotik sistemik (WHO ATC/DDD kategori J01) untuk kesehatan manusia di Indonesia, dan mensintesis bukti yang ada guna melihat peluang untuk intervensi.

**Metode**

Kami mencari secara sistematis pada lima pangkalan data internasional dan nasional, artikel yang memenuhi kriteria kelayakan ditelaah mitra bestari (*peer-reviewed*), ditulis dalam bahasa Inggris dan Indonesia, serta diterbitkan antara tanggal 1 Januari 2000 sampai 1 Juni 2021, yang mencakup: 1) konsumsi antibiotik; 2) ketepatan peresepan; 3) penatagunaan antimikroba/*antimicrobial stewardship* (AMS); 4) persepsi konsumen dan penyedia layanan. Dua penelaah secara independen memilih artikel dan melakukan ekstraksi data dari artikel yang dipilih. Analisis data dilakukan dengan menggunakan model meta-analisis efek-acak (*random-effect model*) untuk melihat kesesuaian konsumsi dan peresepan, analisis arah efek (*effect direction analysis*) untuk intervensi AMS, serta sintesis kualitatif untuk survei persepsi. (PROSPERO: CRD42019134641)

**Hasil**

Dari 9323 hasil pencarian, kami memasukkan 100 laporan tentang konsumsi antibiotik (20), ketepatan peresepan (49), AMS (13), dan/atau persepsi (25) (8 dikategorikan ke dalam >1 domain). Estimasi gabungan (*pooled estimate*) konsumsi antibiotik adalah 134,8 DDD/100 pasien-hari (KI95 82,5-187,0) untuk pasien rawat inap, dan 121.1 DDD/1000 populasi per hari (KI95 10,4-231,8) untuk pasien rawat jalan. Ceftriakson, levofloksasin, dan ampisilin merupakan antibiotik yang paling banyak dikonsumsi oleh pasien rawat inap; dan amoksisilin, siprofloksasin, and sefadroksil oleh pasien rawat jalan. Estimasi gabungan untuk peresepan yang tepat secara keseluruhan (menurut metode Gyssens) adalah 33,5% (KI95 18,1–53,4) di rumah sakit dan 49,4% (KI95 23,7–75,4) di fasilitas kesehatan primer. Estimasi gabungan untuk peresepan yang tepat (menurut panduan) di rumah sakit adalah 99,7% (KI95 97,4–100) untuk indikasi, 84,9% (KI95 38,5–98,0) untuk pilihan obat, dan 6,1% (KI95 0,2–63,2) untuk kesesuaian secara keseluruhan. Pada fasilitas kesehatan primer, estimasi gabungannya adalah 98,9% (KI95 60,9–100%) untuk indikasi, 82,6% (KI95 50,5–95,7) untuk pilihan obat, dan 10,5 (KI95 0,8–62,6) untuk kesesuaian secara keseluruhan. Studi-studi yang mengevaluasi gabungan beberapa intervensi AMS menunjukkan efek positif terhadap konsumsi antibiotik, ketepatan resep, kepatuhan terhadap panduan, dan keluaran pasien. Tema utama yang diidentifikasi dalam survei persepsi adalah kurangnya pengetahuan antibiotik di antara konsumen dan penggunaan antibiotik tanpa resep.

**Interpretasi**

Strategi intervensi spesifik konteks sangat dibutuhkan untuk meningkatkan penggunaan antibiotik yang tepat di rumah sakit dan masyarakat di Indonesia, dengan kesenjangan bukti kritis tentang penyedia layanan kesehatan swasta dan informal.

**Pendanaan**

Wellcome Trust Africa Asia Programme Vietnam.
